# Supplementary material for: Validation of chronic obstructive pulmonary disease recording in the Clinical Practice Research Datalink (CPRD-GOLD)
Source: BMJ Open. 2014 Jul 22;4(7):e005540. doi: 10.1136/bmjopen-2014-005540 (PMC4120321; doi:10.1136/bmjopen-2014-005540)
Supplement: Author's manuscript [file bmjopen-2014-005540.draft_revisions.pdf]

Validation of Chronic Obstructive Pulmonary Disease (COPD) recording in the Clinical Practice Research Datalink (CPRD-GOLD)

Journal:	*BMJ Open*
Manuscript ID:	bmjopen-2014-005540
Article Type:	Research
Date Submitted by the Author:	23-Apr-2014

Complete List of Authors:	Quint, Jennifer; London School of Hygeine and Tropical Medicine, Epidemiology<br>M�llerova, Hana; GlaxoSmithKline, Epidemiology Department<br>DiSantostefano, Rachael; GlaxoSmithKline, Worldwide Epidemiology<br>Forbes, Harriet; London school of Hygiene and Tropical Medicine, Epidemiology Department<br>Eaton, Susan; MHRA, Clincal Practice Research Datalink Group<br>Hurst, John; UCL Medical School,, Academic Unit of Respiratory Medicine<br>Davis, Kourtney<br>Smeeth, Liam; London School of Hygiene and Tropical Medicine, Epidemiology and Population Health

**Primary Subject Heading**:	Respiratory medicine
Secondary Subject Heading:	Epidemiology
Keywords:	RESPIRATORY MEDICINE (see Thoracic Medicine), THORACIC MEDICINE, Chronic airways disease < THORACIC MEDICINE, Epidemiology < THORACIC MEDICINE

Validation of Chronic Obstructive Pulmonary Disease (COPD) recording in the Clinical Practice Research Datalink (CPRD-GOLD)

Jennifer K Quint[1], Hana Müllerova[2], Rachael L DiSantostefano[3], Harriet Forbes[1], Susan Eaton[4], John R Hurst[5], Kourtney Davis[2], Liam Smeeth[1].

[1]Department of Non-communicable disease Epidemiology, London School of Hygiene and Tropical Medicine, London, UK
[2] Respiratory Epidemiology, GlaxoSmithKline R&D, Uxbridge, UK
[3] Respiratory Epidemiology, GlaxoSmithKline R&D, RTP, NC USA
[4] Clinical Practice Research Datalink Group, Medicines and Healthcare products Regulatory Agency, London, UK
[5]UCL Respiratory Medicine, Royal Free Campus, University College London Medical School, London, UK

Corresponding author:
Dr. Jennifer K Quint
Department of Non-communicable disease Epidemiology
London School of Hygiene and Tropical Medicine
Keppel Street, London WC1E 7HT
Tel: 0207 927 2622
Email: **Jennifer.quint@lshtm.ac.uk**

Keywords: COPD, diagnosis of COPD, validation, spirometry, electronic health records

Word count: 3448

For peer review only - http://bmjopen.bmj.com/site/about/guidelines.xhtml

ABSTRACT (252 words)

Background: The optimal method of identifying people with COPD from primary care records is not known. We assessed the accuracy of different approaches using the Clinical Practice Research Datalink (CPRD).

Methods: Participants were registered with a CPRD practice between 1st January 2004 and 31st December 2012. Individuals were selected for ≥1 of eight algorithms to identify people with COPD. General practitioners were sent a brief questionnaire and additional evidence to support a COPD diagnosis was requested. All information received was reviewed independently by two respiratory physicians whose opinion was taken as gold standard. The primary measure of accuracy was the positive predictive value (PPV); the proportion of people identified by each algorithm for whom COPD was confirmed.

Results: Nine hundred and fifty one questionnaires were sent and 738 (78%) returned. After quality control, 696 (73.2%) patients were included in the final analysis. All four algorithms including a specific COPD diagnostic code performed well. Using a diagnostic code alone the PPV was 86.5% (77.5% to 92.3%) while requiring a diagnosis plus spirometry plus specific medication the PPV was slightly higher at 89.4% (80.7% to 94.5%) but reduced case numbers by 10%. Algorithms without specific diagnostic codes had low PPVs (range 12.2% to 44.4%).

Conclusion: Patients with COPD can be accurately identified from UK primary care records using specific diagnostic codes. Requiring spirometry or COPD medications only marginally improved accuracy. The high accuracy applies since the introduction of an incentivised disease register for COPD as part of QOF (Quality and Outcomes Framework) in 2004.

Article Summary: This paper investigates how individuals with COPD can be accurately identified in electronic health records using specific COPD codes alone without additional

For peer review only - http://bmjopen.bmj.com/site/about/guidelines.xhtml

information (i.e. presence of spirometry or respiratory medications). This information is essential for appropriate disease management, epidemiological research of COPD and other conditions, where COPD is an important co-morbidity.

Strengths and limitations of this study:

- We have shown that the presence of a specific COPD Read code alone is sufficient to identify patients with COPD from electronic health records. Minimal precision lost by not including spirometry and medications in the algorithm, allows an increase in the number of individuals who can potentially be included in a study by up to 10%.
- We were able to investigate both the accuracy of algorithms when identifying COPD patients within the CPRD, and accuracy of the actual GP diagnosis of COPD.
- The amount of missing data among the responding questionnaires was low, suggesting reasonable data quality.
- Although the overall response rate for this study was acceptable (77.6%), the proportion of questionnaires accompanied with additional evidence allowing for adjudication was lower.

No additional data are available. We would be happy to provide the ISAC protocol to reviewers if required.

For peer review only - http://bmjopen.bmj.com/site/about/guidelines.xhtml

INTRODUCTION

Chronic obstructive pulmonary disease (COPD) represents an enormous health burden worldwide. Currently COPD is the 4th leading cause of death and is predicted to become the 3rd by 2020.[1] There are approximately 835,000 people diagnosed with COPD in the UK and an estimated 2,200,000 people remain undiagnosed.[2]

Electronic health records in the UK provide an excellent resource in which to study COPD as they offer a large cohort size, the presence of disease severity indicators and long-term follow-up information on a patient's integrated medical history. Although multiple studies have been undertaken to investigate various aspects of COPD over the last 10 years in several electronic health record databases, there is no standard definition used to identify COPD in large databases and codelists used to identify patients with COPD vary by author. Over ten years ago, the diagnosis of COPD was validated in the Clinical Practice Research Datalink (CPRD-GOLD, formerly GPRD) using OXMIS codes.[3] This coding system is now obsolete in CPRD and Read codes are used as standard.

There is no single diagnostic test for COPD. The diagnosis of COPD relies on clinical judgement based on a combination of history, physical examination and confirmation of the presence of airflow obstruction using spirometry.[4] When retrospectively evaluating the accuracy of a COPD diagnosis, commonly used COPD definitions may misclassify patients as having COPD. Using multiple diagnostic codes in combination with pharmacy may improve the accuracy of identification of COPD patients.[5] Further, over the past 10 years the definition of COPD in clinical practice has evolved leading to changes in how COPD is diagnosed and the diagnosis recorded. With the introduction of the Quality and Outcomes Framework (QOF) codes for COPD in 2004 in England and Wales there are more codes available to identify COPD then there were previously. Evaluation of airflow limitation using

For peer review only - http://bmjopen.bmj.com/site/about/guidelines.xhtml

spirometry is used as standard to confirm COPD diagnosis and severity of COPD is part of the annual COPD patient review.[3,6] However, even spirometry if incorrectly performed or interpreted can lead to misdiagnosis (both over and under diagnosis of COPD) in approximately 20% of cases. It is also recognised that some subgroups of individuals (e.g. women and individuals under 50 and over 80) are less likely to have spirometry measured.[6]

This study aimed to improve our ability to identify patients with COPD within electronic health records. We used the CPRD, a widely used collection of computerised medical records which is commonly used for clinical and research purposes. CPRD is comparable to other electronic health record databases used in the UK. Our objective was to test the accuracy of different definitions of COPD in the CPRD using Positive Predictive Value (PPV), by comparing the database records with additional information provided by General Practitioners (GPs). This work is important for epidemiological research in COPD and diseases where COPD is an important co-morbidity as well as for clinical practice.

METHODS

Dataset

CPRD is the world's largest validated computerized database of anonymized longitudinal medical records for primary care. [7,8] Data comprise approximately 14 million patients with around 5.4 million of these being currently alive and registered from 660 primary care practices spread throughout the UK. Records are derived from a widely used GP software system and contain complete prescribing and coded diagnostic and clinical information as well as information on tests requested, laboratory results and referrals made at or following on from each consultation. [9]

For peer review only - http://bmjopen.bmj.com/site/about/guidelines.xhtml

Codelists and algorithms

Lists of medical codes (Read codes) specific and non-specific for COPD were created prior to the study initiation. Specific COPD codes consisted of codes listing either COPD or COPD specific entities of emphysema (see supplementary material). Non-specific codes consisted of a variety of lung diseases that could potentially be COPD e.g. chronic bronchitis. Combinations of codelists and additional material in the form of the presence of spirometry or COPD medications (see supplementary material) were used to make up the eight algorithms. The first four definitions required a specific COPD diagnosis code, with the first three requiring additional documentation (e.g., medication and/or spirometry). The other four definitions required non-specific bronchitis or respiratory symptom codes, with the least specific definition requiring only respiratory symptom codes. Details regarding each algorithm can be found in the supplementary material. Briefly, the eight algorithms were defined as follows, from the expected most specific to most sensitive construct:

1. Specific COPD code and more than one prescription of a COPD medication and presence of spirometry (**COPD Code + spirometry + COPD medication)**
2. Specific COPD code and presence of spirometry (**COPD Code + spirometry)**
3. Specific COPD code and more than one prescription of a COPD medication (**COPD Code + COPD medication)**
4. Specific COPD code only (**COPD Code only)**
5. Non-specific bronchitis code and more than one prescription of a COPD medication (**Bronchitis + COPD medication)**
6. Non-specific bronchitis code only (**Bronchitis only)**
7. Respiratory symptoms and presence of spirometry. Respiratory symptoms consisted of persistent cough, sputum production, or dyspnoea. (**Symptoms + spirometry)**
8. Respiratory symptom definition only (**Symptoms only)**

For peer review only - http://bmjopen.bmj.com/site/about/guidelines.xhtml

The presence of spirometry for inclusion in the algorithm was based on an existence of a record of a specific value or a Read code for spirometry rather than examining the actual value. We were able to assess interpretation of spirometry in the subset of patients who had flow volume loops or clinic letters attached and where actual FEV_1 and FVC values were available in CPRD.

Study Population

The study population consisted of a random sample of individuals selected from all subjects registered in CPRD with cohort entry being on or after the 1st January 2004. At cohort entry, people included had to be: over 35 years old, with at least 1 year historical data, and a possible diagnosis of COPD defined as evidence of having ever smoked and a record of a least one specific or possible COPD code or respiratory symptoms suggestive of COPD. The presence of the algorithms was ascertained during a period between patient cohort entry and the 31st December 2012. Patients had to be alive within 4 months of the last collection date of CPRD data for the January 2013 data build for inclusion in the analysis so that CPRD could access their medical records and additional information. For the main analysis, a patient could contribute to one algorithm only. It was possible for an individual to be eligible for more than one algorithm depending upon the codes used in their medical record over the study period. Individuals were randomly selected from the algorithm with the fewest number of participants first and then removed from the cohort so they could not be selected for another algorithm.

CPRD mailed a short, structured questionnaire to GPs in charge of randomly selected patients requesting confirmation of COPD status as well as any available specific information from the individual's medical record including spirometry print outs and hospital respiratory outpatient letters (see supplementary material). Data were "twice encrypted" within CPRD to ensure anonymity; firstly between practices and CPRD and secondly from CPRD to

For peer review only - http://bmjopen.bmj.com/site/about/guidelines.xhtml

researchers. In the questionnaire the GP was asked whether or not the patient had a diagnosis of COPD, what that diagnosis was based on, whether or not the patient had seen a respiratory physician and if they had any other respiratory diagnoses. A pilot set of 20 questionnaires were sent to GPs to assess the quality of the questionnaire. In total, 951 questionnaires were sent out assuming an 80% response rate.

Primary outcome

The primary outcome was identification of a diagnosis of COPD according to the pre-defined eight algorithms. The gold standard for the diagnosis of COPD was the decision made after respiratory physicians independently reviewed the evidence (questionnaire response with or without- additional evidence). Where they did not agree, a 3rd independent physician decided. Additionally, GP diagnosis of COPD was validated in a subset of patients where the GP provided supportive information including spirometry print-outs and hospital letters. This also allowed review of spirometry interpretation in some cases.

Analysis

The primary analysis focused on the accuracy of identification of a COPD diagnosis in each of the pre-defined algorithms as defined by positive predicted value (PPV); i.e. the proportion of “true positives” (individuals with COPD) in each algorithm as determined by the gold standard. In addition, within each algorithm, where additional information was provided (lung function, hospital clinic letters), we calculated the accuracy of the GP diagnosis of COPD relative to the gold standard. This allowed review of spirometry interpretation in some cases.

We assessed the impact of commonly occurring co-morbidities on the accuracy of the pre-specified algorithms stratifying for cardiovascular co-morbidity, previous asthma diagnosis, smoking status and, where possible, Global Initiative for Chronic Obstructive Lung Disease (GOLD) staging of airflow limitation severity.[10] Cardiovascular co-morbidity included

For peer review only - http://bmjopen.bmj.com/site/about/guidelines.xhtml

angina, history of myocardial infarction, previous coronary artery bypass graft / percutaneous coronary intervention or heart failure, but not hypertension due to its lack of overlap of symptoms that could mimic COPD. All covariates for stratification analysis were derived from information available up to cohort entry.

As a post hoc analysis individuals were eligible to be placed into multiple algorithms where possible, and the PPV was calculated for all individuals who had a specific COPD code compared to those with a specific COPD code and additional information (either spirometry or a COPD medication).

Assessment of possible trends in COPD diagnosis recording were also evaluated, including temporal trends in codes used and diagnostic specificity from 2004 to 2011. In addition, we compared our specific COPD codes to those recommended for use by QOF (see supplementary material); [11,12] H31% (excluding H3101 (smokers cough), H31y0 (chronic tracheitis) and H3122 (acute exacerbation of COPD)) H32% H36-H3z (excluding H3y0 and H3y1).

Sample size calculation

Our sample size for each algorithm was chosen to achieve accuracy of the true positives or PPV ±0.08 based on the reviewing physician judgment as the gold standard. Assuming an estimated PPV of 0.85 for any one algorithm, we required a sample of at least 77 individuals in each algorithm to achieve the desired accuracy (95% CI ±0.08). All analyses were performed using STATA 13.

Ethics approval was obtained from ISAC (the Independent Scientific Advisory Committee overseeing CPRD); protocol 12_065 and the LSHTM ethics committee.

For peer review only

RESULTS

Nine hundred and fifty one questionnaires were sent to GPs (see **Figure 1** for patient selection). Of those, 738 (77.6%) were returned, 704 (74.0%) met quality control standards and were not duplicates, and 696 (73.2%) could be included in the final analysis (8 had "uncertain" COPD diagnosis and no supporting documentation and were therefore excluded).

Among those included in the final analysis, additional evidence for the diagnosis of COPD was available for 272 patients. This represented 39.1% of the total study population, or 67.7% of the 402 patients with a confirmed COPD diagnosis in the study.

Overall, irrespective of the qualifying algorithm, 402 patients (57.8%) were considered to have a diagnosis of COPD based on reviewing physician judgment. **Table 1** shows the characteristics of the 696 patients included in the final analysis who were considered to possibly have COPD based on the inclusion criteria. On average, patients were in their mid-60s to early 70s across all algorithms. Approximately two-thirds of them were current smokers and one-quarter had a history of asthma. Generally, there were fewer patients with supporting information and cardiovascular co-morbidity in the less specific algorithms (4-8).

For peer review only

For peer review only - http://bmjopen.bmj.com/site/about/guidelines.xhtml

Table 1: Characteristics of the 696 patients included in the final study analysis

Algorithm		COPD Code + spirometry + COPD medication	COPD Code + spirometry	COPD Code + COPD medication	COPD Code only	Bronchitis + COPD medication	Bronchitis only	Symptoms + spirometry	Symptoms only
**Number of individuals (%)**		85 (100)	79 (100)	88 (100)	89 (100)	98 (100)	84 (100)	83 (100)	90 (100)
**Number (%) with supporting info**		46 (54.1)	44 (55.7)	48 (54.5)	40 (44.9)	32 (32.7)	18 (21.4)	30 (36.1)	14 (15.6)
**Mean age (sd)**		68.7 (11.3)	68.3 (11.7)	71.8 (10.5)	71.1 (10.4)	68.5 (13.1)	67.8 (13.4)	65.9 (11.9)	63.4 (14.1)
**Male (%)**		45 (52.9)	41 (51.9)	40 (45.5)	44 (49.4)	31 (31.6)	29 (34.5)	43 (51.8)	47 (52.2)
**Current smoker (%)**		49 (57.7)	50 (63.3)	55 (62.5)	47 (52.8)	66 (67.4)	61 (72.6)	48 (57.8)	58 (64.4)
**GOLD stage* (n=465)**	**1**	13 (16.3)	14 (18.0)	13 (15.9)	16 (20.5)	17 (28.8)	13 (35.1)	8 (20.0)	4 (36.4)
	**2**	43 (53.8)	48 (61.5)	41 (50.0)	46 (59.0)	31(52.5)	13 (35.1)	22 (55.0)	5 (45.5)
	**3**	18 (22.5)	13 (16.7)	22 (26.8)	12 (15.4)	9 (15.3)	8 (21.6)	8 (20.0)	2 (18.2)
	**4**	6 (7.5)	3 (3.9)	6 (7.3)	4 (5.1)	2 (3.4)	3 (8.1)	2 (5.0)	0 (0.0)
**History of cardiovascular disease**		20 (23.5)	22 (27.8)	17 (19.3)	18 (20.2)	20 (20.4)	17 (20.2)	14 (16.9)	9 (10.0)
**History of asthma**		18 (21.2)	20 (25.3)	16 (18.2)	15 (16.9)	27 (27.6)	23 (27.4)	19 (22.9)	23 (25.6)
**Mean BMI (sd) (n=575)**		N=83<br>27.5 (5.4)	N=78<br>26.7 (5.8)	N=86<br>26.4 (4.7)	N=87<br>27.8 (5.4)	N=98<br>27.4 (5.1)	N=85<br>28.1 (5.0)	N=41<br>27.1 (4.5)	N=17<br>27.6 (4.7)

*GOLD staging was ascertained from CPRD records or from supplementary information provided by GPs.

For peer review only - http://bmjopen.bmj.com/site/about/guidelines.xhtml

The number of patients diagnosed with COPD confirmed by the gold standard and the PPV for each algorithm is given in **Table 2**. The PPV was greatest for algorithms 1-4. Further data is available in the supplementary material regarding the effect of co-morbidities (**Table 4**), smoking status (**Table 5**) and GOLD staging (**Table 6**) on the performance of each algorithm.

Table 2: The PPV and proportion of patients diagnosed with COPD within each algorithm

Algorithm	Number of questionnaires sent out (n=951)	Number evaluable returned (n=696) (%)	Number with confirmed COPD	PPV and 95% CI
**COPD Code + spirometry + COPD medication**	119	85 (71.4)	76	89.4, 80.7-94.5
**COPD Code + spirometry**	119	79 (66.4)	67	83.8, 73.7-90.4
**COPD Code + COPD medication**	119	88 (73.9)	77	87.5, 78.6-93.0
**COPD Code only**	119	89 (74.8)	77	86.5, 77.5-92.3
**Bronchitis + COPD medication**	119	98 (82.4)	44	44.4, 34.8-54.5
**Bronchitis only**	119	84 (70.6)	26	29.5, 20.8-40.1
**Symptoms + spirometry**	119	83 (69.7)	37	43.5, 33.2-54.4
**Symptoms only**	118	90 (75.6)	11	12.2, 6.8-20.9

In a subset of 272 patients where additional evidence was available (in the form of spirometry print outs or hospital outpatient letters), we assessed accuracy of GP diagnosis of COPD. Overall, the PPV in this group was 95.0% (91.1-97.2), This is broken down by algorithm in **Table 3**. While the presence of supporting evidence improved the PPV in each group, algorithms 1-4 were still most accurate.

For peer review only - http://bmjopen.bmj.com/site/about/guidelines.xhtml

Table 3: PPV by algorithm where evidence was available to assess GP compared to physician diagnosis of COPD

Algorithm	Total number with evidence (N=272)	Number of patients with COPD confirmed by GOLD standard (N=220)	Number with COPD according to GP	PPV and 95% CI
**COPD Code + spirometry + COPD medication**	46	46	46	100
**COPD Code + spirometry**	44	35	33	94.3, 82.4-98.9
**COPD Code + COPD medication**	48	43	41	95.3, 82.4-98.9
**COPD Code only**	40	34	33	97.1, 80.2-99.6
**Bronchitis + COPD medication**	32	21	19	90.5, 66.0-97.9
**Bronchitis only**	18	12	11	91.7, 49.9-99.2
**Symptoms + spirometry**	30	21	20	95.2, 69.1-99.4
**Symptoms only**	14	8	6	75.0, 27.6-95.9

Post-hoc analysis

We repeated the analysis allowing individuals to be put into more than one algorithm and tested the PPV of COPD by algorithm relative to the gold standard where individuals were identified using only the presence of a specific COPD code (n=457) the PPV was identical to if they had a COPD code and evidence either in the form of spirometry or COPD medication prescription (n=454); PPV 83.0% (79.0-86.0) for both. In the majority of cases, where a specific COPD code had been assigned, there was additional evidence. Only 3 individuals had a specific COPD code with no additional evidence.

DISCUSSION

We tested the accuracy of eight different algorithms for identifying COPD within the CPRD among patients with suspected COPD (e.g., >35 years of age, smoking history, and recording of respiratory symptoms or COPD codes). The physician reviewer consensus was the GOLD standard. The best performing algorithm allowed an accurate ascertainment of 90% of patients as diagnosed with COPD. This consisted of a combination of a specific COPD code, more than one prescription of a COPD medication and spirometry (PPV 89.4,

For peer review only - http://bmjopen.bmj.com/site/about/guidelines.xhtml

95% CI: 80.7-94.5). The worst performing algorithm was represented by presence of respiratory symptoms only (PPV12.2, 95%CI: 6.8-20.9). We found that any algorithm containing a specific COPD code performed better than those without (Algorithms 1-4). In a post hoc analysis, where we allowed individuals to populate more than one algorithm, we established that the use of additional information such as spirometry or medications in an algorithm to a specific COPD Read code alone did not increase the PPV. This suggests that the presence of a specific COPD Read code alone is sufficient to accurately identify patients with COPD from the database. Some study protocols require the presence of obstructive spirometry for identification of COPD patients; however, this study demonstrates that it may be unnecessary. This is particularly important as certain groups of individuals are less likely to have spirometry, namely women and patients <50 and >80 years of age. [6] This is also important as the minimal precision lost by not including spirometry and medications in the algorithm, allows an increase in the number of individuals who can potentially be included in a study. Using the whole of CPRD, we identified individuals with COPD using a specific COPD code only compared to a specific code plus medication and spirometry and found an increase in potential sample size for study of 10% using a specific COPD code only.

One of the advantages of this study was our ability to investigate both the accuracy of algorithms when identifying COPD patients within the CPRD, and accuracy of the actual GP diagnosis of COPD. When validating the GP diagnosis of COPD with a respiratory physician diagnosis as gold standard based on extra evidence provided by the GP, there was improved accuracy (PPV) across all algorithms, with algorithms 1-4 again performing best. This suggests that additional evidence is collected when the GP is reasonably certain that the patient has COPD. There was good concordance between GP and physician diagnosis suggesting respiratory consultant validation is not always needed. Where there was disagreement, this was usually because lung function did not meet criteria for COPD.

For peer review only

For peer review only - http://bmjopen.bmj.com/site/about/guidelines.xhtml

We found that the diagnostic accuracy of COPD decreased across all algorithms when patients also had a diagnosis of cardiovascular disease or asthma (Table 4 online supplement). When patients had a concomitant diagnosis of asthma, the presence of spirometry was particularly important to improve accuracy of COPD diagnosis. However, stratification led to smaller sample sizes, which could have impacted the precision of estimates. Unsurprisingly, the addition of the use of any inhaled COPD medication to the algorithm did not improve precision, most likely due to the overlap in medications used to treat both asthma and COPD (see online supplement). The algorithm accuracy was not affected by smoking status (current vs. ex-smoker) (Table 5 online supplement). We only included current or former smokers in our analysis, and cannot be sure of the validity of the results in a patient who has never smoked. Certainly in the UK, the majority of COPD is related to tobacco smoking and we hypothesized that fixed airflow obstruction in a non-smoker would most likely be due to chronic asthma.

When considering the severity of COPD by GOLD classification, algorithms 2 and 3, i.e. a specific COPD code and spirometry or COPD medications had the greatest accuracy for patients with mild disease (GOLD stage 1) (Table 6 online supplement). PPV increased with increasing disease severity. PPV increases with disease prevalence, and the prevalence of COPD increased moving from algorithm 8 to algorithm 1.

We considered the timing of diagnosis in view of an increased uptake of spirometry in primary care in more recent years and changes in QOF requirements over time during our study period (Table 7 online supplement). We found that algorithms 1-4 still had the greatest accuracy, but the PPV estimates were better for the post 2008 period than pre 2008 period (see supplementary material). Non-specific bronchitis codes and symptom codes were more likely to be used before 2008 rather than post. This may require consideration when developing retrospective cohorts for analysis and otherwise suggests QOF has had a

For peer review only - http://bmjopen.bmj.com/site/about/guidelines.xhtml

positive effect on the consistency of codes used for COPD diagnoses. However, our codes were more specific than QOF codes and some of the codes included in QOF were not included in our specific COPD codelist, but were included in the non-specific bronchitis codes. While we cannot comment on the accuracy of QOF codes, it is important to highlight that that some QOF codes are not disease specific and may not be a good way of identifying COPD patients from electronic health records as inevitably people without COPD will be included. It is also important to highlight that Read codes change over time with new codes added and some removed, and this needs consideration when identifying people with COPD.

There are limited COPD validation studies in electronic health records published in the literature to which we can compare our study. Soriano validated COPD in the GPRD in 2001, when OXMIS codes were still in use. [3] A Swedish study using a Swedish inpatient registry used ICD-9 and ICD-10 codes and identified COPD patients with similar accuracy.[13] A Canadian study in the Canadian primary care sentinel surveillance network used algorithms to identify several long term conditions and also had a PPV similar to ours for COPD [14]. However, all of these studies used different codes, algorithms and databases.

Our analysis has several limitations. Although the overall response rate for this study was acceptable (77.6%), the proportion of questionnaires accompanied with additional evidence allowing for adjudication was rather low. We chose PPV as the measure of accuracy in this study to allow us to determine the probability that a patient had COPD from their electronic health record. PPV is correlated with disease prevalence, and although it is strongly related to specificity, the actual estimates of specificity, sensitivity and negative predictive value cannot be determined from our data. Further, GP practices are self selecting with respect to their contribution to CPRD, however those practices appear to be representative of the UK population. Very few patients within contributing practices refuse to participate at an

For peer review only - http://bmjopen.bmj.com/site/about/guidelines.xhtml

individual level and this is not thought to bias the results. While CPRD is representative of the general population, as with all validation studies that require a response, we cannot be sure that our sample is representative of GPs who have not responded, although there is unlikely to be any difference. The amount of missing data among the responding questionnaires was low, suggesting reasonable data quality. One of the other limitations of this study is that patients had to be alive to be included; however it is unlikely that coding would be different for individuals who are no longer alive.

The algorithm that consisted of a specific COPD code, COPD medication and spirometry had the highest PPV; however the PPV was almost as high when a specific COPD code alone was used. The poorest performing algorithms were those that involved bronchitis codes or respiratory symptoms; we would not recommend using these algorithms to identify COPD patients. In conclusion, we have shown that the presence of a specific COPD Read code alone is sufficient to identify patients with COPD from electronic health records. Minimal precision lost by not including spirometry and medications in the algorithm, allows an increase in the number of individuals who can potentially be included in a study by up to 10%. However by not including spirometry in the definition, the ability to stage COPD according to GOLD stages may not be possible for all COPD patients included in a study.

For peer review only

For peer review only - http://bmjopen.bmj.com/site/about/guidelines.xhtml

Acknowledgments: none

Funding: This project was funded jointly by GSK and the MRC. JKQ is funded on a MRC Population Health Scientist Fellowship. This work was part funded by a MRC Industry Partnership award (grant number G0902135).

Contributorship statement: JKQ, HM, RLD, SE, KD and LS contributed to the conception and design, JKQ, HM, JH, to acquisition and analysis, JKQ, HM, RLD, HF, SE, JH, KD and LS to interpretation of data, and JKQ, HM, RLD, HF, SE, JH, KD and LS to the drafting of the manuscript. JKQ is responsible for the overall content as guarantor.

Competing interests: There are no competing interests

Data Sharing Statement: No additional data are available. We would be happy to provide the ISAC protocol to reviewers if required.

For peer review only

Figure 1: Study population

REFERENCES

1. National Statistics. www.statistics.gov.uk date accessed June 2013.

2. Shahab LM, Jarvis MJ, Britton J, et al. Prevalence, diagnosis and relation to tobacco dependence of chronic obstructive pulmonary disease in a nationally representative population sample *Thorax* 2006;61(12):1043

3. Soriano JB, Maier WC, Visick G, et al. Validation of general practitioner-diagnosed COPD in the UK General Practice. *European Journal of Epidemiology* 2001;17:1075–1080

4. NICE guideline COPD http://guidance.nice.org.uk/CG101/QuickRefGuide/pdf/English date accessed January 2014.

5. Cooke CR, Joo MJ, Anderson SM et al. The validity of using ICD-9 codes and pharmacy records to identify patients with chronic obstructive pulmonary disease. *BMC Health Services Research* 2011;11:37

6. Smith CJP, Gribbin J, Challen KB, et al. The impact of the 2004 NICE guideline and 2003General Medical Services contract on COPD in primary care in the UK. *Q J Med* 2008;101:145–153.

7. CPRD http://www.cprd.com (accessed February 28,2013)

For peer review only - http://bmjopen.bmj.com/site/about/guidelines.xhtml

8. Williams T, VanStaa T, Padmanabhan S et al. Recent advances in utility and use of the General Practice Research Database as an example of a UK Primary Care Data resource. *Ther Adv Drug Saf* 2012;3:88–99.

9. Tate AR, Beloff N, Al-Radwan B et al. Exploiting the potential of large databases of electronic health records for research using rapid search algorithms and an intuitive query interface. *J Am Med Inform Assoc*. 2014;21(2):292-8.

10. GOLD 2011 http://www.goldcopd.org/uploads/users/files/GOLD2011_Summary.pdf accessed Nov 2013.

11. https://mqi.ic.nhs.uk/IndicatorDefaultView.aspx?ref=1.09.03.07 (QOF COPD). Accessed Feb 2014.

12. http://www.nbmedical.com/pdf/keep_simple_qof_2014-2014.pdf accessed Feb 2014

13. Inghammar M, Engström G, Löfdahl C-G et al. Validation of a COPD diagnosis from the Swedish Inpatient Registry *Scandinavian Journal of Public Health*, 2012;40: 773–776

14. Kadhim-Saleh A, Green M, WilliamsonT et al. Validation of the Diagnostic Algorithms for 5 Chronic Conditions in the Canadian Primary Care Sentinel Surveillance Network (CPCSSN): A Kingston Practice-based Research Network (PBRN) Report. *J Am Board Fam Med* 2013;26: 159–167

For peer review only - http://bmjopen.bmj.com/site/about/guidelines.xhtml

Figure 1: Study sample

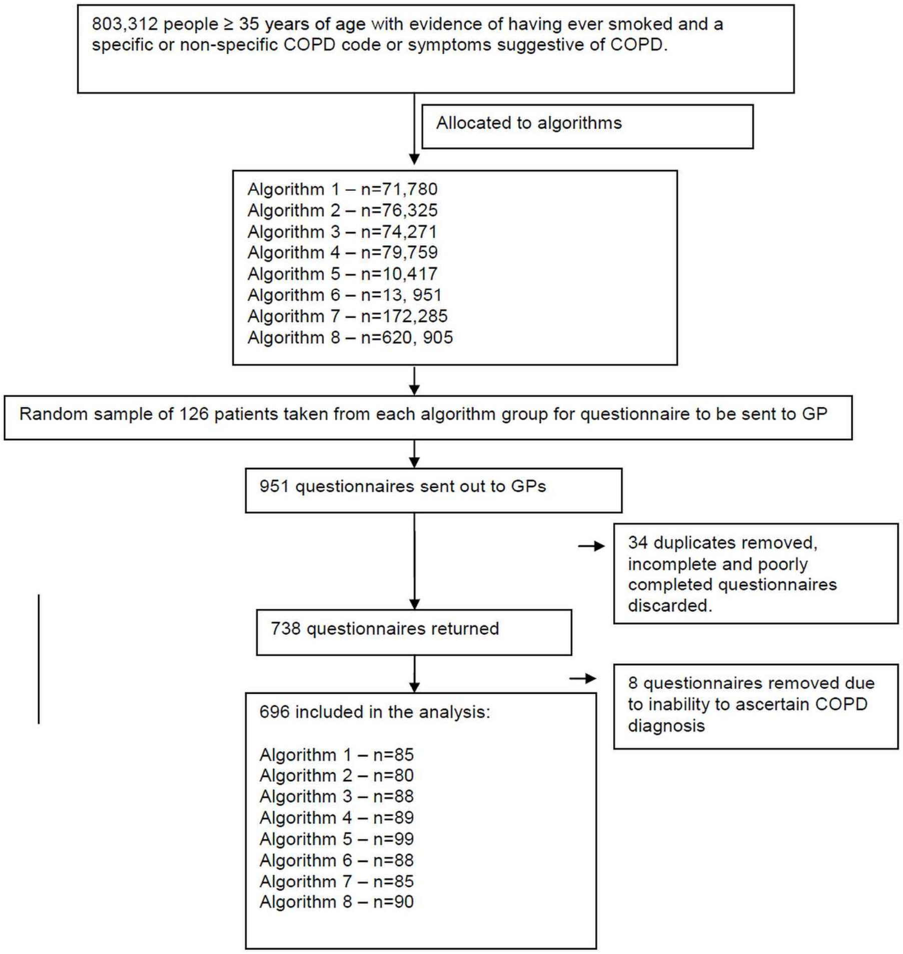

228x248mm (300 x 300 DPI)

Supplementary information

Algorithms

1. Specific COPD code and more than one prescription of a COPD medication and presence of spirometry. The first COPD medication had to be prescribed within 4 weeks of the COPD diagnostic code.
2. Specific COPD code and presence of spirometry
3. Specific COPD code and more than one prescription of a COPD medication. The first COPD medication had to be prescribed within 4 weeks of the COPD diagnostic code.
4. Specific COPD code only
5. Non-specific bronchitis codes and more than one prescription of a COPD medication. The first COPD medication had to be prescribed within 4 weeks of the COPD diagnostic code.
6. Non-specific bronchitis codes only
7. Respiratory symptoms and presence of spirometry. Respiratory symptoms consisted of persistent cough, sputum production, or dyspnoea
8. Respiratory symptom definition only

For peer review only

Specific COPD codes

medcode	term
18476	COPD follow-up
45771	Chronic obstructive pulmonary disease does not disturb sleep
4084	Airways obstructn irreversible
794	Emphysema
998	Chronic obstructive airways disease
1001	Chronic obstructive pulmonary disease
5710	Chronic obstructive airways disease NOS
9520	Chronic obstructive pulmonary disease monitoring
9876	Severe chronic obstructive pulmonary disease
10802	Moderate chronic obstructive pulmonary disease
10863	Mild chronic obstructive pulmonary disease
10980	Centrilobular emphysema
11287	Chronic obstructive pulmonary disease annual review
14798	Emphysematous bronchitis
18621	Chronic obstructive pulmonary disease follow-up
18792	Chronic obstructive pulmonary disease monitoring admin
23492	Chronic bullous emphysema NOS
26018	Chronic obstructive pulmonary disease monitoring by nurse
26306	Chronic bullous emphysema
28755	Chronic obstructive pulmonary disease monitoring 1st letter
33450	Emphysema NOS
34202	Chronic obstructive pulmonary disease monitoring 2nd letter
34215	Chronic obstructive pulmonary disease monitoring 3rd letter
37247	Chronic obstructive pulmonary disease NOS
37371	Chronic obstructive pulmonary disease monitoring due
44525	Obstructive chronic bronchitis NOS
45998	Chronic obstructive pulmonary disease monitoring by doctor
93568	Very severe chronic obstructive pulmonary disease
12166	Other specified chronic obstructive airways disease

38074 Chronic obstructive pulmonary disease monitor phone invite
42258 Chronic obstructive pulmonary disease monitoring verb invite
42313 Health education - chronic obstructive pulmonary disease
45770 Chronic obstructive pulmonary disease disturbs sleep
45777 Chronic obstructive pulmonary disease clini management plan

COPD medication codes

prodcode	productname
8	salbutamol 100micrograms/dose inhaler
17	salbutamol 100micrograms/dose inhaler cfc free
31	ventolin 100microgram/inhalation inhalation powder (glaxo wellcome uk ltd)
38	beclometasone 100micrograms/dose inhaler
99	becotide 100 inhaler (glaxosmithkline uk ltd)
218	aminophylline 100 mg cap
235	bricanyl 250micrograms/dose inhaler (astrazeneca uk ltd)
273	theophylline 200 mg cap
282	salbutamol 2mg/5ml oral solution sugar free
454	pulmicort 200microgram inhaler (astrazeneca uk ltd)
465	salmeterol 25micrograms/dose inhaler
510	ventolin 5mg/ml respirator solution (glaxosmithkline uk ltd)
549	serevent 25micrograms/dose inhaler (glaxosmithkline uk ltd)
555	aminophylline 225mg modified-release tablets
590	phyllocontin continus 225mg tablets (napp pharmaceuticals ltd)
638	seretide 250 accuhaler (glaxosmithkline uk ltd)
665	seretide 100 accuhaler (glaxosmithkline uk ltd)
674	ventolin 2.5mg nebules (glaxosmithkline uk ltd)
696	salbutamol 8mg modified-release capsules
719	salmeterol 50micrograms/dose dry powder inhaler
746	tiotropium 18 microgram capsule
752	carbocisteine 375mg capsules
856	ventolin 2mg/5ml syrup (glaxosmithkline uk ltd)
860	salbutamol 4mg tablets
862	salbulin inhalation powder (3m health care ltd)
863	slo-phyllin 125mg capsule (lipha pharmaceuticals ltd)
879	theophylline 125mg modified-release capsules
880	theophylline 60mg modified-release capsules
881	salbutamol 2mg tablets
882	salbutamol 200microgram inhalation powder capsules
883	becodisks 200microgram disc (allen & hanburys ltd)
895	beclazone 100 easi-breathe inhaler (teva uk ltd)
896	becotide easi-breathe 100microgram/actuation pressurised inhalation (allen & hanburys ltd)
898	ventolin evohaler 100 100microgram/inhalation pressurised inhalation (glaxo wellcome uk ltd)
907	bricanyl turbohaler 500 500microgram turbohaler (astrazeneca uk ltd)
908	pulmicort 400 turbohaler (astrazeneca uk ltd)
909	budesonide 200micrograms/dose inhaler
910	serevent diskhaler 50microgram inhalation powder (glaxo wellcome uk ltd)
911	flixotide accuhaler 250 250microgram/inhalation inhalation powder (allen & hanburys ltd)
947	budesonide 50micrograms/actuation refill canister
956	pulmicort 200 turbohaler (astrazeneca uk ltd)

For peer review only

957 salamol easi-breathe 100microgram/actuation pressurised inhalation (ivax pharmaceuticals uk ltd)
958 ventolin easi-breathe 100microgram/actuation pressurised inhalation (allen & hanburys ltd)
959 budesonide 50micrograms/dose inhaler
960 pulmicort 100 turbohaler (astrazeneca uk ltd)
987 ventolin 4mg tablet (allen & hanburys ltd)
1093 salamol 100microgram/actuation inhalation powder (ivax pharmaceuticals uk ltd)
1097 slo-phyllin 60mg capsule (lipha pharmaceuticals ltd)
1100 beclazone 100 inhaler (teva uk ltd)
1236 becloforte 250micrograms/dose inhaler (glaxosmithkline uk ltd)
1242 beclometasone 250micrograms/dose inhaler
1243 beclazone 250 easi-breathe inhaler (teva uk ltd)
1258 becotide 200 inhaler (glaxosmithkline uk ltd)
1259 beclometasone 200micrograms/dose inhaler
1269 becotide 50microgram/ml nebuliser liquid (allen & hanburys ltd)
1346 salbutamol 0.05mg/ml injection
1406 becotide 50 inhaler (glaxosmithkline uk ltd)
1409 ipratropium bromide 20micrograms/dose inhaler
1410 ipratropium bromide 0.25mg/ml
1411 ipratropium bromide 250micrograms/ml
1412 flixotide 250microgram/actuation inhalation powder (allen & hanburys ltd)
1414 salamol 5mg/2.5ml nebuliser liquid steri-neb unit dose vials (teva uk ltd)
1415 steri-neb ipratropium 250microgram/ml nebuliser liquid (ivax pharmaceuticals uk ltd)
1423 uniphyllin continus 200mg tablets (napp pharmaceuticals ltd)
1424 flixotide 250microgram disc (allen & hanburys ltd)
1426 flixotide 500microgram disc (allen & hanburys ltd)
1518 flixotide 50microgram/actuation inhalation powder (allen & hanburys ltd)
1537 becotide 200microgram rotacaps (glaxosmithkline uk ltd)
1551 beclazone 250 inhaler (teva uk ltd)
1552 becloforte easi-breathe 250microgram/actuation pressurised inhalation (allen & hanburys ltd)
1619 terbutaline 500micrograms/dose dry powder inhaler
1620 terbutaline 250micrograms/dose inhaler
1628 terbutaline 250micrograms/actuation refill canister
1630 salbutamol 2.5mg/2.5ml nebuliser liquid unit dose vials
1635 salbuvent 2mg/5ml oral solution (pharmacia ltd)
1642 budesonide 400micrograms/dose dry powder inhaler
1676 flixotide 125microgram/actuation inhalation powder (allen & hanburys ltd)
1680 pulmicort ls 50micrograms/dose inhaler (astrazeneca uk ltd)
1698 salbutamol 100micrograms/dose breath actuated inhaler
1711 salbutamol 5mg/2.5ml nebuliser liquid unit dose vials
1725 beclazone 50 easi-breathe inhaler (teva uk ltd)
1727 becotide easi-breathe 50microgram/actuation pressurised inhalation (allen & hanburys ltd)
1734 beclometasone 100micrograms/dose breath actuated inhaler
1741 salbutamol 100micrograms/dose breath actuated inhaler cfc free
1833 theophylline 200mg modified-release tablets
1834 theophylline 400mg modified-release tablets
1861 aerobec 100 autohaler (meda pharmaceuticals ltd)
1882 ventodisks 200microgram/blister disc (allen & hanburys ltd)
1885 beclazone 200 inhaler (teva uk ltd)
1950 ventodisks 400microgram/blister disc (allen & hanburys ltd)
1951 becodisks 400microgram disc (allen & hanburys ltd)
1952 ventolin 400microgram rotacaps (glaxosmithkline uk ltd)

For peer review only

1956 pulmicort 1mg respules (astrazeneca uk ltd)
1957 ventolin 5mg nebules (glaxosmithkline uk ltd)
1959 pulmicort 0.5mg respules (astrazeneca uk ltd)
1972 oxygen bp size af lightweight gas 1360 litres
1974 oxis 12 turbohaler (astrazeneca uk ltd)
1975 oxis 6 turbohaler (astrazeneca uk ltd)
2044 prednisone 2.5 mg tab
2092 budesonide 200micrograms/dose dry powder inhaler
2124 pulmicort refil 200 mcg inh
2125 pulmicort 200microgram refill canister (astrazeneca uk ltd)
2147 theophylline 250mg modified-release capsules
2148 beclometasone 400microgram disc
2149 steri-neb salamol 2.5 mg inh
2152 ipratropium bromide with salbutamol 20mcg + 100mcg
2159 aerobec 50 autohaler (meda pharmaceuticals ltd)
2160 beclometasone 50micrograms/dose breath actuated inhaler
2224 serevent 50micrograms/dose accuhaler (glaxosmithkline uk ltd)
2229 becodisks 100microgram disc (allen & hanburys ltd)
2282 fluticasone 500micrograms/dose dry powder inhaler
2335 qvar 100 inhaler (teva uk ltd)
2368 prednisolone 2.5mg tablet
2390 prednisolone e/c 1 mg tab
2395 salbutamol 2 mg/5ml syr
2437 oxitropium bromide 100micrograms/dose inhaler
2440 flixotide accuhaler 500 500microgram/inhalation inhalation powder (allen & hanburys ltd)
2510 oxygen bp gas 1280 litres
2600 beclometasone 250micrograms/dose breath actuated inhaler
2655 airomir 100micrograms/dose inhaler (teva uk ltd)
2723 fluticasone 25micrograms/dose inhaler
2757 slo-phyllin 250mg capsule (lipha pharmaceuticals ltd)
2758 bricanyl refill canister (astrazeneca uk ltd)
2799 prednisolone 10 mg tab
2850 salbutamol 400microgram inhalation powder capsules
2851 ventolin 200microgram rotacaps (glaxosmithkline uk ltd)
2869 salbutamol 8mg modified-release tablets
2892 becloforte 400microgram disks (glaxosmithkline uk ltd)
2893 beclometasone 200micrograms disc
2949 prednisone 5mg tablets
2951 fluticasone 250microgram/actuation pressurised inhalation
2978 salbutamol 200micrograms/dose dry powder inhaler
2992 beclazone 50 inhaler (teva uk ltd)
2995 nuelin sa 175mg tablets (meda pharmaceuticals ltd)
3018 beclometasone 50micrograms/dose inhaler
3059 prednisolone 50 mg tab
3065 bextasol inhalation powder (allen & hanburys ltd)
3075 becotide 400microgram rotacaps (glaxosmithkline uk ltd)
3119 becloforte integra 250microgram/actuation inhaler with compact spacer (glaxo laboratories ltd)
3150 beclometasone 100micrograms/actuation extrafine particle cfc free inhaler
3163 salbutamol 200micrograms disc
3188 pulmicort complete 50 mcg inh
3189 salbuvent inh inh
3220 qvar 50 autohaler (teva uk ltd)
3254 salbulin 4mg tablet (3m health care ltd)

For peer review only

3289	flixotide 25micrograms/dose inhaler (glaxosmithkline uk ltd)
3297	salmeterol 50micrograms disc
3322	aerolin inh 400 100 mcg aer
3345	sintisone tablet (pharmacia ltd)
3347	oxygen bp size f gas 1360 litres
3363	becloforte 400microgram disks with diskhaler (glaxosmithkline uk ltd)
3388	theophylline 175mg modified-release tablets
3442	pulmicort complete 200 mcg inh
3443	salbutamol 100microgram/inhalation spacehaler (celltech pharma europe ltd)
3534	bricanyl 5mg tablets (astrazeneca uk ltd)
3546	qvar 50 inhaler (teva uk ltd)
3556	beclometasone 50micrograms with salbutamol 100micrograms/inhalation inhaler
3557	prednisone 1mg tablets
3570	budesonide 200micrograms/actuation refill canister
3584	bricanyl 1.5mg/5ml syrup (astrazeneca uk ltd)
3666	seretide 500 accuhaler (glaxosmithkline uk ltd)
3743	filair 50 inhaler (meda pharmaceuticals ltd)
3753	flixotide diskhaler-community pack 250 mcg
3758	pulmadil inhalation powder (3m health care ltd)
3763	terbutaline respules inh
3764	bricanyl respules (5mg/2ml) 2.5 mg/ml inh
3786	fenoterol 100micrograms/dose / ipratropium 40micrograms/dose inhaler
3838	salbutamol 400mcg/beclometh.100mcg r/cap inh
3927	filair 100 inhaler (meda pharmaceuticals ltd)
3947	becotide 100microgram rotacaps (glaxosmithkline uk ltd)
3988	flixotide diskhaler-community pack 100 mcg
3989	flixotide 100microgram disc (allen & hanburys ltd)
3993	filair forte 250micrograms/dose inhaler (meda pharmaceuticals ltd)
3994	salbutamol 4mg modified-release tablets
4055	salbulin 2mg/5ml oral solution (3m health care ltd)
4131	fluticasone 100microgram disc
4132	fluticasone 125microgram/actuation pressurised inhalation
4165	zithromax 250mg capsules (pfizer ltd)
4171	ventolin 2mg tablet (allen & hanburys ltd)
4222	bricanyl 10mg/ml respirator solution (astrazeneca uk ltd)
4268	ipratropium bromide 40micrograms/dose inhaler
4365	beclometasone 100micrograms disc
4412	oxygen bp size g gas 3400 litres
4413	qvar 100 autohaler (teva uk ltd)
4497	ventolin accuhaler 200 200microgram/actuation inhalation powder (glaxo wellcome uk ltd)
4499	aerobec 250microgram/actuation pressurised inhalation (meda pharmaceuticals ltd)
4514	aminophylline 350mg modified-release tablets
4538	oxygen bp size pd gas 300 litres
4541	bricanyl sa 7.5mg tablets (astrazeneca uk ltd)
4545	pulmicort ls 50microgram refill canister (astrazeneca uk ltd)
4593	theophylline 125mg tablets
4601	asmabec 100 clickhaler (focus pharmaceuticals ltd)
4634	salamol 2.5mg/2.5ml nebuliser liquid steri-neb unit dose vials (teva uk ltd)
4640	bricanyl 5mg/2ml nebuliser liquid (astrazeneca uk ltd)
4665	salbulin 100micrograms/dose inhaler (3m health care ltd)
4688	fluticasone 50microgram/actuation pressurised inhalation
4759	beclometasone 100microgram inhalation powder capsules
4791	oxygen bp size c gas 170 litres
4801	budesonide 500micrograms/2ml nebuliser liquid unit dose vials

For peer review only

4803 beclazone 250microgram/actuation inhalation powder (actavis uk ltd)
4842 fenoterol 100microgram/actuation inhaler
4908 ventolin rotahaler (glaxosmithkline uk ltd)
4926 flixotide accuhaler 100 100microgram/inhalation inhalation powder (allen & hanburys ltd)
4942 budesonide 1mg/2ml nebuliser liquid unit dose vials
5057 azithromycin 200mg/5ml oral suspension
5116 azithromycin 250mg capsules
5143 seretide 50 evohaler (glaxosmithkline uk ltd)
5161 seretide 125 evohaler (glaxosmithkline uk ltd)
5170 salamol 100micrograms/dose inhaler cfc free (teva uk ltd)
5172 seretide 250 evohaler (glaxosmithkline uk ltd)
5185 fenoterol 200micrograms/dose inhaler
5223 fluticasone 50micrograms/dose inhaler cfc free
5261 nuelin sa 250 tablets (meda pharmaceuticals ltd)
5308 terbutaline 5mg/2ml nebuliser liquid unit dose vials
5309 flixotide 50micrograms/dose evohaler (glaxosmithkline uk ltd)
5335 zithromax 500mg tablets (pfizer ltd)
5453 uniphyllin continus 400mg tablets (napp pharmaceuticals ltd)
5516 salamol 100micrograms/dose easi-breathe inhaler (teva uk ltd)
5521 beclometasone 200micrograms/dose dry powder inhaler
5522 beclometasone 100micrograms/dose dry powder inhaler
5551 flixotide 0.5mg/2ml nebules (glaxosmithkline uk ltd)
5558 salmeterol 50micrograms with fluticasone 500micrograms cfc free inhaler
5580 flixotide accuhaler 50 50microgram/inhalation inhalation powder (allen & hanburys ltd)
5584 oxygen cylinders size pd (boc ltd)
5683 flixotide 250micrograms/dose evohaler (glaxosmithkline uk ltd)
5718 flixotide 125micrograms/dose evohaler (glaxosmithkline uk ltd)
5740 airomir 100micrograms/dose autohaler (teva uk ltd)
5753 salbutamol 400micrograms disc
5773 oxygen bp size dd gas 460 litres
5780 oxygen cylinders size af (boc ltd)
5804 beclometasone 250micrograms/dose dry powder inhaler
5822 fluticasone 250micrograms/dose inhaler cfc free
5837 salamol steri-neb 5mg/2.5ml nebuliser liquid (numark management ltd)
5864 salmeterol 25micrograms with fluticasone 250micrograms cfc free inhaler
5885 fluticasone 100micrograms/dose dry powder inhaler
5889 salamol 100microgram/inhalation inhalation powder (kent pharmaceuticals ltd)
5898 salamol steri-neb 2.5mg/2.5ml nebuliser liquid (numark management ltd)
5941 uniphyllin continus 300mg tablets (napp pharmaceuticals ltd)
5942 salmeterol 50micrograms with fluticasone 250micrograms cfc free inhaler
5975 fluticasone 125micrograms/dose inhaler cfc free
5976 oxygen bp size dd 460 litre inhalation gas (boc ltd)
5992 beclometasone 50micrograms/dose dry powder inhaler
6081 ipratropium bromide 20micrograms/dose breath actuated inhaler
6276 carbocisteine 250mg/5ml oral solution
6315 slo-phyllin 250mg capsules (merck serono ltd)
6325 symbicort 200/6 turbohaler (astrazeneca uk ltd)
6419 oxygen cylinders size f (boc ltd)
6420 oxygen bp size d gas 340 litres
6423 oxygen bp with integral headset 1360 litre inhalation gas (boc ltd)
6462 salbutamol 95micrograms/dose dry powder inhaler
6522 ipratropium bromide 20micrograms/dose inhaler cfc free
6526 formoterol 12microgram inhalation powder capsules with device

For peer review only

6556 oxygen cylinders size c (boc ltd)
6569 salmeterol 25micrograms with fluticasone 125micrograms cfc free inhaler
6616 salmeterol 25micrograms with fluticasone 50micrograms cfc free inhaler
6619 oxygen bp size cd gas 460 litres
6719 ipratropium bromide 500micrograms/2ml nebuliser liquid unit dose vials
6746 budesonide 400micrograms/dose / formoterol 12micrograms/dose dry powder inhaler
6758 ipratropium 250micrograms/1ml nebuliser liquid steri-neb unit dose vials (teva uk ltd)
6768 oxygen bp size cd 460 litre inhalation gas (boc ltd)
6772 ipratropium bromide 250micrograms/1ml nebuliser liquid unit dose vials
6780 symbicort 400/12 turbohaler (astrazeneca uk ltd)
6796 budesonide 200micrograms/dose / formoterol 6micrograms/dose dry powder inhaler
6802 mucodyne 375mg capsules (sanofi)
6848 oxygen cylinders size dd with integral headset 2 and 4litres/minute flow rate (boc ltd)
6920 mecysteine 100mg gastro-resistant tablets
6938 salmeterol 50micrograms with fluticasone 100micrograms dry powder inhaler
6976 oxygen bp with integral headset gas 430 litres
6988 aminophylline hydrate 100mg modified-release tablets
7013 symbicort 100/6 turbohaler (astrazeneca uk ltd)
7017 salbutamol 100micrograms/dose dry powder inhaler
7031 oxygen bp size e gas 680 litres
7042 oxygen cylinders size e (boc ltd)
7133 formoterol 12micrograms/dose dry powder inhaler
7192 bambuterol 10mg tablets
7239 oxygen cylinders size b10s (air products plc)
7268 serevent 25micrograms/dose evohaler (glaxosmithkline uk ltd)
7270 salmeterol 25micrograms/dose inhaler cfc free
7452 ventolin .25 mg inj
7584 prednisolone 4 mg tab
7602 fluticasone 50microgram disc
7638 fluticasone 250microgram disc
7653 beclometasone 400microgram inhalation powder capsules
7710 prednisolone 15 mg tab
7711 terbutaline 250micrograms/dose inhaler with spacer
7724 betamethasone valerate 100micrograms/actuation inhaler
7730 theo-dur 300mg tablets (astrazeneca uk ltd)
7731 theo-dur 200mg tablets (astrazeneca uk ltd)
7732 theophylline 300mg modified-release tablets
7733 theophylline 250mg modified-release tablets
7788 budesonide 100micrograms/dose dry powder inhaler
7832 choline theophyllinate 200mg tablets
7841 nuelin 125mg tablets (3m health care ltd)
7891 fluticasone 500microgram disc
7934 prednisone 30 mg tab
7935 maxivent 100microgram/inhalation inhalation powder (ashbourne pharmaceuticals ltd)
7948 fluticasone 250micrograms/dose dry powder inhaler
7953 terbutaline 1.5mg/5ml oral solution sugar free
7954 bricanyl 250micrograms/dose spacer inhaler (astrazeneca uk ltd)
7964 beclometasone 50micrograms/ml nebuliser suspension
7965 salbutamol 5mg/ml nebuliser liquid
8012 exirel 15mg capsule (3m health care ltd)
8056 aminophylline 100mg tablets
8057 aminophylline 100mg modified-release tablets
8111 becloforte vm 250microgram/actuation vm pack (allen & hanburys ltd)
8183 oxygen bp gas 640 litres

For peer review only

8251	pulmicort refil 50 mg inh
8252	pirbuterol 15mg capsule
8267	sodium cromoglicate 1mg/dose / salbutamol 100micrograms/dose inhaler
8333	ipratropium bromide 40microgram inhalation powder capsules
8339	fenoterol hydrobromide complete unit inh
8429	ventolin i/v 5 mg inj
8433	budesonide 100micrograms/actuation inhaler
8450	flixotide diskhaler-community pack 50 mcg
8504	exirel 15 mg tab
8505	oxygen bp gas 6400 litres
8522	terbutaline 7.5mg modified-release tablets
8572	rimiterol inhaler
8635	flixotide 50microgram disc (allen & hanburys ltd)
8636	ventolin s/r 8 mg spa
8676	terbutaline 10mg/ml nebuliser liquid
8757	oxygen bp gas 3200 litres
8806	phyllocontin continus 350mg tablet (napp pharmaceuticals ltd)
8955	theophylline 100 mg tab
9018	mucodyne 375mg capsule (aventis pharma)
9092	theophylline 350mg modified release tablets
9164	fluticasone 50micrograms/dose dry powder inhaler
9233	beclometasone 200microgram inhalation powder capsules
9270	ipratropium bromide with fenoterol hydrobromide 500micrograms + 1.25mg/4ml
9384	salbutamol 4mg modified-release capsules
9477	asmabec 100microgram/actuation spacehaler (celltech pharma europe ltd)
9571	beclometasone 250micrograms/actuation vortex inhaler
9577	asmabec 50 clickhaler (focus pharmaceuticals ltd)
9599	beclazone 50microgram/actuation inhalation powder (actavis uk ltd)
9642	mucodyne 250mg/5ml oral solution (aventis pharma)
9651	asmasal 100microgram/inhalation spacehaler (celltech pharma europe ltd)
9658	oxitropium bromide 100micrograms/dose breath actuated inhaler
9711	formoterol 6micrograms/dose dry powder inhaler
9727	prednisolone 50mg tablets
9805	salbutamol 5mg/50ml solution for infusion vials
9889	oxygen cylinders size g (boc ltd)
9895	oxygen bp size df with integral headset gas 1360 litres
9906	mucodyne 250mg/5ml syrup (sanofi)
9921	beclometasone 100micrograms/dose breath actuated inhaler cfc free
9943	visclair 100mg gastro-resistant tablets (ranbaxy (uk) ltd)
10043	oxygen composite cylinders size b10c with integral headset (air products plc)
10053	oxygen connection cubing 1.8m
10090	beclometasone 50micrograms/actuation extrafine particle cfc free inhaler
10218	budesonide 100micrograms/dose / formoterol 6micrograms/dose dry powder inhaler
10321	budesonide 400microgram inhalation powder capsules
10331	nuelin 60mg/5ml liquid (3m health care ltd)
10353	salbuvent rondo
10360	aerocrom inhaler (castlemead healthcare ltd)
10407	phyllocontin paediatric continus 100mg tablets (napp pharmaceuticals ltd)
10433	theophylline 60mg/5ml oral solution
10458	ventolin cr 4mg tablet (allen & hanburys ltd)
10561	aminophylline 250mg/ml injection
10723	theophylline 125mg/5ml syrup
10744	theophylline 80 mg eli
10808	mucodyne paediatric 125mg/5ml syrup (sanofi)
10825	terbutaline 5mg tablets

For peer review only - http://bmjopen.bmj.com/site/about/guidelines.xhtml

10831 biophylline 125mg/5ml oral solution (lorex synthelabo ltd)
10858 pulmadil auto inhalation powder (3m health care ltd)
10958 salbutamol .25 mg inj
10968 foradil 12microgram inhalation powder capsules with device (novartis pharmaceuticals uk ltd)
11046 ipratropium bromide with salbutamol 500micrograms + 2.5mg/2.5ml
11149 betnelan 500microgram tablets (focus pharmaceuticals ltd)
11198 beclometasons 50 micrograms/actuation vortex inhaler
11307 salbutamol 100micrograms/dose / beclometasone 50micrograms/dose inhaler
11410 fluticasone 500micrograms/dose / salmeterol 50micrograms/dose dry powder inhaler
11478 fluticasone 2mg/2ml nebuliser liquid unit dose vials
11497 beclometasone 400micrograms/dose dry powder inhaler
11588 fluticasone 125micrograms/dose / salmeterol 25micrograms/dose inhaler cfc free
11618 fluticasone 250micrograms/dose / salmeterol 25micrograms/dose inhaler cfc free
11659 oxygen bp size dd with integral headset gas 460 litres
11719 slo-phyllin 60mg capsules (merck serono ltd)
11732 beclometasone 50micrograms/dose breath actuated inhaler cfc free
11779 ipratropium bromide 40microgram inhalation powder capsules with device
11993 pro-vent 300mg capsule (wellcome medical division)
12042 ventolin cr 8mg tablet (allen & hanburys ltd)
12144 bambuterol 20mg tablets
12240 theophylline 300mg modified release capsules
12463 pirbuterol 15 mg tab
12479 aerolin inh auto refil 100 mcg aer
12486 bronchodil 500microgram/dose inhalation powder (viatris pharmaceuticals ltd)
12529 fabrol 200mg granules (novartis consumer health uk ltd)
12563 exirel inhalation powder (3m health care ltd)
12699 pecram 225mg modified-release tablet (novartis consumer health uk ltd)
12808 fenoterol 100micrograms/dose / ipratropium bromide 40micrograms/dose breath actuated inhaler
12822 salbutamol 2.5mg with ipratropium bromide 500micrograms/2.5ml unit dose nebuilser solution
12909 salbutamol 100micrograms/dose / ipratropium 20micrograms/dose inhaler
12994 fluticasone 50micrograms/dose / salmeterol 25micrograms/dose inhaler cfc free
13037 pulvinal beclometasone dipropionate 200micrograms/dose dry powder inhaler (chiesi ltd)
13038 pulvinal salbutamol 200micrograms/dose dry powder inhaler (chiesi ltd)
13040 fluticasone 250micrograms/dose / salmeterol 50micrograms/dose dry powder inhaler
13181 easyhaler salbutamol sulphate 100micrograms/dose dry powder inhaler (orion pharma (uk) ltd)
13206 oxygen bp with integral headset gas 300 litres
13273 fluticasone 100micrograms/dose / salmeterol 50micrograms/dose dry powder inhaler
13290 clenil modulite 100micrograms/dose inhaler (chiesi ltd)
13307 bricanyl 500microgram/ml injection (astrazeneca uk ltd)
13522 prednisolone 2 mg tab
13529 amnivent-225 sr tablets (ashbourne pharmaceuticals ltd)
13575 bambec 20mg tablets (astrazeneca uk ltd)
13615 prednisone 10 mg tab
13757 tropiovent steripoule 250microgram/ml nebuliser liquid (ashbourne pharmaceuticals ltd)
13815 beclazone 100microgram/actuation inhalation powder (actavis uk ltd)
13996 salamol 100microgram/inhalation inhalation powder (sandoz ltd)
14294 qvar 50micrograms/dose easi-breathe inhaler (teva uk ltd)
14306 formoterol 12micrograms/dose inhaler cfc free
14321 beclometasone 200micrograms/dose inhaler cfc free

For peer review only

14482 bricanyl 2.5 mg inj
14483 terbutaline 500micrograms/ml injection
14514 zithromax 200mg/5ml oral suspension (pfizer ltd)
14524 bdp 250microgram/actuation spacehaler (celltech pharma europe ltd)
14525 salbutamol 100micrograms/inhalation vortex inhaler
14527 bambec 10mg tablets (astrazeneca uk ltd)
14561 salbutamol 400microgram / beclometasone 200microgram inhalation powder capsules
14567 asmabec 250 clickhaler (focus pharmaceuticals ltd)
14590 asmabec 250microgram/actuation spacehaler (celltech pharma europe ltd)
14700 budesonide 400micrograms/actuation inhaler
14736 pulvinal beclometasone dipropionate 400micrograms/dose dry powder inhaler (chiesi ltd)
14739 norphyllin sr 225mg tablets (teva uk ltd)
14757 pulvinal beclometasone dipropionate 100micrograms/dose dry powder inhaler (chiesi ltd)
14991 aminophylline 250mg/10ml injection
15075 bronchodil 20mg tablet (viatris pharmaceuticals ltd)
15165 reproterol 500micrograms/dose inhaler
15214 oxygen bp size pd 300 litre inhalation gas (medigas ltd)
15281 oxygen cylinders size pa2 with integral headset (air products plc)
15284 slo-phyllin 125mg capsules (merck serono ltd)
15301 carbocisteine 125mg/5ml oral solution
15326 beclometasone 100micrograms/dose inhaler cfc free
15365 theophylline 10mg/5ml sf elixir
15409 theophylline 3 mg sol
15441 fenoterol hydrobromide .5 % sol
15613 salbutamol 500micrograms/1ml solution for injection ampoules
15706 beclometasone 100 micrograms/actuation vortex inhaler
15979 oxygen bp with integral headset 300 litre inhalation gas (boc ltd)
16054 budesonide 200micrograms/actuation breath actuated powder inhaler
16124 oxygen cylinders (medigas ltd)
16148 clenil modulite 250micrograms/dose inhaler (chiesi ltd)
16151 clenil modulite 200micrograms/dose inhaler (chiesi ltd)
16158 clenil modulite 50micrograms/dose inhaler (chiesi ltd)
16236 pirbuterol acetate inhaler
16305 flixotide 2mg/2ml nebules (glaxosmithkline uk ltd)
16523 oxygen cylinders size cd with integral headset 0-15litres/minute flow rate (boc ltd)
16577 easyhaler salbutamol sulphate 200micrograms/dose dry powder inhaler (orion pharma (uk) ltd)
16584 beclometasone 50micrograms/dose inhaler cfc free
16625 ventide rotacaps (glaxosmithkline uk ltd)
16724 prednisone 50 mg tab
16994 aminophylline hydrate 350mg modified-release tablets
17002 aminophylline hydrate 225mg modified-release tablets
17005 oxygen bp size cd with integral headset gas 460 litres
17140 aminophylline 200mg tablets
17185 ventolin 500micrograms/1ml solution for injection ampoules (glaxosmithkline uk ltd)
17465 fluticasone 500micrograms/2ml nebuliser liquid unit dose vials
17644 oxygen bp with integral headset gas 2122 litres
17654 easyhaler beclometasone 200micrograms/dose dry powder inhaler (orion pharma (uk) ltd)
17670 easyhaler budesonide 100micrograms/dose dry powder inhaler (orion pharma (uk) ltd)
17696 ventmax sr 4mg capsules (chiesi ltd)

For peer review only

17874 monovent 1.5mg/5ml oral solution (lagap)
17901 bricanyl nebule 2.5 ml
18140 respontin 500micrograms/2ml nebules (glaxosmithkline uk ltd)
18288 choline theophyllinate 100mg tablets
18299 fenoterol 1.25mg/4ml / ipratropium 500micrograms/4ml nebuliser liquid unit dose vials
18314 aerocrom syncroner with spacer (castlemead healthcare ltd)
18394 bdp 50microgram/actuation spacehaler (celltech pharma europe ltd)
18421 respontin nebules 250microgram/ml nebuliser liquid (glaxo wellcome uk ltd)
18456 salbutamol 200microgram / beclometasone 100microgram inhalation powder capsules
18484 ventide paediatric rotacaps (glaxosmithkline uk ltd)
18537 budesonide 200microgram inhalation powder capsules
18622 salbulin 2mg tablet (3m health care ltd)
18848 qvar 100micrograms/dose easi-breathe inhaler (teva uk ltd)
18937 sabidal sr 270 270 mg tab
18968 salbutamol 5mg/5ml solution for infusion ampoules
18988 choline theophyllinate 62.5mg/5ml oral solution
19031 bdp 100microgram/actuation spacehaler (celltech pharma europe ltd)
19121 beclometasone 100micrograms with salbutamol 200micrograms inhalation capsules
19376 beclometasone 200micrograms with salbutamol 400micrograms inhalation capsules
19389 asmabec 50microgram/actuation spacehaler (celltech pharma europe ltd)
19401 beclometasone 250micrograms/actuation inhaler and compact spacer
19563 becotide for nebuliser
19642 ventolin nebules
19649 ventolin rotahaler
19653 ventolin respirator
19726 ventolin s/r
19732 cobutolin inh
19735 uniphyllin continus
19736 becotide susp for nebulisation
19737 atrovent nebuliser solution (1ml vial)
19799 tulobuterol 2mg
19805 atrovent
20095 precortisyl forte 25mg tablet (aventis pharma)
20225 aminophylline 500 mg inj
20670 prednisolone e/c
20675 salbutamol rotahaler complete unit
20680 aerolin autohaler
20707 becotide 100
20720 atrovent forte
20763 becloforte
20781 salbutamol u.dose nebulising 2.5mg/2.5ml
20803 ipratropium bromide nebuliser solution
20812 pulmicort refill
20825 spacehaler bdp 250microgram/actuation spacehaler (celltech pharma europe ltd)
20838 salbuvent 2mg tablet (pharmacia ltd)
21005 beclometasone 250micrograms/dose inhaler cfc free
21102 salbutamol 2mg/5ml oral solution (lagap)
21330 oxygen composite cylinders size if2 with integral headset (medigas ltd)
21331 oxygen composite cylinders with integral headset (air products plc)
21402 oxygen cylinders size pd (air products plc)
21482 beclometasone 100micrograms/dose inhaler (generics (uk) ltd)
21769 lasma 300mg tablet (pharmax ltd)
21833 decortisyl 5mg tablet (roussel laboratories ltd)

For peer review only

21859 asmaven 100microgram inhalation powder (berk pharmaceuticals ltd)
22080 aminophylline 20 ml inj
22225 beclomethasone /salbutamol
22313 ventmax sr 8mg capsules (chiesi ltd)
22430 spacehaler salbutamol 100microgram/inhalation spacehaler (celltech pharma europe ltd)
22467 salbutamol respirator soln
22512 salbutamol inhaler
22550 duovent
22661 pirbuterol 10mg capsule
22663 respacal 2mg tablet (ucb pharma ltd)
22669 choline theophyllinate 270 mg tab
22790 reproterol 10mg/ml respirator solution
22828 carbocisteine 750mg/5ml forte oral solution
23269 maxivent 2.5mg/2.5ml nebuliser liquid unit dose steripoule vials (ashbourne pharmaceuticals ltd)
23337 oxygen cylinder f size 1280 litres
23512 precortisyl 5mg tablet (hoechst marion roussel)
23567 respontin 250micrograms/1ml nebules (glaxosmithkline uk ltd)
23572 aminophylline sr 225mg modified-release tablet (ivax pharmaceuticals uk ltd)
23675 pulmicort l.s. refil
23688 ventolin rotacaps
23709 ipratropium 500micrograms/2ml nebuliser liquid steri-neb unit dose vials (teva uk ltd)
23741 novolizer budesonide 200microgram/actuation pressurised inhalation (meda pharmaceuticals ltd)
23787 exirel 10mg capsule (3m health care ltd)
23905 oxygen cylinders size d (boc ltd)
23961 ipratropium bromide 250microgram/ml inhalation vapour (galen ltd)
24219 becotide rotacaps
24380 sodium cromoglicate 1mg/dose / salbutamol 100micrograms/dose inhaler with spacer
24418 biophylline 350mg tablet (lorex synthelabo ltd)
24456 carbocisteine 375mg tablets
24645 ventolin 5mg/5ml solution for infusion ampoules (glaxosmithkline uk ltd)
24660 betamethasone valerate
24674 biophylline 500mg tablet (lorex synthelabo ltd)
24716 prednisolone e/c
24898 spacehaler bdp 100microgram/actuation spacehaler (celltech pharma europe ltd)
25020 ipratropium bromide (forte)
25073 salbutamol
25093 theophylline s/r
25204 beclometasone 100micrograms/dose inhaler (a a h pharmaceuticals ltd)
25218 salbutamol cfc/free b/a
25272 precortisyl 1mg tablet (hoechst marion roussel)
25339 maxivent 5mg/2.5ml nebuliser liquid unit dose steripoule vials (ashbourne pharmaceuticals ltd)
25784 atimos modulite 12micrograms/dose inhaler (chiesi ltd)
25820 bronchodil 10mg/5ml oral solution (viatris pharmaceuticals ltd)
25821 exirel 7.5mg/5ml oral solution (3m health care ltd)
25829 pirbuterol 7.5mg/5ml oral solution
26063 beclometasone 100micrograms/dose inhaler (teva uk ltd)
26079 uniphyllin paediatric continus
26420 exirel 10 mg tab
26525 ventolin
26616 ipratropium bromide with fenoterol hydrobromide 0micrograms + 100micrograms/actuation

For peer review only

26665 pulmicort complete
26716 airomir autohaler cfc free b/a
26829 brelomax 2mg tablet (abbott laboratories ltd)
26873 cobutolin 2mg tablet (actavis uk ltd)
27040 phyllocontin continus
27188 easyhaler budesonide 200micrograms/dose dry powder inhaler (orion pharma (uk) ltd)
27301 bromhexine hcl 8mg tablets
27340 salbuvent 0.5mg/ml injection (pharmacia ltd)
27505 ipratropium bromide with fenoterol hydrobromide 40micrograms + 100micrograms/actuation
27525 becotide 50
27558 choledyl
27573 ventolin
27583 pulmicort
27679 beclometasone 100microgram/actuation pressurised inhalation (approved prescription services ltd)
27842 aminophylline 2 ml inj
27889 prednisolone
27915 fluticasone prop disk refill
27959 prednisolone
27962 deltastab 1mg tablet (waymade healthcare plc)
28073 beclometasone 250microgram/actuation pressurised inhalation (approved prescription services ltd)
28241 aminophylline 250mg/10ml solution for injection minijet pre-filled syringes (ucb pharma ltd)
28376 prednisolone 2.5mg gastro-resistant tablet (biorex laboratories ltd)
28508 salbutamol 100microgram/inhalation inhalation powder (ivax pharmaceuticals uk ltd)
28577 ventolin 50microgram/ml injection (allen & hanburys ltd)
28640 beclometasone 100microgram/actuation inhalation powder (actavis uk ltd)
28761 spacehaler bdp 50microgram/actuation spacehaler (celltech pharma europe ltd)
28859 deltastab 5mg tablet (waymade healthcare plc)
28881 salbutamol 2mg/5ml oral solution sugar free (a a h pharmaceuticals ltd)
29267 salbuvent 4mg tablet (pharmacia ltd)
29273 aminophylline 225mg modified-release tablet (hillcross pharmaceuticals ltd)
29325 beclometasone 250micrograms/dose inhaler (generics (uk) ltd)
30118 salbutamol 100micrograms/dose inhaler cfc free (teva uk ltd)
30204 salbutamol 200micrograms inahalation capsules
30210 beclometasone 250micrograms/dose inhaler (teva uk ltd)
30212 salbutamol cyclohaler
30229 ipratropium bromide 250microgram/ml nebuliser liquid (galen ltd)
30230 salbutamol 100micrograms/actuation breath actuated inhaler
30238 beclometasone 50microgram/actuation pressurised inhalation (approved prescription services ltd)
30240 aerolin autohaler 100microgram/actuation pressurised inhalation (3m health care ltd)
30390 deltastab 2 mg tab
30596 aminophylline 225mg modified-release tablet (actavis uk ltd)
30649 easyhaler budesonide 400micrograms/dose dry powder inhaler (orion pharma (uk) ltd)
30971 decortisyl 25 mg tab
31082 salbuvent 5mg/ml respirator solution (pharmacia ltd)
31290 salbulin cfc free
31327 prednisolone steaglate 6.65mg tablet
31758 uniphyllin continus
31774 beclometasone 50micrograms/dose inhaler (generics (uk) ltd)

For peer review only

31845	salapin 2mg/5ml syrup (pinewood healthcare)
31933	salbutamol 100micrograms/dose inhaler (a a h pharmaceuticals ltd)
32050	salbutamol 400 cyclocaps (teva uk ltd)
32102	salbutamol 4mg tablets (a a h pharmaceuticals ltd)
32461	choline theophyllinate 90 mg tab
32812	numotac 10mg tablet (3m health care ltd)
32874	beclometasone 50microgram/actuation inhalation powder (actavis uk ltd)
32893	theophylline 100mg/lysine 74mg mg tab
33089	salbutamol 100micrograms/dose inhaler (kent pharmaceuticals ltd)
33258	beclometasone 250micrograms/dose inhaler (a a h pharmaceuticals ltd)
33373	salbutamol 200 cyclocaps (teva uk ltd)
33588	salbutamol 100micrograms/dose inhaler (generics (uk) ltd)
33691	prednisolone 5mg gastro-resistant tablet (biorex laboratories ltd)
33817	salbutamol 100micrograms/dose inhaler cfc free (actavis uk ltd)
33849	beclometasone 100microgram/actuation inhalation powder (neo laboratories ltd)
33888	azithromycin 250mg tablets
33988	prednisolone 5mg tablet (co-pharma ltd)
33990	prednisolone 5mg tablet (ivax pharmaceuticals uk ltd)
34018	salbutamol 5mg/2.5ml nebuliser liquid (galen ltd)
34029	salbutamol 400micrograms inahalation capsules
34109	prednisolone 5 mg gastro-resistant tablet
34134	aerolin 400 100microgram/actuation inhalation powder (3m health care ltd)
34162	salbutamol 2.5mg/2.5ml nebuliser liquid (galen ltd)
34310	salbutamol 100micrograms/dose inhaler cfc free (a a h pharmaceuticals ltd)
34311	salbutamol 100microgram/inhalation inhalation powder (berk pharmaceuticals ltd)
34315	beclometasone 250microgram/actuation inhalation powder (actavis uk ltd)
34428	beclometasone 50microgram/actuation inhalation powder (neo laboratories ltd)
34618	salbutamol 2mg tablets (actavis uk ltd)
34619	salbutamol 100microgram/inhalation inhalation powder (kent pharmaceuticals ltd)
34631	prednisolone 1mg tablet (co-pharma ltd)
34702	salbutamol 100microgram/inhalation inhalation powder (c p pharmaceuticals ltd)
34739	beclometasone 50micrograms/dose inhaler (teva uk ltd)
34794	beclometasone 200micrograms/dose inhaler (a a h pharmaceuticals ltd)
34859	beclometasone 250microgram/actuation inhalation powder (neo laboratories ltd)
34914	prednisolone 1mg tablet (celltech pharma europe ltd)
34919	beclometasone 50micrograms/dose inhaler (a a h pharmaceuticals ltd)
34938	salbutamol 4mg tablets (actavis uk ltd)
35011	tiotropium bromide 18microgram inhalation powder capsules
35014	tiotropium bromide 18microgram inhalation powder capsules with device
35015	erdosteine 300mg capsules
35071	becodisks 200microgram (glaxosmithkline uk ltd)
35106	becodisks 100microgram with diskhaler (glaxosmithkline uk ltd)
35107	beclometasone 400microgram inhalation powder blisters with device
35113	beclometasone 200microgram inhalation powder blisters
35118	becodisks 400microgram with diskhaler (glaxosmithkline uk ltd)
35165	serevent 50microgram disks with diskhaler (glaxosmithkline uk ltd)
35178	erdotin 300mg capsules (galen ltd)
35225	flixotide 100microgram disks with diskhaler (glaxosmithkline uk ltd)
35288	beclometasone 400microgram inhalation powder blisters
35293	beclometasone 200microgram inhalation powder blisters with device
35299	becodisks 400microgram (glaxosmithkline uk ltd)
35374	flixotide 500microgram disks (glaxosmithkline uk ltd)
35392	flixotide 500microgram disks with diskhaler (glaxosmithkline uk ltd)
35408	becodisks 100microgram (glaxosmithkline uk ltd)
35430	becodisks 200microgram with diskhaler (glaxosmithkline uk ltd)

For peer review only

35461 flixotide 250microgram disks with diskhaler (glaxosmithkline uk ltd)
35503 salmeterol 50microgram inhalation powder blisters
35510 budesonide 200micrograms/dose dry powder inhalation cartridge with device
35522 bricanyl 500micrograms/1ml solution for injection ampoules (astrazeneca uk ltd)
35542 salmeterol 50microgram inhalation powder blisters with device
35557 ipramol nebuliser solution 2.5ml steri-neb unit dose vials (teva uk ltd)
35566 oxygen bp with set gas 1360 litres
35580 beclometasone 100microgram inhalation powder blisters with device
35602 budesonide 200micrograms/dose dry powder inhalation cartridge
35611 flixotide 250microgram disks (glaxosmithkline uk ltd)
35631 budelin novolizer 200micrograms/dose inhalation powder (meda pharmaceuticals ltd)
35638 fluticasone 100microgram inhalation powder blisters with device
35652 beclometasone 100microgram inhalation powder blisters
35700 fluticasone 500microgram inhalation powder blisters with device
35724 budelin novolizer 200micrograms/dose inhalation powder refill (meda pharmaceuticals ltd)
35725 formoterol easyhaler 12micrograms/dose dry powder inhaler (orion pharma (uk) ltd)
35744 bricanyl 2.5mg/5ml solution for injection ampoules (astrazeneca uk ltd)
35772 fluticasone 100microgram inhalation powder blisters
35825 serevent 50microgram disks (glaxosmithkline uk ltd)
35861 terbutaline 2.5mg/5ml solution for injection ampoules
35862 terbutaline 500micrograms/1ml solution for injection ampoules
35905 fluticasone 250microgram inhalation powder blisters
35986 flixotide 50microgram disks (glaxosmithkline uk ltd)
36021 fluticasone 50microgram inhalation powder blisters with device
36090 flixotide 100microgram disks (glaxosmithkline uk ltd)
36290 flixotide 50microgram disks with diskhaler (glaxosmithkline uk ltd)
36401 fluticasone 250microgram inhalation powder blisters with device
36462 fluticasone 500microgram inhalation powder blisters
36677 reproterol 10mg/5ml oral solution
36864 tiotropium bromide 2.5micrograms/dose solution for inhalation cartridge with device cfc free
37432 fostair 100micrograms/dose/6micrograms/dose inhaler (chiesi ltd)
37447 fluticasone 50microgram inhalation powder blisters
37470 beclometasone 100micrograms/dose / formoterol 6micrograms/dose inhaler cfc free
37612 terbutaline 5mg/2ml nebuliser liquid unit dose vials (galen ltd)
37791 ipratropium bromide 250microgram/ml
38079 salbutamol 100micrograms/dose dry powder inhalation cartridge with device
38097 salbutamol cyclocaps 200microgram inhalation powder (dupont pharmaceuticals ltd)
38120 theophylline 500mg modified release tablets
38136 salbulin novolizer 100micrograms/dose inhalation powder (meda pharmaceuticals ltd)
38214 salbutamol 100micrograms/dose dry powder inhalation cartridge
38226 salbulin novolizer 100micrograms/dose inhalation powder refill (meda pharmaceuticals ltd)
38407 prednisolone 20mg tablet
38416 salbutamol cyclocaps 400microgram inhalation powder (dupont pharmaceuticals ltd)
38419 terbutaline 1.5mg/5ml oral solution sugar free (a a h pharmaceuticals ltd)
39040 phyllocontin forte continus 350mg tablets (napp pharmaceuticals ltd)
39099 pulmicort 100micrograms/dose inhaler cfc free (astrazeneca uk ltd)
39102 budesonide 100micrograms/dose inhaler cfc free
39200 aerobec forte 250 autohaler (meda pharmaceuticals ltd)
39879 budesonide 200micrograms/dose inhaler cfc free
40057 pulmicort 200micrograms/dose inhaler cfc free (astrazeneca uk ltd)
40177 ipratropium bromide 250microgram/ml nebuliser liquid (hillcross pharmaceuticals ltd)
40218 azithromycin 500mg tablets (teva uk ltd)

For peer review only

40599 salbutamol 5mg/2.5ml nebuliser liquid unit dose steripoule vials (galen ltd)
40637 ipratropium 250micrograms/1ml nebuliser liquid unit dose steripoule vials (galen ltd)
40655 salbuvent 100microgram/actuation inhalation powder (pharmacia ltd)
40709 salbutamol 2.5mg/2.5ml nebuliser liquid unit dose vials (a a h pharmaceuticals ltd)
40832 ipratropium 500micrograms/2ml nebuliser liquid unit dose steripoule vials (galen ltd)
41269 beclometasone 400 cyclocaps (teva uk ltd)
41412 beclometasone 400micrograms/actuation inhaler
41548 salbutamol 2mg tablets (approved prescription services ltd)
41549 salbutamol 2mg tablet (c p pharmaceuticals ltd)
41691 salbutamol 2mg/5ml oral solution sugar free (sandoz ltd)
41832 monovent 1.5mg/5ml syrup (sandoz ltd)
42103 tulobuterol 1mg/5ml sugar free syrup
42279 salbutamol 2.5mg/2.5ml nebuliser liquid unit dose steripoule vials (galen ltd)
42497 salbutamol 8mg tablet
42511 aminophylline 25mg/ml injection (celltech pharma europe ltd)
42830 ventolin 100micrograms/dose evohaler (glaxosmithkline uk ltd)
42858 ventolin 200micrograms/dose accuhaler (glaxosmithkline uk ltd)
42867 terbutaline 1.5mg/5ml oral solution (sandoz ltd)
42886 bricanyl 500micrograms/dose turbohaler (astrazeneca uk ltd)
42910 aminophylline 250mg/10ml solution for injection ampoules (martindale pharmaceuticals ltd)
42928 flixotide 100micrograms/dose accuhaler (glaxosmithkline uk ltd)
42985 flixotide 50micrograms/dose accuhaler (glaxosmithkline uk ltd)
42994 flixotide 250micrograms/dose accuhaler (glaxosmithkline uk ltd)
43046 salipraneb 0.5mg/2.5mg nebuliser solution 2.5ml ampoules (arrow generics ltd)
43074 flixotide 500micrograms/dose accuhaler (glaxosmithkline uk ltd)
43085 bricanyl 5mg/2ml respules (astrazeneca uk ltd)
43400 clamelle 500mg tablets (actavis uk ltd)
43544 prednisone 5mg tablet (knoll ltd)
43738 indacaterol 150microgram inhalation powder capsules with device
43794 nebusal 7% inhalation solution 4ml ampoules (forest laboratories uk ltd)
43870 sodium chloride 7% inhalation solution 4ml ampoules
43893 onbrez breezhaler 150microgram inhalation powder capsules with device (novartis pharmaceuticals uk ltd)
44064 onbrez breezhaler 300microgram inhalation powder capsules with device (novartis pharmaceuticals uk ltd)
44173 roflumilast 500microgram tablets
44380 prednisone 1mg modified-release tablets
44431 daxas 500microgram tablets (takeda uk ltd)
44713 salbutamol 100microgram/inhalation inhalation powder (celltech pharma europe ltd)
45302 prednisolone 5mg tablet (biorex laboratories ltd)
45610 indacaterol 300microgram inhalation powder capsules with device
45863 salbutamol 5mg/2.5ml nebuliser liquid (generics (uk) ltd)
46157 beclometasone 200 cyclocaps (teva uk ltd)
46551 salbutamol 100microgram/inhalation inhalation powder (neo laboratories ltd)
46695 azithromycin 500mg tablet (hillcross pharmaceuticals ltd)
49016 sodium chloride 7% inhalation solution 4ml vials
49045 nebusal 7% inhalation solution 4ml vials (forest laboratories uk ltd)
49530 azithromycin 200mg/5ml oral suspension (sandoz ltd)
50508 mucodyne 250mg/5ml syrup (sigma pharmaceuticals plc)
52029 zithromax 250mg capsules (mawdsley-brooks & company ltd)
53303 carbocisteine 375mg capsules (arrow generics ltd)

For peer review only

Spirometry codes

medcode	readterm
6118	spirometry
10336	spirometry reversibility
10337	spirometry screening
10420	spirometry reversibility negative
10492	spirometry reversibility positive
13683	referral for spirometry
14453	forced expiratory volume - fev
19428	chronic obstructive pulmonary disease excluded by spirometry
26241	spirometry indicated
29015	spirometry
45993	incentive spirometry
102522	post bronchodilator spirometry
6091	Percent predicted FEV1
8512	FEV1/FVC percent
10320	Forced expired volume in 1 second
11078	FEV1/FVC > 70% of predicted
14455	FEV1/FVC ratio normal
14456	FEV1/FVC ratio
19830	FEV1 after bronchodilation
19832	FEV1/FVC ratio after bronchodilator
23237	FEV1 before bronchodilation
23285	FEV1/FVC ratio abnormal
25083	FEV1/FVC < 70% of predicted
27141	FEV1/FVC ratio before bronchodilator
43040	FEV1 pre steroids
43041	FEV1 post steroids
58632	FEV1/FVC ratio pre steroids
58633	FEV1/FVC ratio post steroids
99777	Forced expired volume in 1 second reversibility
100391	Forced expired volume in 1 second percentage change
101079	Percentage predicted FEV1 after bronchodilation

Non-specific bronchitis codes / Frequent winter bronchitis

medcode	readterm
148	bronchitis unspecified
152	wheezy bronchitis
3243	chronic bronchitis
3480	bronchitis nos
4519	h/o: bronchitis
5798	chronic asthmatic bronchitis
5909	chronic wheezy bronchitis
7092	recurrent wheezy bronchitis
11150	mucopurulent chronic bronchitis
13247	fh: bronchitis
13262	fh: bronchitis/coad
14798	emphysematous bronchitis
15157	chronic bronchitis nos
15626	chronic catarrhal bronchitis
17359	chest infection - unspecified bronchitis
24248	mixed simple and mucopurulent chronic bronchitis
25603	simple chronic bronchitis
27819	obstructive chronic bronchitis

For peer review only

37959	fetid chronic bronchitis
40159	purulent chronic bronchitis
44525	obstructive chronic bronchitis nos
61118	simple chronic bronchitis nos
61513	mucopurulent chronic bronchitis nos
62913	[v]screening for chronic bronchitis or emphysema
66043	other chronic bronchitis
68066	other chronic bronchitis nos
202167	INFLUENZAL BRONCHITIS
207165	Acute streptococcal bronchitis
207166	Acute viral bronchitis unspecified
216109	Acute membranous bronchitis
216111	Acute bronchitis due to mycoplasma pneumoniae
216112	Acute bronchitis NOS
216141	Chest infection - unspecified bronchopneumonia
220234	BRONCHITIS FIBRINOUS ACUTE
225216	Acute pseudomembranous bronchitis
225241	Bronchitis NOS
234340	Acute bronchitis due to rhinovirus
239395	H/O: bronchitis
243349	Acute bronchitis and bronchiolitis
243350	Acute pneumococcal bronchitis
243351	Acute bronchitis due to echovirus
243389	Bronchitis unspecified
261710	Acute wheezy bronchitis
261711	Acute purulent bronchitis
261712	Acute croupous bronchitis
261713	Acute bronchitis due to respiratory syncytial virus
271007	Acute bronchitis due to coxsackievirus
280054	Acute fibrinous bronchitis
280055	Acute bronchitis due to parainfluenza virus
280082	Chest infection - unspecified bronchitis
289164	Acute bronchitis
289165	Acute neisseria catarrhalis bronchitis
289166	Subacute bronchitis unspecified
289233	[X]Acute bronchitis due to other specified organisms
293231	BRONCHITIS SUBACUTE
298435	Acute haemophilus influenzae bronchitis
298436	Acute bacterial bronchitis unspecified
298440	Acute bronchitis or bronchiolitis NOS
303952	BRONCHITIS ACUTE
303954	VIRAL BRONCHITIS
303963	BRONCHOPNEUMONIA
306525	BRONCHITIS PURULENT
306528	CATARRHAL BRONCHITIS
308788	Acute bronchitis

Respiratory symptom codes

Wheeze

medcode	readterm
152	wheezy bronchitis
173	wheezing
2210	[d]wheezing

For peer review only

2891 wheezing symptom
4836 nocturnal cough / wheeze
5861 o/e - expiratory wheeze
5909 chronic wheezy bronchitis
5978 acute wheezy bronchitis
7092 recurrent wheezy bronchitis
42317 increasing exercise wheeze
100954 [d]mild wheeze
101037 [d]moderate wheeze
101073 [d]severe wheeze
101421 [d]very severe wheeze
102480 on examination - inspiratory wheeze

For peer review only

Chronic cough

medcode	readterm
92	cough
292	chesty cough
1025	bronchial cough
1160	[d]cough
1234	productive cough nos
1273	c/o - cough
1612	chronic cough
3068	night cough present
3628	persistent cough
3645	coughing up phlegm
4070	morning cough
4836	nocturnal cough / wheeze
4931	dry cough
7706	productive cough -clear sputum
7707	cough symptom nos
7708	productive cough-yellow sputum
7773	productive cough -green sputum
16717	smokers' cough
22318	difficulty in coughing up sputum
29318	evening cough
43795	unexplained cough
60903	cough aggravates symptom
100333	cough on exercise

Exertional breathlessness

medcode	readterm
735	[d]breathlessness
1429	breathlessness
3092	[d]dyspnoea
5175	breathlessness symptom
5896	dyspnoea - symptom
6326	breathless - moderate exertion
7000	o/e - dyspnoea
7932	breathless - mild exertion
19426	mrc breathlessness scale: grade 3
19427	mrc breathlessness scale: grade 2
19429	mrc breathlessness scale: grade 5

19430 mrc breathlessness scale: grade 4
19432 mrc breathlessness scale: grade 1
21801 breathlessness nos
24889 breathless - strenuous exertion
31143 breathless - at rest
42287 borg breathlessness score: 6 severe (+)
53771 dyspnoea on exertion
57193 borg breathlessness score: 3 moderate
57759 borg breathlessness score: 2 slight
59860 borg breathlessness score: 4 somewhat severe
64049 borg breathlessness score: 5 severe
67566 borg breathlessness score: 9 very, very sev (almost maximal)
68707 borg breathlessness score: 1 very slight
70061 borg breathlessness score: 7 very severe
70818 borg breathlessness score: 0.5 very, very slight
72334 borg breathlessness score: 8 very severe (+)
101843 borg breathlessness score: 10 maximal
741 [D]Shortness of breath
2563 [D]Respiratory distress
2575 Short of breath on exertion
2931 Difficulty breathing
4822 Shortness of breath
5349 Shortness of breath symptom
9297 [D]Respiratory insufficiency
22094 Short of breath dressing/undressing
40813 Unable to complete a sentence in one breath

Sputum codes
medcode readterm
292 chesty cough
1025 bronchial cough
1234 productive cough nos
3645 coughing up phlegm
7706 productive cough -clear sputum
7708 productive cough-yellow sputum
7773 productive cough -green sputum
22318 difficulty in coughing up sputum
1251 [d]abnormal sputum
3727 sputum sent for c/s
8287 sputum sample obtained
8760 [d]positive culture findings in sputum
9807 sputum - symptom
14804 sputum appears infected
15430 [d]sputum abnormal - colour
16026 sputum examination: abnormal
18964 sputum clearance
20086 [d]sputum abnormal - amount
22318 difficulty in coughing up sputum
23582 [d]abnormal sputum nos
24181 sputum: mucopurulent
29294 sputum appears normal
30754 yellow sputum
30904 sputum sent for examination
31417 sputum - not infected
35577 sputum examination - general

For peer review only

36515 [d]abnormal sputum - tenacious
36880 green sputum
40201 sputum microscopy: nad
40202 mucoid sputum - o/e
42573 clear sputum
43270 sputum evidence of infection
43272 sputum examination nos
44214 [d]sputum abnormal - odour
49029 sputum gen. exam. nos
49694 sputum: organism on gram stain
52806 sputum: frothy/watery
54177 sputum: excessive - mucoid
61079 sputum inspection nos
91680 expectoration of induced sputum from respiratory tract
100484 volume of sputum
100524 moderate sputum
100629 white sputum
100647 copious sputum
100931 brown sputum
101782 profuse sputum
103209 grey sputum

QOF codes

H3... Chronic obstructive pulmonary disease
H31.. Chronic bronchitis
H310. Simple chronic bronchitis
H3100 Chronic catarrhal bronchitis
H310z Simple chronic bronchitis NOS
H311. Mucopurulent chronic bronchitis
H3110 Purulent chronic bronchitis
H3111 Fetid chronic bronchitis
H311z Mucopurulent chronic bronchitis NOS
H312. Obstructive chronic bronchitis
H3120 Chronic asthmatic bronchitis
H3121 Emphysematous bronchitis
H3123 Bronchiolitis obliterans
H312z Obstructive chronic bronchitis NOS
H313. Mixed simple and mucopurulent chronic bronchitis
H31y. Other chronic bronchitis
H31y1 Chronic tracheobronchitis
H31yz Other chronic bronchitis NOS
H31z. Chronic bronchitis NOS
H32.. Emphysema
H320. Chronic bullous emphysema
H3200 Segmental bullous emphysema
H3201 Zonal bullous emphysema
H3202 Giant bullous emphysema
H3203 Bullous emphysema with collapse
H320z Chronic bullous emphysema NOS
H321. Panlobular emphysema
H322. Centrilobular emphysema
H32y. Other emphysema
H32y0 Acute vesicular emphysema

For peer review only

H32y1 Atrophic (senile) emphysema
H32y2 MacLeod's unilateral emphysema
H32yz Other emphysema NOS
H32z. Emphysema NOS
H36.. Mild chronic obstructive pulmonary disease
H37.. Moderate chronic obstructive pulmonary disease
H38.. Severe chronic obstructive pulmonary disease
H39.. Very severe chronic obstructive pulmonary disease
H3A.. End stage chronic obstructive airways disease (v23)
H3y.. Other specified chronic obstructive airways disease
~~H3y0. Chronic obstructive pulmonary disease with acute lower respiratory infection~~ (v23)
~~H3y1. Chronic obstructive pulmonary disease with acute exacerbation, unspecified~~ (v23)
H3z.. Chronic obstructive airways disease NOS

GP Questionnaire

1. Do you think this patient has COPD? Yes / No / Uncertain
2. What was the diagnosis of COPD based on? (please circle all that apply)
   Smoking history symptoms spirometry other (please describe)
3. Has a respiratory physician seen the patient and confirmed a diagnosis of COPD?
4. Does the patient have any other respiratory condition?
   If yes, than what?

Table 4: The PPV and proportion of patients diagnosed with COPD who also had cardiovascular disease or asthma

Algorithm	Number with CVDx	Number confirmed COPD	PPV and 95% CI	Number with asthma	Number confirmed COPD	PPV and 95% CI
**COPD Code + spirometry + COPD medication**	20	17	85.0, 59.6-95.6	18	14	72.2, 45.3-89.1
**COPD Code + spirometry**	24	20	83.3, 61.1-94.1	20	14	70.0, 45.0-87.0
**COPD Code + COPD medication**	18	16	88.9, 61.1-97.6	16	9	56.3, 29.8-79.6
**COPD Code only**	18	16	88.9, 61.1-97.6	15	12	80.0, 48.8-94.4
**Bronchitis + COPD medication**	21	8	38.1, 19.1-61.7	27	4	14.8, 5.3-35.1
**Bronchitis only**	19	8	42.1, 21.1-66.5	23	6	26.1, 11.4-49.1
**Symptoms + spirometry**	15	9	60.0, 31.8-82.9	19	4	21.1, 7.3-47.3
**Symptoms only**	9	2	22.2, 3.9-67.0	23	2	8.7, 1.9-31.4

For peer review only

Table 5: The PPV and proportion of patients diagnosed with COPD who were current smokers

Algorithm	Number current smokers	Number confirmed COPD	PPV and 95% CI
**COPD Code + spirometry + COPD medication**	49	44	89.9, 77.1-95.8
**COPD Code + spirometry**	51	39	76.5, 62.5-86.4
**COPD Code + COPD medication**	55	46	83.6, 71.0-91.4
**COPD Code only**	47	41	87.2, 73.8-94.3
**Bronchitis + COPD medication**	67	34	50.7, 38.7-62.7
**Bronchitis only**	64	19	29.7, 19.6-42.3
**Symptoms + spirometry**	50	20	40.0, 27.1-54.5
**Symptoms only**	58	6	10.3, 4.6-21.7

Table 6: The PPV and proportion of patients diagnosed with COPD by COPD severity

Algorithm	GOLD 1 Number confirmed, (PPV and 95% CI)	GOLD 2 Number confirmed, (PPV and 95% CI)	GOLD 3 Number confirmed, (PPV and 95% CI)	GOLD 4 Number confirmed, (PPV and 95% CI)
**COPD Code + spirometry + COPD medication**	9, (69.2, 36.5-89.8)	34, (87.2, 71.8-94.8)	22, (100)	6, (100)
**COPD Code + spirometry**	8, (66.7, 32.9-89.1)	35, (83.3, 68.2-92.1)	20, (100)	3, (75, 4.1-99.5)
**COPD Code + COPD medication**	14, (82.4, 53.7-94.9)	32 (84.2, 68.1-93.0)	20, (95.2, 69.1-99.4)	6, (100)
**COPD Code only**	14, (82.4, 53.7-94.9)	37, (86.0, 71.5-93.8)	14, (93.3, 58.4-99.3)	3, (100)
**Bronchitis + COPD medication**	7, (36.8, 17.3-62.0)	20, (76.9, 55.6-89.9)	11, (91.7, 49.9-99.2)	2, (100)
**Bronchitis only**	3, (20.0, 5.6-51.2)	7, (63.6, 28.8-83.3)	10 (100)	1, (100)
**Symptoms + spirometry**	4, (57.1, 15.0-90.9)	19, (90.5, 66.0-97.9)	10 (90.9, 46.3-99.1)	1, (100)
**Symptoms only**	2, (40, 3.8-91.9)	6, (100)	0	0

For peer review only

Table 7: The PPV and proportion of patients diagnosed with COPD by timing of diagnosis

**Algorithm**	**2004 to end 2008**			**Post 2008**		
	**Number in algorithm**	**Number confirmed COPD**	**PPV and 95% CI**	**Number in algorithm**	**Number confirmed COPD**	**PPV and 95% CI**
**COPD Code + spirometry + COPD medication**	32	28	87.5, 69.8-95.5	53	48	90.6, 78.7-96.1
**COPD Code + spirometry**	14	11	78.6, 46.0-94.0	66	56	84.8, 73.7-91.8
**COPD Code + COPD medication**	27	23	85.2, 64.9-94.7	61	54	88.5, 77.4-94.5
**COPD Code only**	21	16	76.2, 51.7-90.5	68	61	89.7, 79.6-95.1
**Bronchitis + COPD medication**	94	42	44.7, 34.8-55.0	5	2	40.0, 3.8-91.9
**Bronchitis only**	83	22	26.5, 18.0-37.2	5	4	80.0, 11.1-99.2
**Symptoms + spirometry**	63	28	44.4, 32.4-57.1	22	9	40.9, 21.6-63.5
**Symptoms only**	64	10	15.6, 8.5-27.0	26	1	3.8, 0.47-25.4

For peer review only

STROBE Statement—checklist of items that should be included in reports of observational studies

	Item No	Recommendation
**Title and abstract** **Pg 1 and 2**	1	(*a*) Indicate the study's design with a commonly used term in the title or the abstract
		(*b*) Provide in the abstract an informative and balanced summary of what was done and what was found
**Introduction**		
Background/rationale **Pg 3**	2	Explain the scientific background and rationale for the investigation being reported
Objectives **Pg 4**	3	State specific objectives, including any prespecified hypotheses
**Methods**		
Study design **Pg 4**	4	Present key elements of study design early in the paper
Setting **Pg 4**	5	Describe the setting, locations, and relevant dates, including periods of recruitment, exposure, follow-up, and data collection
Participants **Pg 6**	6	(*a*) *Cohort study*—Give the eligibility criteria, and the sources and methods of selection of participants. Describe methods of follow-up<br>*Case-control study*—Give the eligibility criteria, and the sources and methods of case ascertainment and control selection. Give the rationale for the choice of cases and controls<br>*Cross-sectional study*—Give the eligibility criteria, and the sources and methods of selection of participants
		(*b*) *Cohort study*—For matched studies, give matching criteria and number of exposed and unexposed<br>*Case-control study*—For matched studies, give matching criteria and the number of controls per case
Variables **Pg 5,7**	7	Clearly define all outcomes, exposures, predictors, potential confounders, and effect modifiers. Give diagnostic criteria, if applicable
Data sources/ measurement **Pg 7**	8*	For each variable of interest, give sources of data and details of methods of assessment (measurement). Describe comparability of assessment methods if there is more than one group
Bias **Pg 6**	9	Describe any efforts to address potential sources of bias
Study size **Pg 8**	10	Explain how the study size was arrived at
Quantitative variables **Pg 7 and 8**	11	Explain how quantitative variables were handled in the analyses. If applicable, describe which groupings were chosen and why
Statistical methods **Pg 7 and 8**	12	(*a*) Describe all statistical methods, including those used to control for confounding
		(*b*) Describe any methods used to examine subgroups and interactions
		(*c*) Explain how missing data were addressed
		(*d*) *Cohort study*—If applicable, explain how loss to follow-up was addressed<br>*Case-control study*—If applicable, explain how matching of cases and controls was addressed<br>*Cross-sectional study*—If applicable, describe analytical methods taking account of sampling strategy

For peer review only

For peer review only - http://bmjopen.bmj.com/site/about/guidelines.xhtml

(*e*) Describe any sensitivity analyses

Continued on next page

For peer review only

For peer review only - http://bmjopen.bmj.com/site/about/guidelines.xhtml

**Results**		
Participants **Pg 9**	13*	(a) Report numbers of individuals at each stage of study—eg numbers potentially eligible, examined for eligibility, confirmed eligible, included in the study, completing follow-up, and analysed
		(b) Give reasons for non-participation at each stage
		(c) Consider use of a flow diagram
Descriptive data **Pg 9 and 10**	14*	(a) Give characteristics of study participants (eg demographic, clinical, social) and information on exposures and potential confounders
		(b) Indicate number of participants with missing data for each variable of interest
		(c) *Cohort study*—Summarise follow-up time (eg, average and total amount)
Outcome data **Pg 11**	15*	*Cohort study*—Report numbers of outcome events or summary measures over time
		Case-control study—Report numbers in each exposure category, or summary measures of exposure
		Cross-sectional study—Report numbers of outcome events or summary measures
Main results **Pg 10-12**	16	(*a*) Give unadjusted estimates and, if applicable, confounder-adjusted estimates and their precision (eg, 95% confidence interval). Make clear which confounders were adjusted for and why they were included
		(*b*) Report category boundaries when continuous variables were categorized
		(*c*) If relevant, consider translating estimates of relative risk into absolute risk for a meaningful time period
Other analyses **Supplementary material**	17	Report other analyses done—eg analyses of subgroups and interactions, and sensitivity analyses
**Discussion**		
Key results **Pg 12**	18	Summarise key results with reference to study objectives
Limitations **Pg 15**	19	Discuss limitations of the study, taking into account sources of potential bias or imprecision. Discuss both direction and magnitude of any potential bias
Interpretation **Pg 12-15**	20	Give a cautious overall interpretation of results considering objectives, limitations, multiplicity of analyses, results from similar studies, and other relevant evidence
Generalisability **Pg 15**	21	Discuss the generalisability (external validity) of the study results
**Other information**		
Funding **Pg 17**	22	Give the source of funding and the role of the funders for the present study and, if applicable, for the original study on which the present article is based

*Give information separately for cases and controls in case-control studies and, if applicable, for exposed and unexposed groups in cohort and cross-sectional studies.

Note: An Explanation and Elaboration article discusses each checklist item and gives methodological background and published examples of transparent reporting. The STROBE checklist is best used in conjunction with this article (freely available on the Web sites of PLoS Medicine at http://www.plosmedicine.org/, Annals of Internal Medicine at http://www.annals.org/, and Epidemiology at http://www.epidem.com/). Information on the STROBE Initiative is available at www.strobe-statement.org.

For peer review only

For peer review only - http://bmjopen.bmj.com/site/about/guidelines.xhtml

BMJ Open

Validation of Chronic Obstructive Pulmonary Disease (COPD) recording in the Clinical Practice Research Datalink (CPRD-GOLD)

Journal:	*BMJ Open*
Manuscript ID:	bmjopen-2014-005540.R1
Article Type:	Research
Date Submitted by the Author:	29-May-2014

Complete List of Authors:	Quint, Jennifer; London School of Hygeine and Tropical Medicine, Epidemiology<br>M�llerova, Hana; GlaxoSmithKline, Epidemiology Department<br>DiSantostefano, Rachael; GlaxoSmithKline, Worldwide Epidemiology<br>Forbes, Harriet; London school of Hygiene and Tropical Medicine, Epidemiology Department<br>Eaton, Susan; MHRA, Clincal Practice Research Datalink Group<br>Hurst, John; UCL Medical School,, Academic Unit of Respiratory Medicine<br>Davis, Kourtney<br>Smeeth, Liam; London School of Hygiene and Tropical Medicine, Epidemiology and Population Health

**Primary Subject Heading**:	Respiratory medicine
Secondary Subject Heading:	Epidemiology
Keywords:	RESPIRATORY MEDICINE (see Thoracic Medicine), THORACIC MEDICINE, Chronic airways disease < THORACIC MEDICINE, Epidemiology < THORACIC MEDICINE

SCHOLARONE™ Manuscripts

For peer review only

Validation of Chronic Obstructive Pulmonary Disease (COPD) recording in the Clinical Practice Research Datalink (CPRD-GOLD)

Jennifer K Quint[1], Hana Müllerova[2], Rachael L DiSantostefano[3], Harriet Forbes[1], Susan Eaton[4], John R Hurst[5], Kourtney Davis[2], Liam Smeeth[1].

[1]*Department of Non-communicable disease Epidemiology, London School of Hygiene and Tropical Medicine, London, UK*
[2] *Respiratory Epidemiology, GlaxoSmithKline R&D, Uxbridge, UK*
[3] *Respiratory Epidemiology, GlaxoSmithKline R&D, RTP, NC USA*
[4] *Clinical Practice Research Datalink Group, Medicines and Healthcare products Regulatory Agency, London, UK*
[5]*UCL Respiratory Medicine, Royal Free Campus, University College London Medical School, London, UK*

Corresponding author:
Dr. Jennifer K Quint
Department of Non-communicable disease Epidemiology
London School of Hygiene and Tropical Medicine
Keppel Street, London WC1E 7HT
Tel: 0207 927 2622
Email: **Jennifer.quint@lshtm.ac.uk**

Keywords: COPD, diagnosis of COPD, validation, spirometry, electronic health records

Word count: 3448

For peer review only

ABSTRACT

Objectives: The optimal method of identifying people with COPD from electronic primary care records is not known. We assessed the accuracy of different approaches using the Clinical Practice Research Datalink (CPRD), a UK electronic health record database.

Setting: Nine hundred and fifty one participants registered with a CPRD practice in the UK between 1st January 2004 and 31st December 2012. Individuals were selected for ≥1 of eight algorithms to identify people with COPD. General practitioners were sent a brief questionnaire and additional evidence to support a COPD diagnosis was requested. All information received was reviewed independently by two respiratory physicians whose opinion was taken as gold standard.

Primary outcome measure: The primary measure of accuracy was the positive predictive value (PPV); the proportion of people identified by each algorithm for whom COPD was confirmed.

Results: Nine hundred and fifty one questionnaires were sent and 738 (78%) returned. After quality control, 696 (73.2%) patients were included in the final analysis. All four algorithms including a specific COPD diagnostic code performed well. Using a diagnostic code alone the PPV was 86.5% (77.5% to 92.3%) while requiring a diagnosis plus spirometry plus specific medication the PPV was slightly higher at 89.4% (80.7% to 94.5%) but reduced case numbers by 10%. Algorithms without specific diagnostic codes had low PPVs (range 12.2% to 44.4%).

Conclusion: Patients with COPD can be accurately identified from UK primary care records using specific diagnostic codes. Requiring spirometry or COPD medications only marginally improved accuracy. The high accuracy applies since the introduction of an incentivised disease register for COPD as part of QOF (Quality and Outcomes Framework) in 2004.

For peer review only

Article Summary: This paper investigates how individuals with COPD can be accurately identified in electronic health records using specific COPD codes alone without additional information (i.e. presence of spirometry or respiratory medications). This information is essential for appropriate disease management, epidemiological research of COPD and other conditions, where COPD is an important co-morbidity.

Strengths and limitations of this study:

- We have shown that the presence of a specific COPD Read code alone is sufficient to identify patients with COPD from electronic health records. Minimal precision lost by not including spirometry and medications in the algorithm, allows an increase in the number of individuals who can potentially be included in a study by up to 10%.
- We were able to investigate both the accuracy of algorithms when identifying COPD patients within the CPRD, and accuracy of the actual GP diagnosis of COPD.
- The amount of missing data among the responding questionnaires was low, suggesting reasonable data quality.
- Although the overall response rate for this study was acceptable (77.6%), the proportion of questionnaires accompanied with additional evidence allowing for adjudication was lower.

No additional data are available. We would be happy to provide the ISAC protocol to reviewers if required.

INTRODUCTION

For peer review only

Chronic obstructive pulmonary disease (COPD) represents an enormous health burden worldwide. Currently COPD is the 4th leading cause of death and is predicted to become the 3rd by 2020.[1] There are approximately 835,000 people diagnosed with COPD in the UK and an estimated 2,200,000 people remain undiagnosed.[2,3]

Electronic health records in the UK provide an excellent resource in which to study COPD as they offer a large cohort size, the presence of disease severity indicators and long-term follow-up information on a patient's integrated medical history. Although multiple studies have been undertaken to investigate various aspects of COPD over the last 10 years in several electronic health record databases, there is no standard definition used to identify COPD in large databases and codelists used to identify patients with COPD vary by author. Over ten years ago, the diagnosis of COPD was validated in the Clinical Practice Research Datalink (CPRD-GOLD, formerly GPRD) using OXMIS codes.[4] This coding system is now obsolete in CPRD and Read codes are used as standard.

There is no single diagnostic test for COPD. The diagnosis of COPD relies on clinical judgement based on a combination of history, physical examination and confirmation of the presence of airflow obstruction using spirometry.[5] When retrospectively evaluating the accuracy of a COPD diagnosis, commonly used COPD definitions may misclassify patients as having COPD. Using multiple diagnostic codes in combination with pharmacy may improve the accuracy of identification of COPD patients.[6] Further, over the past 10 years the definition of COPD in clinical practice has evolved leading to changes in how COPD is diagnosed and the diagnosis recorded. With the introduction of the Quality and Outcomes Framework (QOF) codes for COPD in 2004 in England and Wales there are more codes available to identify COPD then there were previously. QOF is a voluntary incentive scheme for primary care physicians (GPs) in the UK which contains indicators against which GP practices can score points and hence be rewarded for how well they care for patients. [7] Evaluation of airflow limitation using spirometry is used as standard to confirm COPD

For peer review only

diagnosis and severity of COPD is part of the annual COPD patient review.[3,8] However, even spirometry if incorrectly performed or interpreted can lead to misdiagnosis (both over and under diagnosis of COPD) in approximately 20% of cases. It is also recognised that some subgroups of individuals (e.g. women and individuals under 50 and over 80) are less likely to have spirometry measured.[8]

This study aimed to improve our ability to identify patients with COPD within electronic health records. We used the CPRD, a widely used collection of computerised medical records which is commonly used for clinical and research purposes. CPRD is comparable to other electronic health record databases used in the UK. Our objective was to test the accuracy of different definitions of COPD in the CPRD using Positive Predictive Value (PPV), by comparing the database records with additional information provided by General Practitioners (GPs). This work is important for epidemiological research in COPD and diseases where COPD is an important co-morbidity as well as for clinical practice.

METHODS

Dataset

CPRD is the world's largest validated computerized database of anonymized longitudinal medical records for primary care. [9,10] Data comprise approximately 14 million patients with around 5.4 million of these being currently alive and registered from 660 primary care practices spread throughout the UK. Records are derived from a widely used GP software system and contain complete prescribing and coded diagnostic and clinical information as well as information on tests requested, laboratory results and referrals made at or following on from each consultation. [11]

Codelists and algorithms

Lists of medical codes (Read codes) specific and non-specific for COPD were created prior to the study initiation. Read codes are a hierarchical clinical coding system of over 80,000 terms that are used in general practice in the UK and are entered by the GP into Vision

For peer review only

Software. [12] These data are then uploaded by CPRD after they have been processed and quality checked added to the CPRD database for research use. Specific COPD codes consisted of codes listing either COPD or COPD specific entities of emphysema (see supplementary material for specific codes included). Non-specific codes consisted of a variety of lung diseases that could potentially be COPD e.g. chronic bronchitis. Combinations of codelists and additional material in the form of the presence of spirometry or COPD medications (see supplementary material) were used to make up the eight algorithms. The first four definitions required a specific COPD diagnosis code, with the first three requiring additional documentation (e.g., medication and/or spirometry). The other four definitions required non-specific bronchitis or respiratory symptom codes, with the least specific definition requiring only respiratory symptom codes. Details regarding each algorithm can be found in the supplementary material. Briefly, the eight algorithms were defined as follows, from the expected most specific to most sensitive construct:

1. Specific COPD code and more than one prescription of a COPD medication and presence of spirometry (**COPD Code + spirometry + COPD medication)**
2. Specific COPD code and presence of spirometry (**COPD Code + spirometry)**
3. Specific COPD code and more than one prescription of a COPD medication (**COPD Code + COPD medication)**
4. Specific COPD code only (**COPD Code only)**
5. Non-specific bronchitis code and more than one prescription of a COPD medication (**Bronchitis + COPD medication)**
6. Non-specific bronchitis code only (**Bronchitis only)**
7. Respiratory symptoms and presence of spirometry. Respiratory symptoms consisted of persistent cough, sputum production, or dyspnoea. (**Symptoms + spirometry)**
8. Respiratory symptom definition only (**Symptoms only)**

For peer review only

The presence of spirometry for inclusion in the algorithm was based on an existence of a record of a specific value or a Read code for spirometry rather than examining the actual value. We were able to assess interpretation of spirometry in the subset of patients who had flow volume loops or clinic letters attached and where actual FEV_1 and FVC values were available in CPRD.

Study Population

The study population consisted of a random sample of individuals selected from all subjects registered in CPRD with cohort entry being on or after the 1st January 2004. At cohort entry, people included had to be: over 35 years old, with at least 1 year historical data, and a possible diagnosis of COPD defined as evidence of having ever smoked and a record of a least one specific or possible COPD code or respiratory symptoms suggestive of COPD. The presence of the algorithms was ascertained during a period between patient cohort entry and the 31st December 2012. Patients had to be alive within 4 months of the last collection date of CPRD data for the January 2013 data build for inclusion in the analysis so that CPRD could access their medical records and additional information. For the main analysis, a patient could contribute to one algorithm only. It was possible for an individual to be eligible for more than one algorithm depending upon the codes used in their medical record over the study period. Individuals were randomly selected from the algorithm with the fewest number of participants first and then removed from the cohort so they could not be selected for another algorithm.

CPRD mailed a short, structured questionnaire to GPs in charge of randomly selected patients requesting confirmation of COPD status as well as any available specific information from the individual's medical record including spirometry print outs and hospital respiratory outpatient letters (see supplementary material). Data were "twice encrypted" within CPRD to ensure anonymity; firstly between practices and CPRD and secondly from CPRD to researchers. In the questionnaire the GP was asked whether or not the patient had a

For peer review only

diagnosis of COPD, what that diagnosis was based on, whether or not the patient had seen a respiratory physician and if they had any other respiratory diagnoses. A pilot set of 20 questionnaires were sent to GPs to assess the quality of the questionnaire. In total, 951 questionnaires were sent out assuming an 80% response rate.

Primary outcome

The primary outcome was identification of a diagnosis of COPD according to the pre-defined eight algorithms. The gold standard for the diagnosis of COPD was the decision made after respiratory physicians independently reviewed the evidence from the GP (questionnaire response with or without- additional evidence). Where they did not agree, a 3rd independent physician decided. Additionally, GP diagnosis of COPD was validated in a subset of patients where the GP provided supportive information including spirometry print-outs and hospital letters. This also allowed review of spirometry interpretation in some cases. Although we used two respiratory physicians independently as the gold standard for diagnosing COPD, this was done by reviewing the questionnaire sent to the GP (see appendix) as well as any additional information the GP sent which supported that diagnosis. This supporting information ranged from free text in the GP database to spirometry printouts done in the GP surgery to letters from secondary care. Therefore we were not solely relying on information from secondary care to make the diagnosis unless the GP decided to share that information. In this way we were able to include and validate the diagnosis of COPD in people who were not seen in secondary care.

Analysis

The primary analysis focused on the accuracy of identification of a COPD diagnosis in each of the pre-defined algorithms as defined by positive predicted value (PPV); i.e. the proportion of "true positives" (individuals with COPD) in each algorithm as determined by the gold standard. In addition, within each algorithm, where additional information was provided (lung function, hospital clinic letters), we calculated the accuracy of the GP diagnosis of COPD relative to the gold standard. This allowed review of spirometry interpretation in some cases.

For peer review only

We assessed the impact of commonly occurring co-morbidities on the accuracy of the pre-specified algorithms stratifying for cardiovascular co-morbidity, previous asthma diagnosis, smoking status and, where possible, Global Initiative for Chronic Obstructive Lung Disease (GOLD) staging of airflow limitation severity.[13] Cardiovascular co-morbidity included angina, history of myocardial infarction, previous coronary artery bypass graft / percutaneous coronary intervention or heart failure, but not hypertension due to its lack of overlap of symptoms that could mimic COPD. All covariates for stratification analysis were derived from information available up to cohort entry.

As a post hoc analysis individuals were eligible to be placed into multiple algorithms where possible, and the PPV was calculated for all individuals who had a specific COPD code compared to those with a specific COPD code and additional information (either spirometry or a COPD medication).

Assessment of possible trends in COPD diagnosis recording were also evaluated, including temporal trends in codes used and diagnostic specificity from 2004 to 2011. In addition, we compared our specific COPD codes to those recommended for use by QOF (see supplementary material); [14,15] H31% (excluding H3101 (smokers cough), H31y0 (chronic tracheitis) and H3122 (acute exacerbation of COPD)) H32% H36-H3z (excluding H3y0 and H3y1).

Sample size calculation

Our sample size for each algorithm was chosen to achieve accuracy of the true positives or PPV ±0.08 based on the reviewing physician judgment as the gold standard. Assuming an estimated PPV of 0.85 for any one algorithm, we required a sample of at least 77 individuals in each algorithm to achieve the desired accuracy (95% CI ±0.08). All analyses were performed using STATA 13.

For peer review only

Ethics approval was obtained from ISAC (the Independent Scientific Advisory Committee overseeing CPRD); protocol 12_065 and the LSHTM ethics committee.

RESULTS

Nine hundred and fifty one questionnaires were sent to GPs (see **Figure 1** for patient selection). Of those, 738 (77.6%) were returned, 704 (74.0%) met quality control standards and were not duplicates, and 696 (73.2%) could be included in the final analysis (8 had “uncertain” COPD diagnosis and no supporting documentation and were therefore excluded).

Among those included in the final analysis, additional evidence for the diagnosis of COPD was available for 272 patients. This represented 39.1% of the total study population, or 67.7% of the 402 patients with a confirmed COPD diagnosis in the study.

Overall, irrespective of the qualifying algorithm, 402 patients (57.8%) were considered to have a diagnosis of COPD based on reviewing physician judgment. **Table 1** shows the characteristics of the 696 patients included in the final analysis who were considered to possibly have COPD based on the inclusion criteria. On average, patients were in their mid-60s to early 70s across all algorithms. Approximately two-thirds of them were current smokers and one-quarter had a history of asthma. Generally, there were fewer patients with supporting information and cardiovascular co-morbidity in the less specific algorithms (4-8).

For peer review only

Table 1: Characteristics of the 696 patients included in the final study analysis

Algorithm		COPD Code + spirometry + COPD medication	COPD Code + spirometry	COPD Code + COPD medication	COPD Code only	Bronchitis + COPD medication	Bronchitis only	Symptoms + spirometry	Symptoms only
Number of individuals (%)		85 (100)	79 (100)	88 (100)	89 (100)	98 (100)	84 (100)	83 (100)	90 (100)
Number (%) with supporting info		46 (54.1)	44 (55.7)	48 (54.5)	40 (44.9)	32 (32.7)	18 (21.4)	30 (36.1)	14 (15.6)
Mean age (sd)		68.7 (11.3)	68.3 (11.7)	71.8 (10.5)	71.1 (10.4)	68.5 (13.1)	67.8 (13.4)	65.9 (11.9)	63.4 (14.1)
Male (%)		45 (52.9)	41 (51.9)	40 (45.5)	44 (49.4)	31 (31.6)	29 (34.5)	43 (51.8)	47 (52.2)
Current smoker (%)		49 (57.7)	50 (63.3)	55 (62.5)	47 (52.8)	66 (67.4)	61 (72.6)	48 (57.8)	58 (64.4)
GOLD stage* (n=465)	1	13 (16.3)	14 (18.0)	13 (15.9)	16 (20.5)	17 (28.8)	13 (35.1)	8 (20.0)	4 (36.4)
	2	43 (53.8)	48 (61.5)	41 (50.0)	46 (59.0)	31(52.5)	13 (35.1)	22 (55.0)	5 (45.5)
	3	18 (22.5)	13 (16.7)	22 (26.8)	12 (15.4)	9 (15.3)	8 (21.6)	8 (20.0)	2 (18.2)
	4	6 (7.5)	3 (3.9)	6 (7.3)	4 (5.1)	2 (3.4)	3 (8.1)	2 (5.0)	0 (0.0)
History of cardiovascular disease		20 (23.5)	22 (27.8)	17 (19.3)	18 (20.2)	20 (20.4)	17 (20.2)	14 (16.9)	9 (10.0)
History of asthma		18 (21.2)	20 (25.3)	16 (18.2)	15 (16.9)	27 (27.6)	23 (27.4)	19 (22.9)	23 (25.6)
Mean BMI (sd) (n=575)		N=83 27.5 (5.4)	N=78 26.7 (5.8)	N=86 26.4 (4.7)	N=87 27.8 (5.4)	N=98 27.4 (5.1)	N=85 28.1 (5.0)	N=41 27.1 (4.5)	N=17 27.6 (4.7)

*GOLD staging was ascertained from CPRD records or from supplementary information provided by GPs.

For peer review only

The number of patients diagnosed with COPD confirmed by the gold standard and the PPV for each algorithm is given in **Table 2**. The PPV was greatest for algorithms 1-4. Further data is available in the supplementary material regarding the effect of co-morbidities (**Table S1**), smoking status (**Table S2**) and GOLD staging (**Table S3**) on the performance of each algorithm.

Table 2: The PPV and proportion of patients diagnosed with COPD within each algorithm

Algorithm	Number of questionnaires sent out (n=951)	Number evaluable returned (n=696) (%)	Number with confirmed COPD	PPV and 95% CI
**COPD Code + spirometry + COPD medication**	119	85 (71.4)	76	89.4, 80.7-94.5
**COPD Code + spirometry**	119	79 (66.4)	67	83.8, 73.7-90.4
**COPD Code + COPD medication**	119	88 (73.9)	77	87.5, 78.6-93.0
**COPD Code only**	119	89 (74.8)	77	86.5, 77.5-92.3
**Bronchitis + COPD medication**	119	98 (82.4)	44	44.4, 34.8-54.5
**Bronchitis only**	119	84 (70.6)	26	29.5, 20.8-40.1
**Symptoms + spirometry**	119	83 (69.7)	37	43.5, 33.2-54.4
**Symptoms only**	118	90 (75.6)	11	12.2, 6.8-20.9

In a subset of 272 patients where additional evidence was available (in the form of spirometry print outs or hospital outpatient letters), we assessed accuracy of GP diagnosis of COPD. Overall, the PPV in this group was 95.0% (91.1-97.2), This is broken down by algorithm in **Table 3**. While the presence of supporting evidence improved the PPV in each group, algorithms 1-4 were still most accurate.

For peer review only - http://bmjopen.bmj.com/site/about/guidelines.xhtml

Table 3: PPV by algorithm where evidence was available to assess GP compared to physician diagnosis of COPD

Algorithm	Total number with evidence (N=272)	Number of patients with COPD confirmed by GOLD standard (N=220)	Number with COPD according to GP	PPV and 95% CI
**COPD Code + spirometry + COPD medication**	46	46	46	100
**COPD Code + spirometry**	44	35	33	94.3, 82.4-98.9
**COPD Code + COPD medication**	48	43	41	95.3, 82.4-98.9
**COPD Code only**	40	34	33	97.1, 80.2-99.6
**Bronchitis + COPD medication**	32	21	19	90.5, 66.0-97.9
**Bronchitis only**	18	12	11	91.7, 49.9-99.2
**Symptoms + spirometry**	30	21	20	95.2, 69.1-99.4
**Symptoms only**	14	8	6	75.0, 27.6-95.9

Post-hoc analysis

We repeated the analysis allowing individuals to be put into more than one algorithm and tested the PPV of COPD by algorithm relative to the gold standard where individuals were identified using only the presence of a specific COPD code (n=457) the PPV was identical to if they had a COPD code and evidence either in the form of spirometry or COPD medication prescription (n=454); PPV 83.0% (79.0-86.0) for both. In the majority of cases, where a specific COPD code had been assigned, there was additional evidence. Only 3 individuals had a specific COPD code with no additional evidence.

DISCUSSION

We tested the accuracy of eight different algorithms for identifying COPD within the CPRD among patients with suspected COPD (e.g., >35 years of age, smoking history, and recording of respiratory symptoms or COPD codes). The physician reviewer consensus was the GOLD standard. The best performing algorithm allowed an accurate ascertainment of 90% of patients as diagnosed with COPD. This consisted of a combination of a specific COPD code, more than one prescription of a COPD medication and spirometry (PPV 89.4, 95% CI: 80.7-94.5). The worst performing algorithm was represented by presence of

For peer review only - http://bmjopen.bmj.com/site/about/guidelines.xhtml

respiratory symptoms only (PPV12.2, 95%CI: 6.8-20.9). We found that any algorithm containing a specific COPD code performed better than those without (Algorithms 1-4). In a post hoc analysis, where we allowed individuals to populate more than one algorithm, we established that the use of additional information such as spirometry or medications in an algorithm to a specific COPD Read code alone did not increase the PPV. This suggests that the presence of a specific COPD Read code alone is sufficient to accurately identify patients with COPD from the database. Some study protocols require the presence of obstructive spirometry for identification of COPD patients; however, this study demonstrates that it may be unnecessary. This is particularly important as certain groups of individuals are less likely to have spirometry, namely women and patients <50 and >80 years of age. [8] This is also important as the minimal precision lost by not including spirometry and medications in the algorithm, allows an increase in the number of individuals who can potentially be included in a study. Using the whole of CPRD, we identified individuals with COPD using a specific COPD code only compared to a specific code plus medication and spirometry and found an increase in potential sample size for study of 10% using a specific COPD code only.

One of the advantages of this study was our ability to investigate both the accuracy of algorithms when identifying COPD patients within the CPRD, and accuracy of the actual GP diagnosis of COPD. When validating the GP diagnosis of COPD with a respiratory physician diagnosis as gold standard based on extra evidence provided by the GP, there was improved accuracy (PPV) across all algorithms, with algorithms 1-4 again performing best. This suggests that additional evidence is collected when the GP is reasonably certain that the patient has COPD. There was good concordance between GP and physician diagnosis suggesting respiratory consultant validation is not always needed. Where there was disagreement, this was usually because lung function did not meet criteria for COPD.

For peer review only

We found that the diagnostic accuracy of COPD decreased across all algorithms when patients also had a diagnosis of cardiovascular disease or asthma (Table S1 online supplement). When patients had a concomitant diagnosis of asthma, the presence of spirometry was particularly important to improve accuracy of COPD diagnosis. However, stratification led to smaller sample sizes, which could have impacted the precision of estimates. Unsurprisingly, the addition of the use of any inhaled COPD medication to the algorithm did not improve precision, most likely due to the overlap in medications used to treat both asthma and COPD (see online supplement). The algorithm accuracy was not affected by smoking status (current vs. ex-smoker) (Table S2 online supplement). We only included current or former smokers in our analysis, and cannot be sure of the validity of the results in a patient who has never smoked. Certainly in the UK, the majority of COPD is related to tobacco smoking and we hypothesized that fixed airflow obstruction in a non-smoker would most likely be due to chronic asthma.

When considering the severity of COPD by GOLD classification, algorithms 2 and 3, i.e. a specific COPD code and spirometry or COPD medications had the greatest accuracy for patients with mild disease (GOLD stage 1) (Table S3 online supplement). PPV increased with increasing disease severity. PPV increases with disease prevalence, and the prevalence of COPD increased moving from algorithm 8 to algorithm 1.

We considered the timing of diagnosis in view of an increased uptake of spirometry in primary care in more recent years and changes in QOF requirements over time during our study period (Table S4 online supplement). We found that algorithms 1-4 still had the greatest accuracy, but the PPV estimates were better for the post 2008 period than pre 2008 period (see supplementary material). Non-specific bronchitis codes and symptom codes were more likely to be used before 2008 rather than post. This may require consideration when developing retrospective cohorts for analysis and otherwise suggests QOF has had a

For peer review only - http://bmjopen.bmj.com/site/about/guidelines.xhtml

positive effect on the consistency of codes used for COPD diagnoses. However, our codes were more specific than QOF codes and some of the codes included in QOF were not included in our specific COPD codelist, but were included in the non-specific bronchitis codes. While we cannot comment on the accuracy of QOF codes, it is important to highlight that that some QOF codes are not disease specific and may not be a good way of identifying COPD patients from electronic health records as inevitably people without COPD will be included. It is also important to highlight that Read codes change over time with new codes added and some removed, and this needs consideration when identifying people with COPD.

There are limited COPD validation studies in electronic health records published in the literature to which we can compare our study. Soriano validated COPD in the GPRD in 2001, when OXMIS codes were still in use. [4] A Swedish study using a Swedish inpatient registry used ICD-9 and ICD-10 codes and identified COPD patients with similar accuracy.[16] A Canadian study in the Canadian primary care sentinel surveillance network used algorithms to identify several long term conditions and also had a PPV similar to ours for COPD [17]. However, all of these studies used different codes, algorithms and databases.

Our analysis has several limitations. Although the overall response rate for this study was acceptable (77.6%), the proportion of questionnaires accompanied with additional evidence allowing for adjudication was rather low. We chose PPV as the measure of accuracy in this study to allow us to determine the probability that a patient had COPD from their electronic health record. PPV is correlated with disease prevalence, and although it is strongly related to specificity, the actual estimates of specificity, sensitivity and negative predictive value cannot be determined from our data. Further, GP practices are self selecting with respect to their contribution to CPRD, however those practices appear to be representative of the UK population. Very few patients within contributing practices refuse to participate at an

For peer review only - http://bmjopen.bmj.com/site/about/guidelines.xhtml

individual level and this is not thought to bias the results. While CPRD is representative of the general population, as with all validation studies that require a response, we cannot be sure that our sample is representative of GPs who have not responded, although there is unlikely to be any difference. The amount of missing data among the responding questionnaires was low, suggesting reasonable data quality. One of the other limitations of this study is that patients had to be alive to be included; however it is unlikely that coding would be different for individuals who are no longer alive.

The algorithm that consisted of a specific COPD code, COPD medication and spirometry had the highest PPV; however the PPV was almost as high when a specific COPD code alone was used. The poorest performing algorithms were those that involved bronchitis codes or respiratory symptoms; we would not recommend using these algorithms to identify COPD patients. In conclusion, we have shown that the presence of a specific COPD Read code alone is sufficient to identify patients with COPD from electronic health records. Minimal precision lost by not including spirometry and medications in the algorithm, allows an increase in the number of individuals who can potentially be included in a study by up to 10%. However by not including spirometry in the definition, the ability to stage COPD according to GOLD stages may not be possible for all COPD patients included in a study.

For peer review only

Acknowledgments: none

Competing interests: none

Funding: This project was funded jointly by GSK and the MRC. JKQ is funded on a MRC Population Health Scientist Fellowship. This work was part funded by a MRC Industry Partnership award (grant number G0902135).

Contributorship statement: JKQ, HM, RLD, SE, KD and LS contributed to the conception and design, JKQ, HM, JH, to acquisition and analysis, JKQ, HM, RLD, HF, SE, JH, KD and LS to interpretation of data, and JKQ, HM, RLD, HF, SE, JH, KD and LS to the drafting of the manuscript. JKQ is responsible for the overall content as guarantor.

Data Sharing Statement: No additional data are available. We would be happy to provide the ISAC protocol to reviewers if required.

Figure legend

Figure 1: Study population

For peer review only

REFERENCES

1. National Statistics. www.statistics.gov.uk date accessed June 2013.

2. Shahab LM, Jarvis MJ, Britton J, et al. Prevalence, diagnosis and relation to tobacco dependence of chronic obstructive pulmonary disease in a nationally representative population sample *Thorax* 2006;61(12):1043

3. file:///C:/Users/ENCDJQUI/Downloads/Invisible-Lives-report.pdf Invisible Lives. Chronic Obstructive Pulmonary Disease (COPD) – finding the missing millions. British Lung Foundation. November 2007. Accessed February 2014.

4. Soriano JB, Maier WC, Visick G, et al. Validation of general practitioner-diagnosed COPD in the UK General Practice. *European Journal of Epidemiology* 2001;17:1075–1080

5. NICE guideline COPD http://guidance.nice.org.uk/CG101/QuickRefGuide/pdf/English date accessed January 2014.

6. Cooke CR, Joo MJ, Anderson SM et al. The validity of using ICD-9 codes and pharmacy records to identify patients with chronic obstructive pulmonary disease. *BMC Health Services Research* 2011;11:37

7. Smith CJP, Gribbin J, Challen KB, et al. The impact of the 2004 NICE guideline and 2003General Medical Services contract on COPD in primary care in the UK. *Q J Med* 2008;101:145–153.

8. http://www.nice.org.uk/aboutnice/qof/qof.jsp. Accessed January 2014.

For peer review only

For peer review only - http://bmjopen.bmj.com/site/about/guidelines.xhtml

9. CPRD http://www.cprd.com (accessed February 28,2013)

10. Williams T, VanStaa T, Padmanabhan S et al. Recent advances in utility and use of the General Practice Research Database as an example of a UK Primary Care Data resource. *Ther Adv Drug Saf* 2012;3:88–99.

11. Tate AR, Beloff N, Al-Radwan B et al. Exploiting the potential of large databases of electronic health records for research using rapid search algorithms and an intuitive query interface. *J Am Med Inform Assoc*. 2014;21(2):292-8.

12. Chisholm J. The Read clinical classification. BMJ, 1990. 300(6732):1092.

13. GOLD 2011 http://www.goldcopd.org/uploads/users/files/GOLD2011_Summary.pdf accessed Nov 2013.

14. https://mqi.ic.nhs.uk/IndicatorDefaultView.aspx?ref=1.09.03.07 (QOF COPD). Accessed Feb 2014.

15. http://www.nbmedical.com/pdf/keep_simple_qof_2014-2014.pdf accessed Feb 2014

16. Inghammar M, Engström G, Löfdahl C-G et al. Validation of a COPD diagnosis from the Swedish Inpatient Registry *Scandinavian Journal of Public Health*, 2012;40: 773–776

17. Kadhim-Saleh A, Green M, WilliamsonT et al. Validation of the Diagnostic Algorithms for 5 Chronic Conditions in the Canadian Primary Care Sentinel

For peer review only - http://bmjopen.bmj.com/site/about/guidelines.xhtml

Surveillance Network (CPCSSN): A Kingston Practice-based Research Network (PBRN) Report. *J Am Board Fam Med* 2013;26: 159–167

For peer review only

For peer review only - http://bmjopen.bmj.com/site/about/guidelines.xhtml

Validation of Chronic Obstructive Pulmonary Disease (COPD) recording in the Clinical Practice Research Datalink (CPRD-GOLD)

Jennifer K Quint[1], Hana Müllerova[2], Rachael L DiSantostefano[3], Harriet Forbes[1], Susan Eaton[4], John R Hurst[5], Kourtney Davis[2], Liam Smeeth[1].

[1]Department of Non-communicable disease Epidemiology, London School of Hygiene and Tropical Medicine, London, UK
[2] Respiratory Epidemiology, GlaxoSmithKline R&D, Uxbridge, UK
[3] Respiratory Epidemiology, GlaxoSmithKline R&D, RTP, NC USA
[4] Clinical Practice Research Datalink Group, Medicines and Healthcare products Regulatory Agency, London, UK
[5]UCL Respiratory Medicine, Royal Free Campus, University College London Medical School, London, UK

Corresponding author:
Dr. Jennifer K Quint
Department of Non-communicable disease Epidemiology
London School of Hygiene and Tropical Medicine
Keppel Street, London WC1E 7HT
Tel: 0207 927 2622
Email: **Jennifer.quint@lshtm.ac.uk**

Keywords: COPD, diagnosis of COPD, validation, spirometry, electronic health records

Word count: 3448

Contributorship statement: JKQ, HM, RLD, SE, KD and LS contributed to the conception and design, JKQ, HM, JH, to acquisition and analysis, JKQ, HM, RLD, HF, SE, JH, KD and LS to interpretation of data, and JKQ, HM, RLD, HF, SE, JH, KD and LS to the drafting of the manuscript. JKQ is responsible for the overall content as guarantor.

For peer review only - http://bmjopen.bmj.com/site/about/guidelines.xhtml

ABSTRACT

Objectives: The optimal method of identifying people with COPD from electronic primary care records is not known. We assessed the accuracy of different approaches using the Clinical Practice Research Datalink (CPRD), a UK electronic health record database.

Setting: Nine hundred and fifty one participants registered with a CPRD practice in the UK between 1st January 2004 and 31st December 2012. Individuals were selected for ≥1 of eight algorithms to identify people with COPD. General practitioners were sent a brief questionnaire and additional evidence to support a COPD diagnosis was requested. All information received was reviewed independently by two respiratory physicians whose opinion was taken as gold standard.

Primary outcome measure: The primary measure of accuracy was the positive predictive value (PPV); the proportion of people identified by each algorithm for whom COPD was confirmed.

Results: Nine hundred and fifty one questionnaires were sent and 738 (78%) returned. After quality control, 696 (73.2%) patients were included in the final analysis. All four algorithms including a specific COPD diagnostic code performed well. Using a diagnostic code alone the PPV was 86.5% (77.5% to 92.3%) while requiring a diagnosis plus spirometry plus specific medication the PPV was slightly higher at 89.4% (80.7% to 94.5%) but reduced case numbers by 10%. Algorithms without specific diagnostic codes had low PPVs (range 12.2% to 44.4%).

Conclusion: Patients with COPD can be accurately identified from UK primary care records using specific diagnostic codes. Requiring spirometry or COPD medications only marginally improved accuracy. The high accuracy applies since the introduction of an incentivised disease register for COPD as part of QOF (Quality and Outcomes Framework) in 2004.

For peer review only - http://bmjopen.bmj.com/site/about/guidelines.xhtml

Article Summary: This paper investigates how individuals with COPD can be accurately identified in electronic health records using specific COPD codes alone without additional information (i.e. presence of spirometry or respiratory medications). This information is essential for appropriate disease management, epidemiological research of COPD and other conditions, where COPD is an important co-morbidity.

Strengths and limitations of this study:

- We have shown that the presence of a specific COPD Read code alone is sufficient to identify patients with COPD from electronic health records. Minimal precision lost by not including spirometry and medications in the algorithm, allows an increase in the number of individuals who can potentially be included in a study by up to 10%.
- We were able to investigate both the accuracy of algorithms when identifying COPD patients within the CPRD, and accuracy of the actual GP diagnosis of COPD.
- The amount of missing data among the responding questionnaires was low, suggesting reasonable data quality.
- Although the overall response rate for this study was acceptable (77.6%), the proportion of questionnaires accompanied with additional evidence allowing for adjudication was lower.

No additional data are available. We would be happy to provide the ISAC protocol to reviewers if required.

For peer review only

INTRODUCTION

Chronic obstructive pulmonary disease (COPD) represents an enormous health burden worldwide. Currently COPD is the 4th leading cause of death and is predicted to become the 3rd by 2020.[1] There are approximately 835,000 people diagnosed with COPD in the UK and an estimated 2,200,000 people remain undiagnosed.[2,3]

Electronic health records in the UK provide an excellent resource in which to study COPD as they offer a large cohort size, the presence of disease severity indicators and long-term follow-up information on a patient's integrated medical history. Although multiple studies have been undertaken to investigate various aspects of COPD over the last 10 years in several electronic health record databases, there is no standard definition used to identify COPD in large databases and codelists used to identify patients with COPD vary by author. Over ten years ago, the diagnosis of COPD was validated in the Clinical Practice Research Datalink (CPRD-GOLD, formerly GPRD) using OXMIS codes.[4] This coding system is now obsolete in CPRD and Read codes are used as standard.

There is no single diagnostic test for COPD. The diagnosis of COPD relies on clinical judgement based on a combination of history, physical examination and confirmation of the presence of airflow obstruction using spirometry.[5] When retrospectively evaluating the accuracy of a COPD diagnosis, commonly used COPD definitions may misclassify patients as having COPD. Using multiple diagnostic codes in combination with pharmacy may improve the accuracy of identification of COPD patients.[6] Further, over the past 10 years the definition of COPD in clinical practice has evolved leading to changes in how COPD is diagnosed and the diagnosis recorded. With the introduction of the Quality and Outcomes Framework (QOF) codes for COPD in 2004 in England and Wales there are more codes available to identify COPD then there were previously. QOF is a voluntary incentive scheme for primary care physicians (GPs) in the UK which contains indicators against which GP

For peer review only

For peer review only - http://bmjopen.bmj.com/site/about/guidelines.xhtml

practices can score points and hence be rewarded for how well they care for patients. [7] Evaluation of airflow limitation using spirometry is used as standard to confirm COPD diagnosis and severity of COPD is part of the annual COPD patient review.[3,8] However, even spirometry if incorrectly performed or interpreted can lead to misdiagnosis (both over and under diagnosis of COPD) in approximately 20% of cases. It is also recognised that some subgroups of individuals (e.g. women and individuals under 50 and over 80) are less likely to have spirometry measured.[8]

This study aimed to improve our ability to identify patients with COPD within electronic health records. We used the CPRD, a widely used collection of computerised medical records which is commonly used for clinical and research purposes. CPRD is comparable to other electronic health record databases used in the UK. Our objective was to test the accuracy of different definitions of COPD in the CPRD using Positive Predictive Value (PPV), by comparing the database records with additional information provided by General Practitioners (GPs). This work is important for epidemiological research in COPD and diseases where COPD is an important co-morbidity as well as for clinical practice.

METHODS

Dataset

CPRD is the world's largest validated computerized database of anonymized longitudinal medical records for primary care. [9,10] Data comprise approximately 14 million patients with around 5.4 million of these being currently alive and registered from 660 primary care practices spread throughout the UK. Records are derived from a widely used GP software system and contain complete prescribing and coded diagnostic and clinical information as well as information on tests requested, laboratory results and referrals made at or following on from each consultation. [11]

For peer review only - http://bmjopen.bmj.com/site/about/guidelines.xhtml

Codelists and algorithms

Lists of medical codes (Read codes) specific and non-specific for COPD were created prior to the study initiation. Read codes are a hierarchical clinical coding system of over 80,000 terms that are used in general practice in the UK and are entered by the GP into Vision Software. [12] These data are then uploaded by CPRD after they have been processed and quality checked added to the CPRD database for research use. Specific COPD codes consisted of codes listing either COPD or COPD specific entities of emphysema (see supplementary material for specific codes included). Non-specific codes consisted of a variety of lung diseases that could potentially be COPD e.g. chronic bronchitis. Combinations of codelists and additional material in the form of the presence of spirometry or COPD medications (see supplementary material) were used to make up the eight algorithms. The first four definitions required a specific COPD diagnosis code, with the first three requiring additional documentation (e.g., medication and/or spirometry). The other four definitions required non-specific bronchitis or respiratory symptom codes, with the least specific definition requiring only respiratory symptom codes. Details regarding each algorithm can be found in the supplementary material. Briefly, the eight algorithms were defined as follows, from the expected most specific to most sensitive construct:

1. Specific COPD code and more than one prescription of a COPD medication and presence of spirometry (**COPD Code + spirometry + COPD medication)**
2. Specific COPD code and presence of spirometry (**COPD Code + spirometry)**
3. Specific COPD code and more than one prescription of a COPD medication (**COPD Code + COPD medication)**
4. Specific COPD code only (**COPD Code only)**
5. Non-specific bronchitis code and more than one prescription of a COPD medication (**Bronchitis + COPD medication)**
6. Non-specific bronchitis code only (**Bronchitis only)**

For peer review only - http://bmjopen.bmj.com/site/about/guidelines.xhtml

7. Respiratory symptoms and presence of spirometry. Respiratory symptoms consisted of persistent cough, sputum production, or dyspnoea. (**Symptoms + spirometry)**
8. Respiratory symptom definition only (**Symptoms only)**

The presence of spirometry for inclusion in the algorithm was based on an existence of a record of a specific value or a Read code for spirometry rather than examining the actual value. We were able to assess interpretation of spirometry in the subset of patients who had flow volume loops or clinic letters attached and where actual FEV_1 and FVC values were available in CPRD.

Study Population

The study population consisted of a random sample of individuals selected from all subjects registered in CPRD with cohort entry being on or after the 1st January 2004. At cohort entry, people included had to be: over 35 years old, with at least 1 year historical data, and a possible diagnosis of COPD defined as evidence of having ever smoked and a record of a least one specific or possible COPD code or respiratory symptoms suggestive of COPD. The presence of the algorithms was ascertained during a period between patient cohort entry and the 31st December 2012. Patients had to be alive within 4 months of the last collection date of CPRD data for the January 2013 data build for inclusion in the analysis so that CPRD could access their medical records and additional information. For the main analysis, a patient could contribute to one algorithm only. It was possible for an individual to be eligible for more than one algorithm depending upon the codes used in their medical record over the study period. Individuals were randomly selected from the algorithm with the fewest number of participants first and then removed from the cohort so they could not be selected for another algorithm.

For peer review only
For peer review only - http://bmjopen.bmj.com/site/about/guidelines.xhtml

CPRD mailed a short, structured questionnaire to GPs in charge of randomly selected patients requesting confirmation of COPD status as well as any available specific information from the individual's medical record including spirometry print outs and hospital respiratory outpatient letters (see supplementary material). Data were "twice encrypted" within CPRD to ensure anonymity; firstly between practices and CPRD and secondly from CPRD to researchers. In the questionnaire the GP was asked whether or not the patient had a diagnosis of COPD, what that diagnosis was based on, whether or not the patient had seen a respiratory physician and if they had any other respiratory diagnoses. A pilot set of 20 questionnaires were sent to GPs to assess the quality of the questionnaire. In total, 951 questionnaires were sent out assuming an 80% response rate.

Primary outcome

The primary outcome was identification of a diagnosis of COPD according to the pre-defined eight algorithms. The gold standard for the diagnosis of COPD was the decision made after respiratory physicians independently reviewed the evidence from the GP (questionnaire response with or without- additional evidence). Where they did not agree, a 3rd independent physician decided. Additionally, GP diagnosis of COPD was validated in a subset of patients where the GP provided supportive information including spirometry print-outs and hospital letters. This also allowed review of spirometry interpretation in some cases. Although we used two respiratory physicians independently as the gold standard for diagnosing COPD, this was done by reviewing the questionnaire sent to the GP (see appendix) as well as any additional information the GP sent which supported that diagnosis. This supporting information ranged from free text in the GP database to spirometry printouts done in the GP surgery to letters from secondary care. Therefore we were not solely relying on information from secondary care to make the diagnosis unless the GP decided to share that information. In this way we were able to include and validate the diagnosis of COPD in people who were not seen in secondary care.

For peer review only - http://bmjopen.bmj.com/site/about/guidelines.xhtml

Analysis

The primary analysis focused on the accuracy of identification of a COPD diagnosis in each of the pre-defined algorithms as defined by positive predicted value (PPV); i.e. the proportion of "true positives" (individuals with COPD) in each algorithm as determined by the gold standard. In addition, within each algorithm, where additional information was provided (lung function, hospital clinic letters), we calculated the accuracy of the GP diagnosis of COPD relative to the gold standard. This allowed review of spirometry interpretation in some cases.

We assessed the impact of commonly occurring co-morbidities on the accuracy of the pre-specified algorithms stratifying for cardiovascular co-morbidity, previous asthma diagnosis, smoking status and, where possible, Global Initiative for Chronic Obstructive Lung Disease (GOLD) staging of airflow limitation severity.[13] Cardiovascular co-morbidity included angina, history of myocardial infarction, previous coronary artery bypass graft / percutaneous coronary intervention or heart failure, but not hypertension due to its lack of overlap of symptoms that could mimic COPD. All covariates for stratification analysis were derived from information available up to cohort entry.

As a post hoc analysis individuals were eligible to be placed into multiple algorithms where possible, and the PPV was calculated for all individuals who had a specific COPD code compared to those with a specific COPD code and additional information (either spirometry or a COPD medication).

Assessment of possible trends in COPD diagnosis recording were also evaluated, including temporal trends in codes used and diagnostic specificity from 2004 to 2011. In addition, we compared our specific COPD codes to those recommended for use by QOF (see supplementary material); [14,15] H31% (excluding H3101 (smokers cough), H31y0 (chronic

For peer review only

For peer review only - http://bmjopen.bmj.com/site/about/guidelines.xhtml

tracheitis) and H3122 (acute exacerbation of COPD)) H32% H36-H3z (excluding H3y0 and H3y1).

Sample size calculation

Our sample size for each algorithm was chosen to achieve accuracy of the true positives or PPV ±0.08 based on the reviewing physician judgment as the gold standard. Assuming an estimated PPV of 0.85 for any one algorithm, we required a sample of at least 77 individuals in each algorithm to achieve the desired accuracy (95% CI ±0.08). All analyses were performed using STATA 13.

Ethics approval was obtained from ISAC (the Independent Scientific Advisory Committee overseeing CPRD); protocol 12_065 and the LSHTM ethics committee.

RESULTS

Nine hundred and fifty one questionnaires were sent to GPs (see **Figure 1** for patient selection). Of those, 738 (77.6%) were returned, 704 (74.0%) met quality control standards and were not duplicates, and 696 (73.2%) could be included in the final analysis (8 had "uncertain" COPD diagnosis and no supporting documentation and were therefore excluded).

Among those included in the final analysis, additional evidence for the diagnosis of COPD was available for 272 patients. This represented 39.1% of the total study population, or 67.7% of the 402 patients with a confirmed COPD diagnosis in the study.

Overall, irrespective of the qualifying algorithm, 402 patients (57.8%) were considered to have a diagnosis of COPD based on reviewing physician judgment. **Table 1** shows the characteristics of the 696 patients included in the final analysis who were considered to

For peer review only - http://bmjopen.bmj.com/site/about/guidelines.xhtml

For peer review only

possibly have COPD based on the inclusion criteria. On average, patients were in their mid-60s to early 70s across all algorithms. Approximately two-thirds of them were current smokers and one-quarter had a history of asthma. Generally, there were fewer patients with supporting information and cardiovascular co-morbidity in the less specific algorithms (4-8).

For peer review only

For peer review only - http://bmjopen.bmj.com/site/about/guidelines.xhtml

Table 1: Characteristics of the 696 patients included in the final study analysis

Algorithm		COPD Code + spirometry + COPD medication	COPD Code + spirometry	COPD Code + COPD medication	COPD Code only	Bronchitis + COPD medication	Bronchitis only	Symptoms + spirometry	Symptoms only
**Number of individuals (%)**		85 (100)	79 (100)	88 (100)	89 (100)	98 (100)	84 (100)	83 (100)	90 (100)
**Number (%) with supporting info**		46 (54.1)	44 (55.7)	48 (54.5)	40 (44.9)	32 (32.7)	18 (21.4)	30 (36.1)	14 (15.6)
**Mean age (sd)**		68.7 (11.3)	68.3 (11.7)	71.8 (10.5)	71.1 (10.4)	68.5 (13.1)	67.8 (13.4)	65.9 (11.9)	63.4 (14.1)
**Male (%)**		45 (52.9)	41 (51.9)	40 (45.5)	44 (49.4)	31 (31.6)	29 (34.5)	43 (51.8)	47 (52.2)
**Current smoker (%)**		49 (57.7)	50 (63.3)	55 (62.5)	47 (52.8)	66 (67.4)	61 (72.6)	48 (57.8)	58 (64.4)
**GOLD stage* (n=465)**	**1**	13 (16.3)	14 (18.0)	13 (15.9)	16 (20.5)	17 (28.8)	13 (35.1)	8 (20.0)	4 (36.4)
	**2**	43 (53.8)	48 (61.5)	41 (50.0)	46 (59.0)	31(52.5)	13 (35.1)	22 (55.0)	5 (45.5)
	**3**	18 (22.5)	13 (16.7)	22 (26.8)	12 (15.4)	9 (15.3)	8 (21.6)	8 (20.0)	2 (18.2)
	**4**	6 (7.5)	3 (3.9)	6 (7.3)	4 (5.1)	2 (3.4)	3 (8.1)	2 (5.0)	0 (0.0)
**History of cardiovascular disease**		20 (23.5)	22 (27.8)	17 (19.3)	18 (20.2)	20 (20.4)	17 (20.2)	14 (16.9)	9 (10.0)
**History of asthma**		18 (21.2)	20 (25.3)	16 (18.2)	15 (16.9)	27 (27.6)	23 (27.4)	19 (22.9)	23 (25.6)
**Mean BMI (sd) (n=575)**		N=83 27.5 (5.4)	N=78 26.7 (5.8)	N=86 26.4 (4.7)	N=87 27.8 (5.4)	N=98 27.4 (5.1)	N=85 28.1 (5.0)	N=41 27.1 (4.5)	N=17 27.6 (4.7)

*GOLD staging was ascertained from CPRD records or from supplementary information provided by GPs.

For peer review only - http://bmjopen.bmj.com/site/about/guidelines.xhtml

The number of patients diagnosed with COPD confirmed by the gold standard and the PPV for each algorithm is given in **Table 2**. The PPV was greatest for algorithms 1-4. Further data is available in the supplementary material regarding the effect of co-morbidities (**Table S1**), smoking status (**Table S2**) and GOLD staging (**Table S3**) on the performance of each algorithm.

Table 2: The PPV and proportion of patients diagnosed with COPD within each algorithm

**Algorithm**	**Number of questionnaires sent out (n=951)**	**Number evaluable returned (n=696) (%)**	**Number with confirmed COPD**	**PPV and 95% CI**
**COPD Code + spirometry + COPD medication**	119	85 (71.4)	76	89.4, 80.7-94.5
**COPD Code + spirometry**	119	79 (66.4)	67	83.8, 73.7-90.4
**COPD Code + COPD medication**	119	88 (73.9)	77	87.5, 78.6-93.0
**COPD Code only**	119	89 (74.8)	77	86.5, 77.5-92.3
**Bronchitis + COPD medication**	119	98 (82.4)	44	44.4, 34.8-54.5
**Bronchitis only**	119	84 (70.6)	26	29.5, 20.8-40.1
**Symptoms + spirometry**	119	83 (69.7)	37	43.5, 33.2-54.4
**Symptoms only**	118	90 (75.6)	11	12.2, 6.8-20.9

In a subset of 272 patients where additional evidence was available (in the form of spirometry print outs or hospital outpatient letters), we assessed accuracy of GP diagnosis of COPD. Overall, the PPV in this group was 95.0% (91.1-97.2), This is broken down by algorithm in **Table 3**. While the presence of supporting evidence improved the PPV in each group, algorithms 1-4 were still most accurate.

For peer review only - http://bmjopen.bmj.com/site/about/guidelines.xhtml

Table 3: PPV by algorithm where evidence was available to assess GP compared to physician diagnosis of COPD

Algorithm	Total number with evidence (N=272)	Number of patients with COPD confirmed by GOLD standard (N=220)	Number with COPD according to GP	PPV and 95% CI
**COPD Code + spirometry + COPD medication**	46	46	46	100
**COPD Code + spirometry**	44	35	33	94.3, 82.4-98.9
**COPD Code + COPD medication**	48	43	41	95.3, 82.4-98.9
**COPD Code only**	40	34	33	97.1, 80.2-99.6
**Bronchitis + COPD medication**	32	21	19	90.5, 66.0-97.9
**Bronchitis only**	18	12	11	91.7, 49.9-99.2
**Symptoms + spirometry**	30	21	20	95.2, 69.1-99.4
**Symptoms only**	14	8	6	75.0, 27.6-95.9

Post-hoc analysis

We repeated the analysis allowing individuals to be put into more than one algorithm and tested the PPV of COPD by algorithm relative to the gold standard where individuals were identified using only the presence of a specific COPD code (n=457) the PPV was identical to if they had a COPD code and evidence either in the form of spirometry or COPD medication prescription (n=454); PPV 83.0% (79.0-86.0) for both. In the majority of cases, where a specific COPD code had been assigned, there was additional evidence. Only 3 individuals had a specific COPD code with no additional evidence.

DISCUSSION

We tested the accuracy of eight different algorithms for identifying COPD within the CPRD among patients with suspected COPD (e.g., >35 years of age, smoking history, and recording of respiratory symptoms or COPD codes). The physician reviewer consensus was the GOLD standard. The best performing algorithm allowed an accurate ascertainment of 90% of patients as diagnosed with COPD. This consisted of a combination of a specific COPD code, more than one prescription of a COPD medication and spirometry (PPV 89.4, 95% CI: 80.7-94.5). The worst performing algorithm was represented by presence of

For peer review only

For peer review only - http://bmjopen.bmj.com/site/about/guidelines.xhtml

respiratory symptoms only (PPV12.2, 95%CI: 6.8-20.9). We found that any algorithm containing a specific COPD code performed better than those without (Algorithms 1-4). In a post hoc analysis, where we allowed individuals to populate more than one algorithm, we established that the use of additional information such as spirometry or medications in an algorithm to a specific COPD Read code alone did not increase the PPV. This suggests that the presence of a specific COPD Read code alone is sufficient to accurately identify patients with COPD from the database. Some study protocols require the presence of obstructive spirometry for identification of COPD patients; however, this study demonstrates that it may be unnecessary. This is particularly important as certain groups of individuals are less likely to have spirometry, namely women and patients <50 and >80 years of age. [8] This is also important as the minimal precision lost by not including spirometry and medications in the algorithm, allows an increase in the number of individuals who can potentially be included in a study. Using the whole of CPRD, we identified individuals with COPD using a specific COPD code only compared to a specific code plus medication and spirometry and found an increase in potential sample size for study of 10% using a specific COPD code only.

One of the advantages of this study was our ability to investigate both the accuracy of algorithms when identifying COPD patients within the CPRD, and accuracy of the actual GP diagnosis of COPD. When validating the GP diagnosis of COPD with a respiratory physician diagnosis as gold standard based on extra evidence provided by the GP, there was improved accuracy (PPV) across all algorithms, with algorithms 1-4 again performing best. This suggests that additional evidence is collected when the GP is reasonably certain that the patient has COPD. There was good concordance between GP and physician diagnosis suggesting respiratory consultant validation is not always needed. Where there was disagreement, this was usually because lung function did not meet criteria for COPD.

For peer review only - http://bmjopen.bmj.com/site/about/guidelines.xhtml

We found that the diagnostic accuracy of COPD decreased across all algorithms when patients also had a diagnosis of cardiovascular disease or asthma (Table S1 online supplement). When patients had a concomitant diagnosis of asthma, the presence of spirometry was particularly important to improve accuracy of COPD diagnosis. However, stratification led to smaller sample sizes, which could have impacted the precision of estimates. Unsurprisingly, the addition of the use of any inhaled COPD medication to the algorithm did not improve precision, most likely due to the overlap in medications used to treat both asthma and COPD (see online supplement). The algorithm accuracy was not affected by smoking status (current vs. ex-smoker) (Table S2 online supplement). We only included current or former smokers in our analysis, and cannot be sure of the validity of the results in a patient who has never smoked. Certainly in the UK, the majority of COPD is related to tobacco smoking and we hypothesized that fixed airflow obstruction in a non-smoker would most likely be due to chronic asthma.

When considering the severity of COPD by GOLD classification, algorithms 2 and 3, i.e. a specific COPD code and spirometry or COPD medications had the greatest accuracy for patients with mild disease (GOLD stage 1) (Table S3 online supplement). PPV increased with increasing disease severity. PPV increases with disease prevalence, and the prevalence of COPD increased moving from algorithm 8 to algorithm 1.

We considered the timing of diagnosis in view of an increased uptake of spirometry in primary care in more recent years and changes in QOF requirements over time during our study period (Table S4 online supplement). We found that algorithms 1-4 still had the greatest accuracy, but the PPV estimates were better for the post 2008 period than pre 2008 period (see supplementary material). Non-specific bronchitis codes and symptom codes were more likely to be used before 2008 rather than post. This may require consideration when developing retrospective cohorts for analysis and otherwise suggests QOF has had a

For peer review only - http://bmjopen.bmj.com/site/about/guidelines.xhtml

positive effect on the consistency of codes used for COPD diagnoses. However, our codes were more specific than QOF codes and some of the codes included in QOF were not included in our specific COPD codelist, but were included in the non-specific bronchitis codes. While we cannot comment on the accuracy of QOF codes, it is important to highlight that that some QOF codes are not disease specific and may not be a good way of identifying COPD patients from electronic health records as inevitably people without COPD will be included. It is also important to highlight that Read codes change over time with new codes added and some removed, and this needs consideration when identifying people with COPD.

There are limited COPD validation studies in electronic health records published in the literature to which we can compare our study. Soriano validated COPD in the GPRD in 2001, when OXMIS codes were still in use. [4] A Swedish study using a Swedish inpatient registry used ICD-9 and ICD-10 codes and identified COPD patients with similar accuracy.[16] A Canadian study in the Canadian primary care sentinel surveillance network used algorithms to identify several long term conditions and also had a PPV similar to ours for COPD [17]. However, all of these studies used different codes, algorithms and databases.

Our analysis has several limitations. Although the overall response rate for this study was acceptable (77.6%), the proportion of questionnaires accompanied with additional evidence allowing for adjudication was rather low. We chose PPV as the measure of accuracy in this study to allow us to determine the probability that a patient had COPD from their electronic health record. PPV is correlated with disease prevalence, and although it is strongly related to specificity, the actual estimates of specificity, sensitivity and negative predictive value cannot be determined from our data. Further, GP practices are self selecting with respect to their contribution to CPRD, however those practices appear to be representative of the UK population. Very few patients within contributing practices refuse to participate at an

For peer review only

individual level and this is not thought to bias the results. While CPRD is representative of the general population, as with all validation studies that require a response, we cannot be sure that our sample is representative of GPs who have not responded, although there is unlikely to be any difference. The amount of missing data among the responding questionnaires was low, suggesting reasonable data quality. One of the other limitations of this study is that patients had to be alive to be included; however it is unlikely that coding would be different for individuals who are no longer alive.

The algorithm that consisted of a specific COPD code, COPD medication and spirometry had the highest PPV; however the PPV was almost as high when a specific COPD code alone was used. The poorest performing algorithms were those that involved bronchitis codes or respiratory symptoms; we would not recommend using these algorithms to identify COPD patients. In conclusion, we have shown that the presence of a specific COPD Read code alone is sufficient to identify patients with COPD from electronic health records. Minimal precision lost by not including spirometry and medications in the algorithm, allows an increase in the number of individuals who can potentially be included in a study by up to 10%. However by not including spirometry in the definition, the ability to stage COPD according to GOLD stages may not be possible for all COPD patients included in a study.

Acknowledgments: none

Competing interests: none

Funding: This project was funded jointly by GSK and the MRC. JKQ is funded on a MRC Population Health Scientist Fellowship. This work was part funded by a MRC Industry Partnership award (grant number G0902135).

Figure 1: Study population

For peer review only - http://bmjopen.bmj.com/site/about/guidelines.xhtml

REFERENCES

1. National Statistics. www.statistics.gov.uk date accessed June 2013.

2. Shahab LM, Jarvis MJ, Britton J, et al. Prevalence, diagnosis and relation to tobacco dependence of chronic obstructive pulmonary disease in a nationally representative population sample *Thorax* 2006;61(12):1043

3. file:///C:/Users/ENCDJQUI/Downloads/Invisible-Lives-report.pdf Invisible Lives. Chronic Obstructive Pulmonary Disease (COPD) – finding the missing millions. British Lung Foundation. November 2007. Accessed February 2014.

4. Soriano JB, Maier WC, Visick G, et al. Validation of general practitioner-diagnosed COPD in the UK General Practice. *European Journal of Epidemiology* 2001;17:1075–1080

5. NICE guideline COPD http://guidance.nice.org.uk/CG101/QuickRefGuide/pdf/English date accessed January 2014.

6. Cooke CR, Joo MJ, Anderson SM et al. The validity of using ICD-9 codes and pharmacy records to identify patients with chronic obstructive pulmonary disease. *BMC Health Services Research* 2011;11:37

7. Smith CJP, Gribbin J, Challen KB, et al. The impact of the 2004 NICE guideline and 2003General Medical Services contract on COPD in primary care in the UK. *Q J Med* 2008;101:145–153.

For peer review only - http://bmjopen.bmj.com/site/about/guidelines.xhtml

8. http://www.nice.org.uk/aboutnice/qof/qof.jsp. Accessed January 2014.

9. CPRD http://www.cprd.com (accessed February 28,2013)

10. Williams T, VanStaa T, Padmanabhan S et al. Recent advances in utility and use of the General Practice Research Database as an example of a UK Primary Care Data resource. *Ther Adv Drug Saf* 2012;3:88–99.

11. Tate AR, Beloff N, Al-Radwan B et al. Exploiting the potential of large databases of electronic health records for research using rapid search algorithms and an intuitive query interface. *J Am Med Inform Assoc*. 2014;21(2):292-8.

12. Chisholm J. The Read clinical classification. BMJ, 1990. 300(6732):1092.

13. GOLD 2011 http://www.goldcopd.org/uploads/users/files/GOLD2011_Summary.pdf accessed Nov 2013.

14. https://mqi.ic.nhs.uk/IndicatorDefaultView.aspx?ref=1.09.03.07 (QOF COPD). Accessed Feb 2014.

15. http://www.nbmedical.com/pdf/keep_simple_qof_2014-2014.pdf accessed Feb 2014

16. Inghammar M, Engström G, Löfdahl C-G et al. Validation of a COPD diagnosis from the Swedish Inpatient Registry *Scandinavian Journal of Public Health*, 2012;40: 773–776

For peer review only

17. Kadhim-Saleh A, Green M, WilliamsonT et al. Validation of the Diagnostic Algorithms for 5 Chronic Conditions in the Canadian Primary Care Sentinel Surveillance Network (CPCSSN): A Kingston Practice-based Research Network (PBRN) Report. *J Am Board Fam Med* 2013;26: 159–167

For peer review only

For peer review only - http://bmjopen.bmj.com/site/about/guidelines.xhtml

Figure 1: Study sample

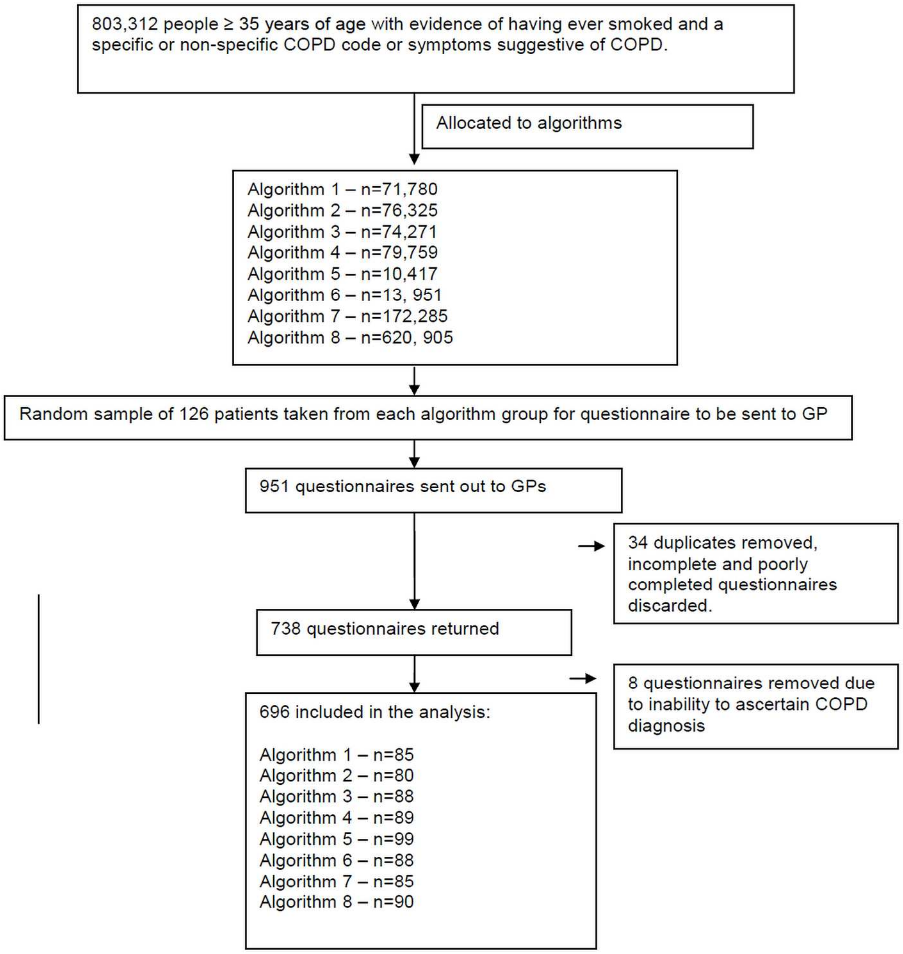

228x248mm (300 x 300 DPI)

Supplementary information

Algorithms

1. Specific COPD code and more than one prescription of a COPD medication and presence of spirometry. The first COPD medication had to be prescribed within 4 weeks of the COPD diagnostic code.
2. Specific COPD code and presence of spirometry
3. Specific COPD code and more than one prescription of a COPD medication. The first COPD medication had to be prescribed within 4 weeks of the COPD diagnostic code.
4. Specific COPD code only
5. Non-specific bronchitis codes and more than one prescription of a COPD medication. The first COPD medication had to be prescribed within 4 weeks of the COPD diagnostic code.
6. Non-specific bronchitis codes only
7. Respiratory symptoms and presence of spirometry. Respiratory symptoms consisted of persistent cough, sputum production, or dyspnoea
8. Respiratory symptom definition only

For peer review only

Specific COPD codes

medcode	term
18476	COPD follow-up
45771	Chronic obstructive pulmonary disease does not disturb sleep
4084	Airways obstructn irreversible
794	Emphysema
998	Chronic obstructive airways disease
1001	Chronic obstructive pulmonary disease
5710	Chronic obstructive airways disease NOS
9520	Chronic obstructive pulmonary disease monitoring
9876	Severe chronic obstructive pulmonary disease
10802	Moderate chronic obstructive pulmonary disease
10863	Mild chronic obstructive pulmonary disease
10980	Centrilobular emphysema
11287	Chronic obstructive pulmonary disease annual review
14798	Emphysematous bronchitis
18621	Chronic obstructive pulmonary disease follow-up
18792	Chronic obstructive pulmonary disease monitoring admin
23492	Chronic bullous emphysema NOS
26018	Chronic obstructive pulmonary disease monitoring by nurse
26306	Chronic bullous emphysema
28755	Chronic obstructive pulmonary disease monitoring 1st letter
33450	Emphysema NOS
34202	Chronic obstructive pulmonary disease monitoring 2nd letter
34215	Chronic obstructive pulmonary disease monitoring 3rd letter
37247	Chronic obstructive pulmonary disease NOS
37371	Chronic obstructive pulmonary disease monitoring due
44525	Obstructive chronic bronchitis NOS
45998	Chronic obstructive pulmonary disease monitoring by doctor
93568	Very severe chronic obstructive pulmonary disease
12166	Other specified chronic obstructive airways disease

38074 Chronic obstructive pulmonary disease monitor phone invite
42258 Chronic obstructive pulmonary disease monitoring verb invite
42313 Health education - chronic obstructive pulmonary disease
45770 Chronic obstructive pulmonary disease disturbs sleep
45777 Chronic obstructive pulmonary disease clini management plan

COPD medication codes

prodcode	productname
8	salbutamol 100micrograms/dose inhaler
17	salbutamol 100micrograms/dose inhaler cfc free
31	ventolin 100microgram/inhalation inhalation powder (glaxo wellcome uk ltd)
38	beclometasone 100micrograms/dose inhaler
99	becotide 100 inhaler (glaxosmithkline uk ltd)
218	aminophylline 100 mg cap
235	bricanyl 250micrograms/dose inhaler (astrazeneca uk ltd)
273	theophylline 200 mg cap
282	salbutamol 2mg/5ml oral solution sugar free
454	pulmicort 200microgram inhaler (astrazeneca uk ltd)
465	salmeterol 25micrograms/dose inhaler
510	ventolin 5mg/ml respirator solution (glaxosmithkline uk ltd)
549	serevent 25micrograms/dose inhaler (glaxosmithkline uk ltd)
555	aminophylline 225mg modified-release tablets
590	phyllocontin continus 225mg tablets (napp pharmaceuticals ltd)
638	seretide 250 accuhaler (glaxosmithkline uk ltd)
665	seretide 100 accuhaler (glaxosmithkline uk ltd)
674	ventolin 2.5mg nebules (glaxosmithkline uk ltd)
696	salbutamol 8mg modified-release capsules
719	salmeterol 50micrograms/dose dry powder inhaler
746	tiotropium 18 microgram capsule
752	carbocisteine 375mg capsules
856	ventolin 2mg/5ml syrup (glaxosmithkline uk ltd)
860	salbutamol 4mg tablets
862	salbulin inhalation powder (3m health care ltd)
863	slo-phyllin 125mg capsule (lipha pharmaceuticals ltd)
879	theophylline 125mg modified-release capsules
880	theophylline 60mg modified-release capsules
881	salbutamol 2mg tablets
882	salbutamol 200microgram inhalation powder capsules
883	becodisks 200microgram disc (allen & hanburys ltd)
895	beclazone 100 easi-breathe inhaler (teva uk ltd)
896	becotide easi-breathe 100microgram/actuation pressurised inhalation (allen & hanburys ltd)
898	ventolin evohaler 100 100microgram/inhalation pressurised inhalation (glaxo wellcome uk ltd)
907	bricanyl turbohaler 500 500microgram turbohaler (astrazeneca uk ltd)
908	pulmicort 400 turbohaler (astrazeneca uk ltd)
909	budesonide 200micrograms/dose inhaler
910	serevent diskhaler 50microgram inhalation powder (glaxo wellcome uk ltd)
911	flixotide accuhaler 250 250microgram/inhalation inhalation powder (allen & hanburys ltd)
947	budesonide 50micrograms/actuation refill canister
956	pulmicort 200 turbohaler (astrazeneca uk ltd)

For peer review only

957 salamol easi-breathe 100microgram/actuation pressurised inhalation (ivax pharmaceuticals uk ltd)
958 ventolin easi-breathe 100microgram/actuation pressurised inhalation (allen & hanburys ltd)
959 budesonide 50micrograms/dose inhaler
960 pulmicort 100 turbohaler (astrazeneca uk ltd)
987 ventolin 4mg tablet (allen & hanburys ltd)
1093 salamol 100microgram/actuation inhalation powder (ivax pharmaceuticals uk ltd)
1097 slo-phyllin 60mg capsule (lipha pharmaceuticals ltd)
1100 beclazone 100 inhaler (teva uk ltd)
1236 becloforte 250micrograms/dose inhaler (glaxosmithkline uk ltd)
1242 beclometasone 250micrograms/dose inhaler
1243 beclazone 250 easi-breathe inhaler (teva uk ltd)
1258 becotide 200 inhaler (glaxosmithkline uk ltd)
1259 beclometasone 200micrograms/dose inhaler
1269 becotide 50microgram/ml nebuliser liquid (allen & hanburys ltd)
1346 salbutamol 0.05mg/ml injection
1406 becotide 50 inhaler (glaxosmithkline uk ltd)
1409 ipratropium bromide 20micrograms/dose inhaler
1410 ipratropium bromide 0.25mg/ml
1411 ipratropium bromide 250micrograms/ml
1412 flixotide 250microgram/actuation inhalation powder (allen & hanburys ltd)
1414 salamol 5mg/2.5ml nebuliser liquid steri-neb unit dose vials (teva uk ltd)
1415 steri-neb ipratropium 250microgram/ml nebuliser liquid (ivax pharmaceuticals uk ltd)
1423 uniphyllin continus 200mg tablets (napp pharmaceuticals ltd)
1424 flixotide 250microgram disc (allen & hanburys ltd)
1426 flixotide 500microgram disc (allen & hanburys ltd)
1518 flixotide 50microgram/actuation inhalation powder (allen & hanburys ltd)
1537 becotide 200microgram rotacaps (glaxosmithkline uk ltd)
1551 beclazone 250 inhaler (teva uk ltd)
1552 becloforte easi-breathe 250microgram/actuation pressurised inhalation (allen & hanburys ltd)
1619 terbutaline 500micrograms/dose dry powder inhaler
1620 terbutaline 250micrograms/dose inhaler
1628 terbutaline 250micrograms/actuation refill canister
1630 salbutamol 2.5mg/2.5ml nebuliser liquid unit dose vials
1635 salbuvent 2mg/5ml oral solution (pharmacia ltd)
1642 budesonide 400micrograms/dose dry powder inhaler
1676 flixotide 125microgram/actuation inhalation powder (allen & hanburys ltd)
1680 pulmicort ls 50micrograms/dose inhaler (astrazeneca uk ltd)
1698 salbutamol 100micrograms/dose breath actuated inhaler
1711 salbutamol 5mg/2.5ml nebuliser liquid unit dose vials
1725 beclazone 50 easi-breathe inhaler (teva uk ltd)
1727 becotide easi-breathe 50microgram/actuation pressurised inhalation (allen & hanburys ltd)
1734 beclometasone 100micrograms/dose breath actuated inhaler
1741 salbutamol 100micrograms/dose breath actuated inhaler cfc free
1833 theophylline 200mg modified-release tablets
1834 theophylline 400mg modified-release tablets
1861 aerobec 100 autohaler (meda pharmaceuticals ltd)
1882 ventodisks 200microgram/blister disc (allen & hanburys ltd)
1885 beclazone 200 inhaler (teva uk ltd)
1950 ventodisks 400microgram/blister disc (allen & hanburys ltd)
1951 becodisks 400microgram disc (allen & hanburys ltd)
1952 ventolin 400microgram rotacaps (glaxosmithkline uk ltd)

For peer review only

1956 pulmicort 1mg respules (astrazeneca uk ltd)
1957 ventolin 5mg nebules (glaxosmithkline uk ltd)
1959 pulmicort 0.5mg respules (astrazeneca uk ltd)
1972 oxygen bp size af lightweight gas 1360 litres
1974 oxis 12 turbohaler (astrazeneca uk ltd)
1975 oxis 6 turbohaler (astrazeneca uk ltd)
2044 prednisone 2.5 mg tab
2092 budesonide 200micrograms/dose dry powder inhaler
2124 pulmicort refil 200 mcg inh
2125 pulmicort 200microgram refill canister (astrazeneca uk ltd)
2147 theophylline 250mg modified-release capsules
2148 beclometasone 400microgram disc
2149 steri-neb salamol 2.5 mg inh
2152 ipratropium bromide with salbutamol 20mcg + 100mcg
2159 aerobec 50 autohaler (meda pharmaceuticals ltd)
2160 beclometasone 50micrograms/dose breath actuated inhaler
2224 serevent 50micrograms/dose accuhaler (glaxosmithkline uk ltd)
2229 becodisks 100microgram disc (allen & hanburys ltd)
2282 fluticasone 500micrograms/dose dry powder inhaler
2335 qvar 100 inhaler (teva uk ltd)
2368 prednisolone 2.5mg tablet
2390 prednisolone e/c 1 mg tab
2395 salbutamol 2 mg/5ml syr
2437 oxitropium bromide 100micrograms/dose inhaler
2440 flixotide accuhaler 500 500microgram/inhalation inhalation powder (allen & hanburys ltd)
2510 oxygen bp gas 1280 litres
2600 beclometasone 250micrograms/dose breath actuated inhaler
2655 airomir 100micrograms/dose inhaler (teva uk ltd)
2723 fluticasone 25micrograms/dose inhaler
2757 slo-phyllin 250mg capsule (lipha pharmaceuticals ltd)
2758 bricanyl refill canister (astrazeneca uk ltd)
2799 prednisolone 10 mg tab
2850 salbutamol 400microgram inhalation powder capsules
2851 ventolin 200microgram rotacaps (glaxosmithkline uk ltd)
2869 salbutamol 8mg modified-release tablets
2892 becloforte 400microgram disks (glaxosmithkline uk ltd)
2893 beclometasone 200micrograms disc
2949 prednisone 5mg tablets
2951 fluticasone 250microgram/actuation pressurised inhalation
2978 salbutamol 200micrograms/dose dry powder inhaler
2992 beclazone 50 inhaler (teva uk ltd)
2995 nuelin sa 175mg tablets (meda pharmaceuticals ltd)
3018 beclometasone 50micrograms/dose inhaler
3059 prednisolone 50 mg tab
3065 bextasol inhalation powder (allen & hanburys ltd)
3075 becotide 400microgram rotacaps (glaxosmithkline uk ltd)
3119 becloforte integra 250microgram/actuation inhaler with compact spacer (glaxo laboratories ltd)
3150 beclometasone 100micrograms/actuation extrafine particle cfc free inhaler
3163 salbutamol 200micrograms disc
3188 pulmicort complete 50 mcg inh
3189 salbuvent inh inh
3220 qvar 50 autohaler (teva uk ltd)
3254 salbulin 4mg tablet (3m health care ltd)

For peer review only

3289 flixotide 25micrograms/dose inhaler (glaxosmithkline uk ltd)
3297 salmeterol 50micrograms disc
3322 aerolin inh 400 100 mcg aer
3345 sintisone tablet (pharmacia ltd)
3347 oxygen bp size f gas 1360 litres
3363 becloforte 400microgram disks with diskhaler (glaxosmithkline uk ltd)
3388 theophylline 175mg modified-release tablets
3442 pulmicort complete 200 mcg inh
3443 salbutamol 100microgram/inhalation spacehaler (celltech pharma europe ltd)
3534 bricanyl 5mg tablets (astrazeneca uk ltd)
3546 qvar 50 inhaler (teva uk ltd)
3556 beclometasone 50micrograms with salbutamol 100micrograms/inhalation inhaler
3557 prednisone 1mg tablets
3570 budesonide 200micrograms/actuation refill canister
3584 bricanyl 1.5mg/5ml syrup (astrazeneca uk ltd)
3666 seretide 500 accuhaler (glaxosmithkline uk ltd)
3743 filair 50 inhaler (meda pharmaceuticals ltd)
3753 flixotide diskhaler-community pack 250 mcg
3758 pulmadil inhalation powder (3m health care ltd)
3763 terbutaline respules inh
3764 bricanyl respules (5mg/2ml) 2.5 mg/ml inh
3786 fenoterol 100micrograms/dose / ipratropium 40micrograms/dose inhaler
3838 salbutamol 400mcg/beclometh.100mcg r/cap inh
3927 filair 100 inhaler (meda pharmaceuticals ltd)
3947 becotide 100microgram rotacaps (glaxosmithkline uk ltd)
3988 flixotide diskhaler-community pack 100 mcg
3989 flixotide 100microgram disc (allen & hanburys ltd)
3993 filair forte 250micrograms/dose inhaler (meda pharmaceuticals ltd)
3994 salbutamol 4mg modified-release tablets
4055 salbulin 2mg/5ml oral solution (3m health care ltd)
4131 fluticasone 100microgram disc
4132 fluticasone 125microgram/actuation pressurised inhalation
4165 zithromax 250mg capsules (pfizer ltd)
4171 ventolin 2mg tablet (allen & hanburys ltd)
4222 bricanyl 10mg/ml respirator solution (astrazeneca uk ltd)
4268 ipratropium bromide 40micrograms/dose inhaler
4365 beclometasone 100micrograms disc
4412 oxygen bp size g gas 3400 litres
4413 qvar 100 autohaler (teva uk ltd)
4497 ventolin accuhaler 200 200microgram/actuation inhalation powder (glaxo wellcome uk ltd)
4499 aerobec 250microgram/actuation pressurised inhalation (meda pharmaceuticals ltd)
4514 aminophylline 350mg modified-release tablets
4538 oxygen bp size pd gas 300 litres
4541 bricanyl sa 7.5mg tablets (astrazeneca uk ltd)
4545 pulmicort ls 50microgram refill canister (astrazeneca uk ltd)
4593 theophylline 125mg tablets
4601 asmabec 100 clickhaler (focus pharmaceuticals ltd)
4634 salamol 2.5mg/2.5ml nebuliser liquid steri-neb unit dose vials (teva uk ltd)
4640 bricanyl 5mg/2ml nebuliser liquid (astrazeneca uk ltd)
4665 salbulin 100micrograms/dose inhaler (3m health care ltd)
4688 fluticasone 50microgram/actuation pressurised inhalation
4759 beclometasone 100microgram inhalation powder capsules
4791 oxygen bp size c gas 170 litres
4801 budesonide 500micrograms/2ml nebuliser liquid unit dose vials

For peer review only

4803 beclazone 250microgram/actuation inhalation powder (actavis uk ltd)
4842 fenoterol 100microgram/actuation inhaler
4908 ventolin rotahaler (glaxosmithkline uk ltd)
4926 flixotide accuhaler 100 100microgram/inhalation inhalation powder (allen & hanburys ltd)
4942 budesonide 1mg/2ml nebuliser liquid unit dose vials
5057 azithromycin 200mg/5ml oral suspension
5116 azithromycin 250mg capsules
5143 seretide 50 evohaler (glaxosmithkline uk ltd)
5161 seretide 125 evohaler (glaxosmithkline uk ltd)
5170 salamol 100micrograms/dose inhaler cfc free (teva uk ltd)
5172 seretide 250 evohaler (glaxosmithkline uk ltd)
5185 fenoterol 200micrograms/dose inhaler
5223 fluticasone 50micrograms/dose inhaler cfc free
5261 nuelin sa 250 tablets (meda pharmaceuticals ltd)
5308 terbutaline 5mg/2ml nebuliser liquid unit dose vials
5309 flixotide 50micrograms/dose evohaler (glaxosmithkline uk ltd)
5335 zithromax 500mg tablets (pfizer ltd)
5453 uniphyllin continus 400mg tablets (napp pharmaceuticals ltd)
5516 salamol 100micrograms/dose easi-breathe inhaler (teva uk ltd)
5521 beclometasone 200micrograms/dose dry powder inhaler
5522 beclometasone 100micrograms/dose dry powder inhaler
5551 flixotide 0.5mg/2ml nebules (glaxosmithkline uk ltd)
5558 salmeterol 50micrograms with fluticasone 500micrograms cfc free inhaler
5580 flixotide accuhaler 50 50microgram/inhalation inhalation powder (allen & hanburys ltd)
5584 oxygen cylinders size pd (boc ltd)
5683 flixotide 250micrograms/dose evohaler (glaxosmithkline uk ltd)
5718 flixotide 125micrograms/dose evohaler (glaxosmithkline uk ltd)
5740 airomir 100micrograms/dose autohaler (teva uk ltd)
5753 salbutamol 400micrograms disc
5773 oxygen bp size dd gas 460 litres
5780 oxygen cylinders size af (boc ltd)
5804 beclometasone 250micrograms/dose dry powder inhaler
5822 fluticasone 250micrograms/dose inhaler cfc free
5837 salamol steri-neb 5mg/2.5ml nebuliser liquid (numark management ltd)
5864 salmeterol 25micrograms with fluticasone 250micrograms cfc free inhaler
5885 fluticasone 100micrograms/dose dry powder inhaler
5889 salamol 100microgram/inhalation inhalation powder (kent pharmaceuticals ltd)
5898 salamol steri-neb 2.5mg/2.5ml nebuliser liquid (numark management ltd)
5941 uniphyllin continus 300mg tablets (napp pharmaceuticals ltd)
5942 salmeterol 50micrograms with fluticasone 250micrograms cfc free inhaler
5975 fluticasone 125micrograms/dose inhaler cfc free
5976 oxygen bp size dd 460 litre inhalation gas (boc ltd)
5992 beclometasone 50micrograms/dose dry powder inhaler
6081 ipratropium bromide 20micrograms/dose breath actuated inhaler
6276 carbocisteine 250mg/5ml oral solution
6315 slo-phyllin 250mg capsules (merck serono ltd)
6325 symbicort 200/6 turbohaler (astrazeneca uk ltd)
6419 oxygen cylinders size f (boc ltd)
6420 oxygen bp size d gas 340 litres
6423 oxygen bp with integral headset 1360 litre inhalation gas (boc ltd)
6462 salbutamol 95micrograms/dose dry powder inhaler
6522 ipratropium bromide 20micrograms/dose inhaler cfc free
6526 formoterol 12microgram inhalation powder capsules with device

For peer review only

6556	oxygen cylinders size c (boc ltd)
6569	salmeterol 25micrograms with fluticasone 125micrograms cfc free inhaler
6616	salmeterol 25micrograms with fluticasone 50micrograms cfc free inhaler
6619	oxygen bp size cd gas 460 litres
6719	ipratropium bromide 500micrograms/2ml nebuliser liquid unit dose vials
6746	budesonide 400micrograms/dose / formoterol 12micrograms/dose dry powder inhaler
6758	ipratropium 250micrograms/1ml nebuliser liquid steri-neb unit dose vials (teva uk ltd)
6768	oxygen bp size cd 460 litre inhalation gas (boc ltd)
6772	ipratropium bromide 250micrograms/1ml nebuliser liquid unit dose vials
6780	symbicort 400/12 turbohaler (astrazeneca uk ltd)
6796	budesonide 200micrograms/dose / formoterol 6micrograms/dose dry powder inhaler
6802	mucodyne 375mg capsules (sanofi)
6848	oxygen cylinders size dd with integral headset 2 and 4litres/minute flow rate (boc ltd)
6920	mecysteine 100mg gastro-resistant tablets
6938	salmeterol 50micrograms with fluticasone 100micrograms dry powder inhaler
6976	oxygen bp with integral headset gas 430 litres
6988	aminophylline hydrate 100mg modified-release tablets
7013	symbicort 100/6 turbohaler (astrazeneca uk ltd)
7017	salbutamol 100micrograms/dose dry powder inhaler
7031	oxygen bp size e gas 680 litres
7042	oxygen cylinders size e (boc ltd)
7133	formoterol 12micrograms/dose dry powder inhaler
7192	bambuterol 10mg tablets
7239	oxygen cylinders size b10s (air products plc)
7268	serevent 25micrograms/dose evohaler (glaxosmithkline uk ltd)
7270	salmeterol 25micrograms/dose inhaler cfc free
7452	ventolin .25 mg inj
7584	prednisolone 4 mg tab
7602	fluticasone 50microgram disc
7638	fluticasone 250microgram disc
7653	beclometasone 400microgram inhalation powder capsules
7710	prednisolone 15 mg tab
7711	terbutaline 250micrograms/dose inhaler with spacer
7724	betamethasone valerate 100micrograms/actuation inhaler
7730	theo-dur 300mg tablets (astrazeneca uk ltd)
7731	theo-dur 200mg tablets (astrazeneca uk ltd)
7732	theophylline 300mg modified-release tablets
7733	theophylline 250mg modified-release tablets
7788	budesonide 100micrograms/dose dry powder inhaler
7832	choline theophyllinate 200mg tablets
7841	nuelin 125mg tablets (3m health care ltd)
7891	fluticasone 500microgram disc
7934	prednisone 30 mg tab
7935	maxivent 100microgram/inhalation inhalation powder (ashbourne pharmaceuticals ltd)
7948	fluticasone 250micrograms/dose dry powder inhaler
7953	terbutaline 1.5mg/5ml oral solution sugar free
7954	bricanyl 250micrograms/dose spacer inhaler (astrazeneca uk ltd)
7964	beclometasone 50micrograms/ml nebuliser suspension
7965	salbutamol 5mg/ml nebuliser liquid
8012	exirel 15mg capsule (3m health care ltd)
8056	aminophylline 100mg tablets
8057	aminophylline 100mg modified-release tablets
8111	becloforte vm 250microgram/actuation vm pack (allen & hanburys ltd)
8183	oxygen bp gas 640 litres

For peer review only

8251	pulmicort refil 50 mg inh
8252	pirbuterol 15mg capsule
8267	sodium cromoglicate 1mg/dose / salbutamol 100micrograms/dose inhaler
8333	ipratropium bromide 40microgram inhalation powder capsules
8339	fenoterol hydrobromide complete unit inh
8429	ventolin i/v 5 mg inj
8433	budesonide 100micrograms/actuation inhaler
8450	flixotide diskhaler-community pack 50 mcg
8504	exirel 15 mg tab
8505	oxygen bp gas 6400 litres
8522	terbutaline 7.5mg modified-release tablets
8572	rimiterol inhaler
8635	flixotide 50microgram disc (allen & hanburys ltd)
8636	ventolin s/r 8 mg spa
8676	terbutaline 10mg/ml nebuliser liquid
8757	oxygen bp gas 3200 litres
8806	phyllocontin continus 350mg tablet (napp pharmaceuticals ltd)
8955	theophylline 100 mg tab
9018	mucodyne 375mg capsule (aventis pharma)
9092	theophylline 350mg modified release tablets
9164	fluticasone 50micrograms/dose dry powder inhaler
9233	beclometasone 200microgram inhalation powder capsules
9270	ipratropium bromide with fenoterol hydrobromide 500micrograms + 1.25mg/4ml
9384	salbutamol 4mg modified-release capsules
9477	asmabec 100microgram/actuation spacehaler (celltech pharma europe ltd)
9571	beclometasone 250micrograms/actuation vortex inhaler
9577	asmabec 50 clickhaler (focus pharmaceuticals ltd)
9599	beclazone 50microgram/actuation inhalation powder (actavis uk ltd)
9642	mucodyne 250mg/5ml oral solution (aventis pharma)
9651	asmasal 100microgram/inhalation spacehaler (celltech pharma europe ltd)
9658	oxitropium bromide 100micrograms/dose breath actuated inhaler
9711	formoterol 6micrograms/dose dry powder inhaler
9727	prednisolone 50mg tablets
9805	salbutamol 5mg/50ml solution for infusion vials
9889	oxygen cylinders size g (boc ltd)
9895	oxygen bp size df with integral headset gas 1360 litres
9906	mucodyne 250mg/5ml syrup (sanofi)
9921	beclometasone 100micrograms/dose breath actuated inhaler cfc free
9943	visclair 100mg gastro-resistant tablets (ranbaxy (uk) ltd)
10043	oxygen composite cylinders size b10c with integral headset (air products plc)
10053	oxygen connection cubing 1.8m
10090	beclometasone 50micrograms/actuation extrafine particle cfc free inhaler
10218	budesonide 100micrograms/dose / formoterol 6micrograms/dose dry powder inhaler
10321	budesonide 400microgram inhalation powder capsules
10331	nuelin 60mg/5ml liquid (3m health care ltd)
10353	salbuvent rondo
10360	aerocrom inhaler (castlemead healthcare ltd)
10407	phyllocontin paediatric continus 100mg tablets (napp pharmaceuticals ltd)
10433	theophylline 60mg/5ml oral solution
10458	ventolin cr 4mg tablet (allen & hanburys ltd)
10561	aminophylline 250mg/ml injection
10723	theophylline 125mg/5ml syrup
10744	theophylline 80 mg eli
10808	mucodyne paediatric 125mg/5ml syrup (sanofi)
10825	terbutaline 5mg tablets

For peer review only

10831 biophylline 125mg/5ml oral solution (lorex synthelabo ltd)
10858 pulmadil auto inhalation powder (3m health care ltd)
10958 salbutamol .25 mg inj
10968 foradil 12microgram inhalation powder capsules with device (novartis pharmaceuticals uk ltd)
11046 ipratropium bromide with salbutamol 500micrograms + 2.5mg/2.5ml
11149 betnelan 500microgram tablets (focus pharmaceuticals ltd)
11198 beclometasons 50 micrograms/actuation vortex inhaler
11307 salbutamol 100micrograms/dose / beclometasone 50micrograms/dose inhaler
11410 fluticasone 500micrograms/dose / salmeterol 50micrograms/dose dry powder inhaler
11478 fluticasone 2mg/2ml nebuliser liquid unit dose vials
11497 beclometasone 400micrograms/dose dry powder inhaler
11588 fluticasone 125micrograms/dose / salmeterol 25micrograms/dose inhaler cfc free
11618 fluticasone 250micrograms/dose / salmeterol 25micrograms/dose inhaler cfc free
11659 oxygen bp size dd with integral headset gas 460 litres
11719 slo-phyllin 60mg capsules (merck serono ltd)
11732 beclometasone 50micrograms/dose breath actuated inhaler cfc free
11779 ipratropium bromide 40microgram inhalation powder capsules with device
11993 pro-vent 300mg capsule (wellcome medical division)
12042 ventolin cr 8mg tablet (allen & hanburys ltd)
12144 bambuterol 20mg tablets
12240 theophylline 300mg modified release capsules
12463 pirbuterol 15 mg tab
12479 aerolin inh auto refil 100 mcg aer
12486 bronchodil 500microgram/dose inhalation powder (viatris pharmaceuticals ltd)
12529 fabrol 200mg granules (novartis consumer health uk ltd)
12563 exirel inhalation powder (3m health care ltd)
12699 pecram 225mg modified-release tablet (novartis consumer health uk ltd)
12808 fenoterol 100micrograms/dose / ipratropium bromide 40micrograms/dose breath actuated inhaler
12822 salbutamol 2.5mg with ipratropium bromide 500micrograms/2.5ml unit dose nebuilser solution
12909 salbutamol 100micrograms/dose / ipratropium 20micrograms/dose inhaler
12994 fluticasone 50micrograms/dose / salmeterol 25micrograms/dose inhaler cfc free
13037 pulvinal beclometasone dipropionate 200micrograms/dose dry powder inhaler (chiesi ltd)
13038 pulvinal salbutamol 200micrograms/dose dry powder inhaler (chiesi ltd)
13040 fluticasone 250micrograms/dose / salmeterol 50micrograms/dose dry powder inhaler
13181 easyhaler salbutamol sulphate 100micrograms/dose dry powder inhaler (orion pharma (uk) ltd)
13206 oxygen bp with integral headset gas 300 litres
13273 fluticasone 100micrograms/dose / salmeterol 50micrograms/dose dry powder inhaler
13290 clenil modulite 100micrograms/dose inhaler (chiesi ltd)
13307 bricanyl 500microgram/ml injection (astrazeneca uk ltd)
13522 prednisolone 2 mg tab
13529 amnivent-225 sr tablets (ashbourne pharmaceuticals ltd)
13575 bambec 20mg tablets (astrazeneca uk ltd)
13615 prednisone 10 mg tab
13757 tropiovent steripoule 250microgram/ml nebuliser liquid (ashbourne pharmaceuticals ltd)
13815 beclazone 100microgram/actuation inhalation powder (actavis uk ltd)
13996 salamol 100microgram/inhalation inhalation powder (sandoz ltd)
14294 qvar 50micrograms/dose easi-breathe inhaler (teva uk ltd)
14306 formoterol 12micrograms/dose inhaler cfc free
14321 beclometasone 200micrograms/dose inhaler cfc free

For peer review only

14482 bricanyl 2.5 mg inj
14483 terbutaline 500micrograms/ml injection
14514 zithromax 200mg/5ml oral suspension (pfizer ltd)
14524 bdp 250microgram/actuation spacehaler (celltech pharma europe ltd)
14525 salbutamol 100micrograms/inhalation vortex inhaler
14527 bambec 10mg tablets (astrazeneca uk ltd)
14561 salbutamol 400microgram / beclometasone 200microgram inhalation powder capsules
14567 asmabec 250 clickhaler (focus pharmaceuticals ltd)
14590 asmabec 250microgram/actuation spacehaler (celltech pharma europe ltd)
14700 budesonide 400micrograms/actuation inhaler
14736 pulvinal beclometasone dipropionate 400micrograms/dose dry powder inhaler (chiesi ltd)
14739 norphyllin sr 225mg tablets (teva uk ltd)
14757 pulvinal beclometasone dipropionate 100micrograms/dose dry powder inhaler (chiesi ltd)
14991 aminophylline 250mg/10ml injection
15075 bronchodil 20mg tablet (viatris pharmaceuticals ltd)
15165 reproterol 500micrograms/dose inhaler
15214 oxygen bp size pd 300 litre inhalation gas (medigas ltd)
15281 oxygen cylinders size pa2 with integral headset (air products plc)
15284 slo-phyllin 125mg capsules (merck serono ltd)
15301 carbocisteine 125mg/5ml oral solution
15326 beclometasone 100micrograms/dose inhaler cfc free
15365 theophylline 10mg/5ml sf elixir
15409 theophylline 3 mg sol
15441 fenoterol hydrobromide .5 % sol
15613 salbutamol 500micrograms/1ml solution for injection ampoules
15706 beclometasone 100 micrograms/actuation vortex inhaler
15979 oxygen bp with integral headset 300 litre inhalation gas (boc ltd)
16054 budesonide 200micrograms/actuation breath actuated powder inhaler
16124 oxygen cylinders (medigas ltd)
16148 clenil modulite 250micrograms/dose inhaler (chiesi ltd)
16151 clenil modulite 200micrograms/dose inhaler (chiesi ltd)
16158 clenil modulite 50micrograms/dose inhaler (chiesi ltd)
16236 pirbuterol acetate inhaler
16305 flixotide 2mg/2ml nebules (glaxosmithkline uk ltd)
16523 oxygen cylinders size cd with integral headset 0-15litres/minute flow rate (boc ltd)
16577 easyhaler salbutamol sulphate 200micrograms/dose dry powder inhaler (orion pharma (uk) ltd)
16584 beclometasone 50micrograms/dose inhaler cfc free
16625 ventide rotacaps (glaxosmithkline uk ltd)
16724 prednisone 50 mg tab
16994 aminophylline hydrate 350mg modified-release tablets
17002 aminophylline hydrate 225mg modified-release tablets
17005 oxygen bp size cd with integral headset gas 460 litres
17140 aminophylline 200mg tablets
17185 ventolin 500micrograms/1ml solution for injection ampoules (glaxosmithkline uk ltd)
17465 fluticasone 500micrograms/2ml nebuliser liquid unit dose vials
17644 oxygen bp with integral headset gas 2122 litres
17654 easyhaler beclometasone 200micrograms/dose dry powder inhaler (orion pharma (uk) ltd)
17670 easyhaler budesonide 100micrograms/dose dry powder inhaler (orion pharma (uk) ltd)
17696 ventmax sr 4mg capsules (chiesi ltd)

For peer review only

17874 monovent 1.5mg/5ml oral solution (lagap)
17901 bricanyl nebule 2.5 ml
18140 respontin 500micrograms/2ml nebules (glaxosmithkline uk ltd)
18288 choline theophyllinate 100mg tablets
18299 fenoterol 1.25mg/4ml / ipratropium 500micrograms/4ml nebuliser liquid unit dose vials
18314 aerocrom syncroner with spacer (castlemead healthcare ltd)
18394 bdp 50microgram/actuation spacehaler (celltech pharma europe ltd)
18421 respontin nebules 250microgram/ml nebuliser liquid (glaxo wellcome uk ltd)
18456 salbutamol 200microgram / beclometasone 100microgram inhalation powder capsules
18484 ventide paediatric rotacaps (glaxosmithkline uk ltd)
18537 budesonide 200microgram inhalation powder capsules
18622 salbulin 2mg tablet (3m health care ltd)
18848 qvar 100micrograms/dose easi-breathe inhaler (teva uk ltd)
18937 sabidal sr 270 270 mg tab
18968 salbutamol 5mg/5ml solution for infusion ampoules
18988 choline theophyllinate 62.5mg/5ml oral solution
19031 bdp 100microgram/actuation spacehaler (celltech pharma europe ltd)
19121 beclometasone 100micrograms with salbutamol 200micrograms inhalation capsules
19376 beclometasone 200micrograms with salbutamol 400micrograms inhalation capsules
19389 asmabec 50microgram/actuation spacehaler (celltech pharma europe ltd)
19401 beclometasone 250micrograms/actuation inhaler and compact spacer
19563 becotide for nebuliser
19642 ventolin nebules
19649 ventolin rotahaler
19653 ventolin respirator
19726 ventolin s/r
19732 cobutolin inh
19735 uniphyllin continus
19736 becotide susp for nebulisation
19737 atrovent nebuliser solution (1ml vial)
19799 tulobuterol 2mg
19805 atrovent
20095 precortisyl forte 25mg tablet (aventis pharma)
20225 aminophylline 500 mg inj
20670 prednisolone e/c
20675 salbutamol rotahaler complete unit
20680 aerolin autohaler
20707 becotide 100
20720 atrovent forte
20763 becloforte
20781 salbutamol u.dose nebulising 2.5mg/2.5ml
20803 ipratropium bromide nebuliser solution
20812 pulmicort refill
20825 spacehaler bdp 250microgram/actuation spacehaler (celltech pharma europe ltd)
20838 salbuvent 2mg tablet (pharmacia ltd)
21005 beclometasone 250micrograms/dose inhaler cfc free
21102 salbutamol 2mg/5ml oral solution (lagap)
21330 oxygen composite cylinders size if2 with integral headset (medigas ltd)
21331 oxygen composite cylinders with integral headset (air products plc)
21402 oxygen cylinders size pd (air products plc)
21482 beclometasone 100micrograms/dose inhaler (generics (uk) ltd)
21769 lasma 300mg tablet (pharmax ltd)
21833 decortisyl 5mg tablet (roussel laboratories ltd)

For peer review only

21859 asmaven 100microgram inhalation powder (berk pharmaceuticals ltd)
22080 aminophylline 20 ml inj
22225 beclomethasone /salbutamol
22313 ventmax sr 8mg capsules (chiesi ltd)
22430 spacehaler salbutamol 100microgram/inhalation spacehaler (celltech pharma europe ltd)
22467 salbutamol respirator soln
22512 salbutamol inhaler
22550 duovent
22661 pirbuterol 10mg capsule
22663 respacal 2mg tablet (ucb pharma ltd)
22669 choline theophyllinate 270 mg tab
22790 reproterol 10mg/ml respirator solution
22828 carbocisteine 750mg/5ml forte oral solution
23269 maxivent 2.5mg/2.5ml nebuliser liquid unit dose steripoule vials (ashbourne pharmaceuticals ltd)
23337 oxygen cylinder f size 1280 litres
23512 precortisyl 5mg tablet (hoechst marion roussel)
23567 respontin 250micrograms/1ml nebules (glaxosmithkline uk ltd)
23572 aminophylline sr 225mg modified-release tablet (ivax pharmaceuticals uk ltd)
23675 pulmicort l.s. refil
23688 ventolin rotacaps
23709 ipratropium 500micrograms/2ml nebuliser liquid steri-neb unit dose vials (teva uk ltd)
23741 novolizer budesonide 200microgram/actuation pressurised inhalation (meda pharmaceuticals ltd)
23787 exirel 10mg capsule (3m health care ltd)
23905 oxygen cylinders size d (boc ltd)
23961 ipratropium bromide 250microgram/ml inhalation vapour (galen ltd)
24219 becotide rotacaps
24380 sodium cromoglicate 1mg/dose / salbutamol 100micrograms/dose inhaler with spacer
24418 biophylline 350mg tablet (lorex synthelabo ltd)
24456 carbocisteine 375mg tablets
24645 ventolin 5mg/5ml solution for infusion ampoules (glaxosmithkline uk ltd)
24660 betamethasone valerate
24674 biophylline 500mg tablet (lorex synthelabo ltd)
24716 prednisolone e/c
24898 spacehaler bdp 100microgram/actuation spacehaler (celltech pharma europe ltd)
25020 ipratropium bromide (forte)
25073 salbutamol
25093 theophylline s/r
25204 beclometasone 100micrograms/dose inhaler (a a h pharmaceuticals ltd)
25218 salbutamol cfc/free b/a
25272 precortisyl 1mg tablet (hoechst marion roussel)
25339 maxivent 5mg/2.5ml nebuliser liquid unit dose steripoule vials (ashbourne pharmaceuticals ltd)
25784 atimos modulite 12micrograms/dose inhaler (chiesi ltd)
25820 bronchodil 10mg/5ml oral solution (viatris pharmaceuticals ltd)
25821 exirel 7.5mg/5ml oral solution (3m health care ltd)
25829 pirbuterol 7.5mg/5ml oral solution
26063 beclometasone 100micrograms/dose inhaler (teva uk ltd)
26079 uniphyllin paediatric continus
26420 exirel 10 mg tab
26525 ventolin
26616 ipratropium bromide with fenoterol hydrobromide 0micrograms + 100micrograms/actuation

For peer review only

26665 pulmicort complete
26716 airomir autohaler cfc free b/a
26829 brelomax 2mg tablet (abbott laboratories ltd)
26873 cobutolin 2mg tablet (actavis uk ltd)
27040 phyllocontin continus
27188 easyhaler budesonide 200micrograms/dose dry powder inhaler (orion pharma (uk) ltd)
27301 bromhexine hcl 8mg tablets
27340 salbuvent 0.5mg/ml injection (pharmacia ltd)
27505 ipratropium bromide with fenoterol hydrobromide 40micrograms + 100micrograms/actuation
27525 becotide 50
27558 choledyl
27573 ventolin
27583 pulmicort
27679 beclometasone 100microgram/actuation pressurised inhalation (approved prescription services ltd)
27842 aminophylline 2 ml inj
27889 prednisolone
27915 fluticasone prop disk refill
27959 prednisolone
27962 deltastab 1mg tablet (waymade healthcare plc)
28073 beclometasone 250microgram/actuation pressurised inhalation (approved prescription services ltd)
28241 aminophylline 250mg/10ml solution for injection minijet pre-filled syringes (ucb pharma ltd)
28376 prednisolone 2.5mg gastro-resistant tablet (biorex laboratories ltd)
28508 salbutamol 100microgram/inhalation inhalation powder (ivax pharmaceuticals uk ltd)
28577 ventolin 50microgram/ml injection (allen & hanburys ltd)
28640 beclometasone 100microgram/actuation inhalation powder (actavis uk ltd)
28761 spacehaler bdp 50microgram/actuation spacehaler (celltech pharma europe ltd)
28859 deltastab 5mg tablet (waymade healthcare plc)
28881 salbutamol 2mg/5ml oral solution sugar free (a a h pharmaceuticals ltd)
29267 salbuvent 4mg tablet (pharmacia ltd)
29273 aminophylline 225mg modified-release tablet (hillcross pharmaceuticals ltd)
29325 beclometasone 250micrograms/dose inhaler (generics (uk) ltd)
30118 salbutamol 100micrograms/dose inhaler cfc free (teva uk ltd)
30204 salbutamol 200micrograms inahalation capsules
30210 beclometasone 250micrograms/dose inhaler (teva uk ltd)
30212 salbutamol cyclohaler
30229 ipratropium bromide 250microgram/ml nebuliser liquid (galen ltd)
30230 salbutamol 100micrograms/actuation breath actuated inhaler
30238 beclometasone 50microgram/actuation pressurised inhalation (approved prescription services ltd)
30240 aerolin autohaler 100microgram/actuation pressurised inhalation (3m health care ltd)
30390 deltastab 2 mg tab
30596 aminophylline 225mg modified-release tablet (actavis uk ltd)
30649 easyhaler budesonide 400micrograms/dose dry powder inhaler (orion pharma (uk) ltd)
30971 decortisyl 25 mg tab
31082 salbuvent 5mg/ml respirator solution (pharmacia ltd)
31290 salbulin cfc free
31327 prednisolone steaglate 6.65mg tablet
31758 uniphyllin continus
31774 beclometasone 50micrograms/dose inhaler (generics (uk) ltd)

For peer review only

31845	salapin 2mg/5ml syrup (pinewood healthcare)
31933	salbutamol 100micrograms/dose inhaler (a a h pharmaceuticals ltd)
32050	salbutamol 400 cyclocaps (teva uk ltd)
32102	salbutamol 4mg tablets (a a h pharmaceuticals ltd)
32461	choline theophyllinate 90 mg tab
32812	numotac 10mg tablet (3m health care ltd)
32874	beclometasone 50microgram/actuation inhalation powder (actavis uk ltd)
32893	theophylline 100mg/lysine 74mg mg tab
33089	salbutamol 100micrograms/dose inhaler (kent pharmaceuticals ltd)
33258	beclometasone 250micrograms/dose inhaler (a a h pharmaceuticals ltd)
33373	salbutamol 200 cyclocaps (teva uk ltd)
33588	salbutamol 100micrograms/dose inhaler (generics (uk) ltd)
33691	prednisolone 5mg gastro-resistant tablet (biorex laboratories ltd)
33817	salbutamol 100micrograms/dose inhaler cfc free (actavis uk ltd)
33849	beclometasone 100microgram/actuation inhalation powder (neo laboratories ltd)
33888	azithromycin 250mg tablets
33988	prednisolone 5mg tablet (co-pharma ltd)
33990	prednisolone 5mg tablet (ivax pharmaceuticals uk ltd)
34018	salbutamol 5mg/2.5ml nebuliser liquid (galen ltd)
34029	salbutamol 400micrograms inahalation capsules
34109	prednisolone 5 mg gastro-resistant tablet
34134	aerolin 400 100microgram/actuation inhalation powder (3m health care ltd)
34162	salbutamol 2.5mg/2.5ml nebuliser liquid (galen ltd)
34310	salbutamol 100micrograms/dose inhaler cfc free (a a h pharmaceuticals ltd)
34311	salbutamol 100microgram/inhalation inhalation powder (berk pharmaceuticals ltd)
34315	beclometasone 250microgram/actuation inhalation powder (actavis uk ltd)
34428	beclometasone 50microgram/actuation inhalation powder (neo laboratories ltd)
34618	salbutamol 2mg tablets (actavis uk ltd)
34619	salbutamol 100microgram/inhalation inhalation powder (kent pharmaceuticals ltd)
34631	prednisolone 1mg tablet (co-pharma ltd)
34702	salbutamol 100microgram/inhalation inhalation powder (c p pharmaceuticals ltd)
34739	beclometasone 50micrograms/dose inhaler (teva uk ltd)
34794	beclometasone 200micrograms/dose inhaler (a a h pharmaceuticals ltd)
34859	beclometasone 250microgram/actuation inhalation powder (neo laboratories ltd)
34914	prednisolone 1mg tablet (celltech pharma europe ltd)
34919	beclometasone 50micrograms/dose inhaler (a a h pharmaceuticals ltd)
34938	salbutamol 4mg tablets (actavis uk ltd)
35011	tiotropium bromide 18microgram inhalation powder capsules
35014	tiotropium bromide 18microgram inhalation powder capsules with device
35015	erdosteine 300mg capsules
35071	becodisks 200microgram (glaxosmithkline uk ltd)
35106	becodisks 100microgram with diskhaler (glaxosmithkline uk ltd)
35107	beclometasone 400microgram inhalation powder blisters with device
35113	beclometasone 200microgram inhalation powder blisters
35118	becodisks 400microgram with diskhaler (glaxosmithkline uk ltd)
35165	serevent 50microgram disks with diskhaler (glaxosmithkline uk ltd)
35178	erdotin 300mg capsules (galen ltd)
35225	flixotide 100microgram disks with diskhaler (glaxosmithkline uk ltd)
35288	beclometasone 400microgram inhalation powder blisters
35293	beclometasone 200microgram inhalation powder blisters with device
35299	becodisks 400microgram (glaxosmithkline uk ltd)
35374	flixotide 500microgram disks (glaxosmithkline uk ltd)
35392	flixotide 500microgram disks with diskhaler (glaxosmithkline uk ltd)
35408	becodisks 100microgram (glaxosmithkline uk ltd)
35430	becodisks 200microgram with diskhaler (glaxosmithkline uk ltd)

For peer review only

35461 flixotide 250microgram disks with diskhaler (glaxosmithkline uk ltd)
35503 salmeterol 50microgram inhalation powder blisters
35510 budesonide 200micrograms/dose dry powder inhalation cartridge with device
35522 bricanyl 500micrograms/1ml solution for injection ampoules (astrazeneca uk ltd)
35542 salmeterol 50microgram inhalation powder blisters with device
35557 ipramol nebuliser solution 2.5ml steri-neb unit dose vials (teva uk ltd)
35566 oxygen bp with set gas 1360 litres
35580 beclometasone 100microgram inhalation powder blisters with device
35602 budesonide 200micrograms/dose dry powder inhalation cartridge
35611 flixotide 250microgram disks (glaxosmithkline uk ltd)
35631 budelin novolizer 200micrograms/dose inhalation powder (meda pharmaceuticals ltd)
35638 fluticasone 100microgram inhalation powder blisters with device
35652 beclometasone 100microgram inhalation powder blisters
35700 fluticasone 500microgram inhalation powder blisters with device
35724 budelin novolizer 200micrograms/dose inhalation powder refill (meda pharmaceuticals ltd)
35725 formoterol easyhaler 12micrograms/dose dry powder inhaler (orion pharma (uk) ltd)
35744 bricanyl 2.5mg/5ml solution for injection ampoules (astrazeneca uk ltd)
35772 fluticasone 100microgram inhalation powder blisters
35825 serevent 50microgram disks (glaxosmithkline uk ltd)
35861 terbutaline 2.5mg/5ml solution for injection ampoules
35862 terbutaline 500micrograms/1ml solution for injection ampoules
35905 fluticasone 250microgram inhalation powder blisters
35986 flixotide 50microgram disks (glaxosmithkline uk ltd)
36021 fluticasone 50microgram inhalation powder blisters with device
36090 flixotide 100microgram disks (glaxosmithkline uk ltd)
36290 flixotide 50microgram disks with diskhaler (glaxosmithkline uk ltd)
36401 fluticasone 250microgram inhalation powder blisters with device
36462 fluticasone 500microgram inhalation powder blisters
36677 reproterol 10mg/5ml oral solution
36864 tiotropium bromide 2.5micrograms/dose solution for inhalation cartridge with device cfc free
37432 fostair 100micrograms/dose/6micrograms/dose inhaler (chiesi ltd)
37447 fluticasone 50microgram inhalation powder blisters
37470 beclometasone 100micrograms/dose / formoterol 6micrograms/dose inhaler cfc free
37612 terbutaline 5mg/2ml nebuliser liquid unit dose vials (galen ltd)
37791 ipratropium bromide 250microgram/ml
38079 salbutamol 100micrograms/dose dry powder inhalation cartridge with device
38097 salbutamol cyclocaps 200microgram inhalation powder (dupont pharmaceuticals ltd)
38120 theophylline 500mg modified release tablets
38136 salbulin novolizer 100micrograms/dose inhalation powder (meda pharmaceuticals ltd)
38214 salbutamol 100micrograms/dose dry powder inhalation cartridge
38226 salbulin novolizer 100micrograms/dose inhalation powder refill (meda pharmaceuticals ltd)
38407 prednisolone 20mg tablet
38416 salbutamol cyclocaps 400microgram inhalation powder (dupont pharmaceuticals ltd)
38419 terbutaline 1.5mg/5ml oral solution sugar free (a a h pharmaceuticals ltd)
39040 phyllocontin forte continus 350mg tablets (napp pharmaceuticals ltd)
39099 pulmicort 100micrograms/dose inhaler cfc free (astrazeneca uk ltd)
39102 budesonide 100micrograms/dose inhaler cfc free
39200 aerobec forte 250 autohaler (meda pharmaceuticals ltd)
39879 budesonide 200micrograms/dose inhaler cfc free
40057 pulmicort 200micrograms/dose inhaler cfc free (astrazeneca uk ltd)
40177 ipratropium bromide 250microgram/ml nebuliser liquid (hillcross pharmaceuticals ltd)
40218 azithromycin 500mg tablets (teva uk ltd)

For peer review only

40599 salbutamol 5mg/2.5ml nebuliser liquid unit dose steripoule vials (galen ltd)
40637 ipratropium 250micrograms/1ml nebuliser liquid unit dose steripoule vials (galen ltd)
40655 salbuvent 100microgram/actuation inhalation powder (pharmacia ltd)
40709 salbutamol 2.5mg/2.5ml nebuliser liquid unit dose vials (a a h pharmaceuticals ltd)
40832 ipratropium 500micrograms/2ml nebuliser liquid unit dose steripoule vials (galen ltd)
41269 beclometasone 400 cyclocaps (teva uk ltd)
41412 beclometasone 400micrograms/actuation inhaler
41548 salbutamol 2mg tablets (approved prescription services ltd)
41549 salbutamol 2mg tablet (c p pharmaceuticals ltd)
41691 salbutamol 2mg/5ml oral solution sugar free (sandoz ltd)
41832 monovent 1.5mg/5ml syrup (sandoz ltd)
42103 tulobuterol 1mg/5ml sugar free syrup
42279 salbutamol 2.5mg/2.5ml nebuliser liquid unit dose steripoule vials (galen ltd)
42497 salbutamol 8mg tablet
42511 aminophylline 25mg/ml injection (celltech pharma europe ltd)
42830 ventolin 100micrograms/dose evohaler (glaxosmithkline uk ltd)
42858 ventolin 200micrograms/dose accuhaler (glaxosmithkline uk ltd)
42867 terbutaline 1.5mg/5ml oral solution (sandoz ltd)
42886 bricanyl 500micrograms/dose turbohaler (astrazeneca uk ltd)
42910 aminophylline 250mg/10ml solution for injection ampoules (martindale pharmaceuticals ltd)
42928 flixotide 100micrograms/dose accuhaler (glaxosmithkline uk ltd)
42985 flixotide 50micrograms/dose accuhaler (glaxosmithkline uk ltd)
42994 flixotide 250micrograms/dose accuhaler (glaxosmithkline uk ltd)
43046 salipraneb 0.5mg/2.5mg nebuliser solution 2.5ml ampoules (arrow generics ltd)
43074 flixotide 500micrograms/dose accuhaler (glaxosmithkline uk ltd)
43085 bricanyl 5mg/2ml respules (astrazeneca uk ltd)
43400 clamelle 500mg tablets (actavis uk ltd)
43544 prednisone 5mg tablet (knoll ltd)
43738 indacaterol 150microgram inhalation powder capsules with device
43794 nebusal 7% inhalation solution 4ml ampoules (forest laboratories uk ltd)
43870 sodium chloride 7% inhalation solution 4ml ampoules
43893 onbrez breezhaler 150microgram inhalation powder capsules with device (novartis pharmaceuticals uk ltd)
44064 onbrez breezhaler 300microgram inhalation powder capsules with device (novartis pharmaceuticals uk ltd)
44173 roflumilast 500microgram tablets
44380 prednisone 1mg modified-release tablets
44431 daxas 500microgram tablets (takeda uk ltd)
44713 salbutamol 100microgram/inhalation inhalation powder (celltech pharma europe ltd)
45302 prednisolone 5mg tablet (biorex laboratories ltd)
45610 indacaterol 300microgram inhalation powder capsules with device
45863 salbutamol 5mg/2.5ml nebuliser liquid (generics (uk) ltd)
46157 beclometasone 200 cyclocaps (teva uk ltd)
46551 salbutamol 100microgram/inhalation inhalation powder (neo laboratories ltd)
46695 azithromycin 500mg tablet (hillcross pharmaceuticals ltd)
49016 sodium chloride 7% inhalation solution 4ml vials
49045 nebusal 7% inhalation solution 4ml vials (forest laboratories uk ltd)
49530 azithromycin 200mg/5ml oral suspension (sandoz ltd)
50508 mucodyne 250mg/5ml syrup (sigma pharmaceuticals plc)
52029 zithromax 250mg capsules (mawdsley-brooks & company ltd)
53303 carbocisteine 375mg capsules (arrow generics ltd)

For peer review only

Spirometry codes

medcode	readterm
6118	spirometry
10336	spirometry reversibility
10337	spirometry screening
10420	spirometry reversibility negative
10492	spirometry reversibility positive
13683	referral for spirometry
14453	forced expiratory volume - fev
19428	chronic obstructive pulmonary disease excluded by spirometry
26241	spirometry indicated
29015	spirometry
45993	incentive spirometry
102522	post bronchodilator spirometry
6091	Percent predicted FEV1
8512	FEV1/FVC percent
10320	Forced expired volume in 1 second
11078	FEV1/FVC > 70% of predicted
14455	FEV1/FVC ratio normal
14456	FEV1/FVC ratio
19830	FEV1 after bronchodilation
19832	FEV1/FVC ratio after bronchodilator
23237	FEV1 before bronchodilation
23285	FEV1/FVC ratio abnormal
25083	FEV1/FVC < 70% of predicted
27141	FEV1/FVC ratio before bronchodilator
43040	FEV1 pre steroids
43041	FEV1 post steroids
58632	FEV1/FVC ratio pre steroids
58633	FEV1/FVC ratio post steroids
99777	Forced expired volume in 1 second reversibility
100391	Forced expired volume in 1 second percentage change
101079	Percentage predicted FEV1 after bronchodilation

Non-specific bronchitis codes / Frequent winter bronchitis

medcode	readterm
148	bronchitis unspecified
152	wheezy bronchitis
3243	chronic bronchitis
3480	bronchitis nos
4519	h/o: bronchitis
5798	chronic asthmatic bronchitis
5909	chronic wheezy bronchitis
7092	recurrent wheezy bronchitis
11150	mucopurulent chronic bronchitis
13247	fh: bronchitis
13262	fh: bronchitis/coad
14798	emphysematous bronchitis
15157	chronic bronchitis nos
15626	chronic catarrhal bronchitis
17359	chest infection - unspecified bronchitis
24248	mixed simple and mucopurulent chronic bronchitis
25603	simple chronic bronchitis
27819	obstructive chronic bronchitis

For peer review only

37959	fetid chronic bronchitis
40159	purulent chronic bronchitis
44525	obstructive chronic bronchitis nos
61118	simple chronic bronchitis nos
61513	mucopurulent chronic bronchitis nos
62913	[v]screening for chronic bronchitis or emphysema
66043	other chronic bronchitis
68066	other chronic bronchitis nos
202167	INFLUENZAL BRONCHITIS
207165	Acute streptococcal bronchitis
207166	Acute viral bronchitis unspecified
216109	Acute membranous bronchitis
216111	Acute bronchitis due to mycoplasma pneumoniae
216112	Acute bronchitis NOS
216141	Chest infection - unspecified bronchopneumonia
220234	BRONCHITIS FIBRINOUS ACUTE
225216	Acute pseudomembranous bronchitis
225241	Bronchitis NOS
234340	Acute bronchitis due to rhinovirus
239395	H/O: bronchitis
243349	Acute bronchitis and bronchiolitis
243350	Acute pneumococcal bronchitis
243351	Acute bronchitis due to echovirus
243389	Bronchitis unspecified
261710	Acute wheezy bronchitis
261711	Acute purulent bronchitis
261712	Acute croupous bronchitis
261713	Acute bronchitis due to respiratory syncytial virus
271007	Acute bronchitis due to coxsackievirus
280054	Acute fibrinous bronchitis
280055	Acute bronchitis due to parainfluenza virus
280082	Chest infection - unspecified bronchitis
289164	Acute bronchitis
289165	Acute neisseria catarrhalis bronchitis
289166	Subacute bronchitis unspecified
289233	[X]Acute bronchitis due to other specified organisms
293231	BRONCHITIS SUBACUTE
298435	Acute haemophilus influenzae bronchitis
298436	Acute bacterial bronchitis unspecified
298440	Acute bronchitis or bronchiolitis NOS
303952	BRONCHITIS ACUTE
303954	VIRAL BRONCHITIS
303963	BRONCHOPNEUMONIA
306525	BRONCHITIS PURULENT
306528	CATARRHAL BRONCHITIS
308788	Acute bronchitis

Respiratory symptom codes

Wheeze

medcode	readterm
152	wheezy bronchitis
173	wheezing
2210	[d]wheezing

For peer review only

2891	wheezing symptom
4836	nocturnal cough / wheeze
5861	o/e - expiratory wheeze
5909	chronic wheezy bronchitis
5978	acute wheezy bronchitis
7092	recurrent wheezy bronchitis
42317	increasing exercise wheeze
100954	[d]mild wheeze
101037	[d]moderate wheeze
101073	[d]severe wheeze
101421	[d]very severe wheeze
102480	on examination - inspiratory wheeze

Chronic cough

medcode	readterm
92	cough
292	chesty cough
1025	bronchial cough
1160	[d]cough
1234	productive cough nos
1273	c/o - cough
1612	chronic cough
3068	night cough present
3628	persistent cough
3645	coughing up phlegm
4070	morning cough
4836	nocturnal cough / wheeze
4931	dry cough
7706	productive cough -clear sputum
7707	cough symptom nos
7708	productive cough-yellow sputum
7773	productive cough -green sputum
16717	smokers' cough
22318	difficulty in coughing up sputum
29318	evening cough
43795	unexplained cough
60903	cough aggravates symptom
100333	cough on exercise

Exertional breathlessness

medcode	readterm
735	[d]breathlessness
1429	breathlessness
3092	[d]dyspnoea
5175	breathlessness symptom
5896	dyspnoea - symptom
6326	breathless - moderate exertion
7000	o/e - dyspnoea
7932	breathless - mild exertion
19426	mrc breathlessness scale: grade 3
19427	mrc breathlessness scale: grade 2
19429	mrc breathlessness scale: grade 5

For peer review only

19430	mrc breathlessness scale: grade 4
19432	mrc breathlessness scale: grade 1
21801	breathlessness nos
24889	breathless - strenuous exertion
31143	breathless - at rest
42287	borg breathlessness score: 6 severe (+)
53771	dyspnoea on exertion
57193	borg breathlessness score: 3 moderate
57759	borg breathlessness score: 2 slight
59860	borg breathlessness score: 4 somewhat severe
64049	borg breathlessness score: 5 severe
67566	borg breathlessness score: 9 very, very sev (almost maximal)
68707	borg breathlessness score: 1 very slight
70061	borg breathlessness score: 7 very severe
70818	borg breathlessness score: 0.5 very, very slight
72334	borg breathlessness score: 8 very severe (+)
101843	borg breathlessness score: 10 maximal
741	[D]Shortness of breath
2563	[D]Respiratory distress
2575	Short of breath on exertion
2931	Difficulty breathing
4822	Shortness of breath
5349	Shortness of breath symptom
9297	[D]Respiratory insufficiency
22094	Short of breath dressing/undressing
40813	Unable to complete a sentence in one breath

Sputum codes

medcode	readterm
292	chesty cough
1025	bronchial cough
1234	productive cough nos
3645	coughing up phlegm
7706	productive cough -clear sputum
7708	productive cough-yellow sputum
7773	productive cough -green sputum
22318	difficulty in coughing up sputum
1251	[d]abnormal sputum
3727	sputum sent for c/s
8287	sputum sample obtained
8760	[d]positive culture findings in sputum
9807	sputum - symptom
14804	sputum appears infected
15430	[d]sputum abnormal - colour
16026	sputum examination: abnormal
18964	sputum clearance
20086	[d]sputum abnormal - amount
22318	difficulty in coughing up sputum
23582	[d]abnormal sputum nos
24181	sputum: mucopurulent
29294	sputum appears normal
30754	yellow sputum
30904	sputum sent for examination
31417	sputum - not infected
35577	sputum examination - general

For peer review only

36515 [d]abnormal sputum - tenacious
36880 green sputum
40201 sputum microscopy: nad
40202 mucoid sputum - o/e
42573 clear sputum
43270 sputum evidence of infection
43272 sputum examination nos
44214 [d]sputum abnormal - odour
49029 sputum gen. exam. nos
49694 sputum: organism on gram stain
52806 sputum: frothy/watery
54177 sputum: excessive - mucoid
61079 sputum inspection nos
91680 expectoration of induced sputum from respiratory tract
100484 volume of sputum
100524 moderate sputum
100629 white sputum
100647 copious sputum
100931 brown sputum
101782 profuse sputum
103209 grey sputum

QOF codes

H3... Chronic obstructive pulmonary disease
H31.. Chronic bronchitis
H310. Simple chronic bronchitis
H3100 Chronic catarrhal bronchitis
H310z Simple chronic bronchitis NOS
H311. Mucopurulent chronic bronchitis
H3110 Purulent chronic bronchitis
H3111 Fetid chronic bronchitis
H311z Mucopurulent chronic bronchitis NOS
H312. Obstructive chronic bronchitis
H3120 Chronic asthmatic bronchitis
H3121 Emphysematous bronchitis
H3123 Bronchiolitis obliterans
H312z Obstructive chronic bronchitis NOS
H313. Mixed simple and mucopurulent chronic bronchitis
H31y. Other chronic bronchitis
H31y1 Chronic tracheobronchitis
H31yz Other chronic bronchitis NOS
H31z. Chronic bronchitis NOS
H32.. Emphysema
H320. Chronic bullous emphysema
H3200 Segmental bullous emphysema
H3201 Zonal bullous emphysema
H3202 Giant bullous emphysema
H3203 Bullous emphysema with collapse
H320z Chronic bullous emphysema NOS
H321. Panlobular emphysema
H322. Centrilobular emphysema
H32y. Other emphysema
H32y0 Acute vesicular emphysema

For peer review only

H32y1 Atrophic (senile) emphysema
H32y2 MacLeod's unilateral emphysema
H32yz Other emphysema NOS
H32z. Emphysema NOS
H36.. Mild chronic obstructive pulmonary disease
H37.. Moderate chronic obstructive pulmonary disease
H38.. Severe chronic obstructive pulmonary disease
H39.. Very severe chronic obstructive pulmonary disease
H3A.. End stage chronic obstructive airways disease (v23)
H3y.. Other specified chronic obstructive airways disease
~~H3y0. Chronic obstructive pulmonary disease with acute lower respiratory infection~~ (v23)
~~H3y1. Chronic obstructive pulmonary disease with acute exacerbation, unspecified~~ (v23)
H3z.. Chronic obstructive airways disease NOS

GP Questionnaire

1. Do you think this patient has COPD? Yes / No / Uncertain
2. What was the diagnosis of COPD based on? (please circle all that apply)
   Smoking history symptoms spirometry other (please describe)
3. Has a respiratory physician seen the patient and confirmed a diagnosis of COPD?
4. Does the patient have any other respiratory condition?
   If yes, than what?

Table S1: The PPV and proportion of patients diagnosed with COPD who also had cardiovascular disease or asthma

Algorithm	Number with CVDx	Number confirmed COPD	PPV and 95% CI	Number with asthma	Number confirmed COPD	PPV and 95% CI
**COPD Code + spirometry + COPD medication**	20	17	85.0, 59.6-95.6	18	14	72.2, 45.3-89.1
**COPD Code + spirometry**	24	20	83.3, 61.1-94.1	20	14	70.0, 45.0-87.0
**COPD Code + COPD medication**	18	16	88.9, 61.1-97.6	16	9	56.3, 29.8-79.6
**COPD Code only**	18	16	88.9, 61.1-97.6	15	12	80.0, 48.8-94.4
**Bronchitis + COPD medication**	21	8	38.1, 19.1-61.7	27	4	14.8, 5.3-35.1
**Bronchitis only**	19	8	42.1, 21.1-66.5	23	6	26.1, 11.4-49.1
**Symptoms + spirometry**	15	9	60.0, 31.8-82.9	19	4	21.1, 7.3-47.3
**Symptoms only**	9	2	22.2, 3.9-67.0	23	2	8.7, 1.9-31.4

For peer review only

Table S2: The PPV and proportion of patients diagnosed with COPD who were current smokers

Algorithm	Number current smokers	Number confirmed COPD	PPV and 95% CI
**COPD Code + spirometry + COPD medication**	49	44	89.9, 77.1-95.8
**COPD Code + spirometry**	51	39	76.5, 62.5-86.4
**COPD Code + COPD medication**	55	46	83.6, 71.0-91.4
**COPD Code only**	47	41	87.2, 73.8-94.3
**Bronchitis + COPD medication**	67	34	50.7, 38.7-62.7
**Bronchitis only**	64	19	29.7, 19.6-42.3
**Symptoms + spirometry**	50	20	40.0, 27.1-54.5
**Symptoms only**	58	6	10.3, 4.6-21.7

Table S3: The PPV and proportion of patients diagnosed with COPD by COPD severity

Algorithm	GOLD 1 Number confirmed, (PPV and 95% CI)	GOLD 2 Number confirmed, (PPV and 95% CI)	GOLD 3 Number confirmed, (PPV and 95% CI)	GOLD 4 Number confirmed, (PPV and 95% CI)
**COPD Code + spirometry + COPD medication**	9, (69.2, 36.5-89.8)	34, (87.2, 71.8-94.8)	22, (100)	6, (100)
**COPD Code + spirometry**	8, (66.7, 32.9-89.1)	35, (83.3, 68.2-92.1)	20, (100)	3, (75, 4.1-99.5)
**COPD Code + COPD medication**	14, (82.4, 53.7-94.9)	32 (84.2, 68.1-93.0)	20, (95.2, 69.1-99.4)	6, (100)
**COPD Code only**	14, (82.4, 53.7-94.9)	37, (86.0, 71.5-93.8)	14, (93.3, 58.4-99.3)	3, (100)
**Bronchitis + COPD medication**	7, (36.8, 17.3-62.0)	20, (76.9, 55.6-89.9)	11, (91.7, 49.9-99.2)	2, (100)
**Bronchitis only**	3, (20.0, 5.6-51.2)	7, (63.6, 28.8-83.3)	10 (100)	1, (100)
**Symptoms + spirometry**	4, (57.1, 15.0-90.9)	19, (90.5, 66.0-97.9)	10 (90.9, 46.3-99.1)	1, (100)
**Symptoms only**	2, (40, 3.8-91.9)	6, (100)	0	0

For peer review only

Table S4: The PPV and proportion of patients diagnosed with COPD by timing of diagnosis

**Algorithm**	**2004 to end 2008**			**Post 2008**		
	**Number in algorithm**	**Number confirmed COPD**	**PPV and 95% CI**	**Number in algorithm**	**Number confirmed COPD**	**PPV and 95% CI**
**COPD Code + spirometry + COPD medication**	32	28	87.5, 69.8-95.5	53	48	90.6, 78.7-96.1
**COPD Code + spirometry**	14	11	78.6, 46.0-94.0	66	56	84.8, 73.7-91.8
**COPD Code + COPD medication**	27	23	85.2, 64.9-94.7	61	54	88.5, 77.4-94.5
**COPD Code only**	21	16	76.2, 51.7-90.5	68	61	89.7, 79.6-95.1
**Bronchitis + COPD medication**	94	42	44.7, 34.8-55.0	5	2	40.0, 3.8-91.9
**Bronchitis only**	83	22	26.5, 18.0-37.2	5	4	80.0, 11.1-99.2
**Symptoms + spirometry**	63	28	44.4, 32.4-57.1	22	9	40.9, 21.6-63.5
**Symptoms only**	64	10	15.6, 8.5-27.0	26	1	3.8, 0.47-25.4

For peer review only

STROBE Statement—checklist of items that should be included in reports of observational studies

	Item No	Recommendation
**Title and abstract** **Pg 1 and 2**	1	(*a*) Indicate the study's design with a commonly used term in the title or the abstract
		(*b*) Provide in the abstract an informative and balanced summary of what was done and what was found
**Introduction**		
Background/rationale **Pg 3**	2	Explain the scientific background and rationale for the investigation being reported
Objectives **Pg 4**	3	State specific objectives, including any prespecified hypotheses
**Methods**		
Study design **Pg 4**	4	Present key elements of study design early in the paper
Setting **Pg 4**	5	Describe the setting, locations, and relevant dates, including periods of recruitment, exposure, follow-up, and data collection
Participants **Pg 6**	6	(*a*) *Cohort study*—Give the eligibility criteria, and the sources and methods of selection of participants. Describe methods of follow-up<br>*Case-control study*—Give the eligibility criteria, and the sources and methods of case ascertainment and control selection. Give the rationale for the choice of cases and controls<br>*Cross-sectional study*—Give the eligibility criteria, and the sources and methods of selection of participants
		(*b*) *Cohort study*—For matched studies, give matching criteria and number of exposed and unexposed<br>*Case-control study*—For matched studies, give matching criteria and the number of controls per case
Variables **Pg 5,7**	7	Clearly define all outcomes, exposures, predictors, potential confounders, and effect modifiers. Give diagnostic criteria, if applicable
Data sources/ measurement **Pg 7**	8*	For each variable of interest, give sources of data and details of methods of assessment (measurement). Describe comparability of assessment methods if there is more than one group
Bias **Pg 6**	9	Describe any efforts to address potential sources of bias
Study size **Pg 8**	10	Explain how the study size was arrived at
Quantitative variables **Pg 7 and 8**	11	Explain how quantitative variables were handled in the analyses. If applicable, describe which groupings were chosen and why
Statistical methods **Pg 7 and 8**	12	(*a*) Describe all statistical methods, including those used to control for confounding
		(*b*) Describe any methods used to examine subgroups and interactions
		(*c*) Explain how missing data were addressed
		(*d*) *Cohort study*—If applicable, explain how loss to follow-up was addressed<br>*Case-control study*—If applicable, explain how matching of cases and controls was addressed<br>*Cross-sectional study*—If applicable, describe analytical methods taking account of sampling strategy

For peer review only

For peer review only - http://bmjopen.bmj.com/site/about/guidelines.xhtml

(*e*) Describe any sensitivity analyses

Continued on next page

For peer review only

For peer review only - http://bmjopen.bmj.com/site/about/guidelines.xhtml

**Results**		
Participants **Pg 9**	13*	(a) Report numbers of individuals at each stage of study—eg numbers potentially eligible, examined for eligibility, confirmed eligible, included in the study, completing follow-up, and analysed
		(b) Give reasons for non-participation at each stage
		(c) Consider use of a flow diagram
Descriptive data **Pg 9 and 10**	14*	(a) Give characteristics of study participants (eg demographic, clinical, social) and information on exposures and potential confounders
		(b) Indicate number of participants with missing data for each variable of interest
		(c) *Cohort study*—Summarise follow-up time (eg, average and total amount)
Outcome data **Pg 11**	15*	*Cohort study*—Report numbers of outcome events or summary measures over time
		Case-control study—Report numbers in each exposure category, or summary measures of exposure
		Cross-sectional study—Report numbers of outcome events or summary measures
Main results **Pg 10-12**	16	(*a*) Give unadjusted estimates and, if applicable, confounder-adjusted estimates and their precision (eg, 95% confidence interval). Make clear which confounders were adjusted for and why they were included
		(*b*) Report category boundaries when continuous variables were categorized
		(*c*) If relevant, consider translating estimates of relative risk into absolute risk for a meaningful time period
Other analyses **Supplementary material**	17	Report other analyses done—eg analyses of subgroups and interactions, and sensitivity analyses
**Discussion**		
Key results **Pg 12**	18	Summarise key results with reference to study objectives
Limitations **Pg 15**	19	Discuss limitations of the study, taking into account sources of potential bias or imprecision. Discuss both direction and magnitude of any potential bias
Interpretation **Pg 12-15**	20	Give a cautious overall interpretation of results considering objectives, limitations, multiplicity of analyses, results from similar studies, and other relevant evidence
Generalisability **Pg 15**	21	Discuss the generalisability (external validity) of the study results
**Other information**		
Funding **Pg 17**	22	Give the source of funding and the role of the funders for the present study and, if applicable, for the original study on which the present article is based

*Give information separately for cases and controls in case-control studies and, if applicable, for exposed and unexposed groups in cohort and cross-sectional studies.

Note: An Explanation and Elaboration article discusses each checklist item and gives methodological background and published examples of transparent reporting. The STROBE checklist is best used in conjunction with this article (freely available on the Web sites of PLoS Medicine at http://www.plosmedicine.org/, Annals of Internal Medicine at http://www.annals.org/, and Epidemiology at http://www.epidem.com/). Information on the STROBE Initiative is available at www.strobe-statement.org.

For peer review only

For peer review only - http://bmjopen.bmj.com/site/about/guidelines.xhtml

BMJ Open

Validation of Chronic Obstructive Pulmonary Disease (COPD) recording in the Clinical Practice Research Datalink (CPRD-GOLD)

Journal:	*BMJ Open*
Manuscript ID:	bmjopen-2014-005540.R2
Article Type:	Research
Date Submitted by the Author:	20-Jun-2014
Complete List of Authors:	Quint, Jennifer; London School of Hygeine and Tropical Medicine, Epidemiology<br>M�llerova, Hana; GlaxoSmithKline, Epidemiology Department<br>DiSantostefano, Rachael; GlaxoSmithKline, Worldwide Epidemiology<br>Forbes, Harriet; London school of Hygiene and Tropical Medicine, Epidemiology Department<br>Eaton, Susan; MHRA, Clincal Practice Research Datalink Group<br>Hurst, John; UCL Medical School,, Academic Unit of Respiratory Medicine<br>Davis, Kourtney<br>Smeeth, Liam; London School of Hygiene and Tropical Medicine, Epidemiology and Population Health
**Primary Subject Heading**:	Respiratory medicine
Secondary Subject Heading:	Epidemiology
Keywords:	RESPIRATORY MEDICINE (see Thoracic Medicine), THORACIC MEDICINE, Chronic airways disease < THORACIC MEDICINE, Epidemiology < THORACIC MEDICINE

SCHOLARONE™
Manuscripts

Validation of Chronic Obstructive Pulmonary Disease (COPD) recording in the Clinical Practice Research Datalink (CPRD-GOLD)

Jennifer K Quint[1], Hana Müllerova[2], Rachael L DiSantostefano[3], Harriet Forbes[1], Susan Eaton[4], John R Hurst[5], Kourtney Davis[2], Liam Smeeth[1].

[1]Department of Non-communicable disease Epidemiology, London School of Hygiene and Tropical Medicine, London, UK
[2] Respiratory Epidemiology, GlaxoSmithKline R&D, Uxbridge, UK
[3] Respiratory Epidemiology, GlaxoSmithKline R&D, RTP, NC USA
[4] Clinical Practice Research Datalink Group, Medicines and Healthcare products Regulatory Agency, London, UK
[5]UCL Respiratory Medicine, Royal Free Campus, University College London Medical School, London, UK

Corresponding author:
Dr. Jennifer K Quint
Department of Non-communicable disease Epidemiology
London School of Hygiene and Tropical Medicine
Keppel Street, London WC1E 7HT
Tel: 0207 927 2622
Email: **Jennifer.quint@lshtm.ac.uk**

Keywords: COPD, diagnosis of COPD, validation, spirometry, electronic health records

Word count: 3448

For peer review only

ABSTRACT

Objectives: The optimal method of identifying people with COPD from electronic primary care records is not known. We assessed the accuracy of different approaches using the Clinical Practice Research Datalink (CPRD), a UK electronic health record database.

Setting: Nine hundred and fifty one participants registered with a CPRD practice in the UK between 1st January 2004 and 31st December 2012. Individuals were selected for ≥1 of eight algorithms to identify people with COPD. General practitioners were sent a brief questionnaire and additional evidence to support a COPD diagnosis was requested. All information received was reviewed independently by two respiratory physicians whose opinion was taken as gold standard.

Primary outcome measure: The primary measure of accuracy was the positive predictive value (PPV); the proportion of people identified by each algorithm for whom COPD was confirmed.

Results: Nine hundred and fifty one questionnaires were sent and 738 (78%) returned. After quality control, 696 (73.2%) patients were included in the final analysis. All four algorithms including a specific COPD diagnostic code performed well. Using a diagnostic code alone the PPV was 86.5% (77.5% to 92.3%) while requiring a diagnosis plus spirometry plus specific medication the PPV was slightly higher at 89.4% (80.7% to 94.5%) but reduced case numbers by 10%. Algorithms without specific diagnostic codes had low PPVs (range 12.2% to 44.4%).

Conclusion: Patients with COPD can be accurately identified from UK primary care records using specific diagnostic codes. Requiring spirometry or COPD medications only marginally improved accuracy. The high accuracy applies since the introduction of an incentivised disease register for COPD as part of QOF (Quality and Outcomes Framework) in 2004.

For peer review only

Article Summary: This paper investigates how individuals with COPD can be accurately identified in electronic health records using specific COPD codes alone without additional information (i.e. presence of spirometry or respiratory medications). This information is essential for appropriate disease management, epidemiological research of COPD and other conditions, where COPD is an important co-morbidity.

Strengths and limitations of this study:

- We have shown that the presence of a specific COPD Read code alone is sufficient to identify patients with COPD from electronic health records. Minimal precision lost by not including spirometry and medications in the algorithm, allows an increase in the number of individuals who can potentially be included in a study by up to 10%.
- We were able to investigate both the accuracy of algorithms when identifying COPD patients within the CPRD, and accuracy of the actual GP diagnosis of COPD.
- The amount of missing data among the responding questionnaires was low, suggesting reasonable data quality.
- Although the overall response rate for this study was acceptable (77.6%), the proportion of questionnaires accompanied with additional evidence allowing for adjudication was lower.

‘

For peer review only

INTRODUCTION

Chronic obstructive pulmonary disease (COPD) represents an enormous health burden worldwide. Currently COPD is the 4th leading cause of death and is predicted to become the 3rd by 2020.[1] There are approximately 835,000 people diagnosed with COPD in the UK and an estimated 2,200,000 people remain undiagnosed.[2,3]

Electronic health records in the UK provide an excellent resource in which to study COPD as they offer a large cohort size, the presence of disease severity indicators and long-term follow-up information on a patient's integrated medical history. Although multiple studies have been undertaken to investigate various aspects of COPD over the last 10 years in several electronic health record databases, there is no standard definition used to identify COPD in large databases and codelists used to identify patients with COPD vary by author. Over ten years ago, the diagnosis of COPD was validated in the Clinical Practice Research Datalink (CPRD-GOLD, formerly GPRD) using OXMIS codes.[4] This coding system is now obsolete in CPRD and Read codes are used as standard.

There is no single diagnostic test for COPD. The diagnosis of COPD relies on clinical judgement based on a combination of history, physical examination and confirmation of the presence of airflow obstruction using spirometry.[5] When retrospectively evaluating the accuracy of a COPD diagnosis, commonly used COPD definitions may misclassify patients as having COPD. Using multiple diagnostic codes in combination with pharmacy may improve the accuracy of identification of COPD patients.[6] Further, over the past 10 years the definition of COPD in clinical practice has evolved leading to changes in how COPD is diagnosed and the diagnosis recorded. With the introduction of the Quality and Outcomes Framework (QOF) codes for COPD in 2004 in England and Wales there are more codes available to identify COPD then there were previously. QOF is a voluntary incentive scheme for primary care physicians (GPs) in the UK which contains indicators against which GP practices can score points and hence be rewarded for how well they care for patients. [7]

For peer review only

Evaluation of airflow limitation using spirometry is used as standard to confirm COPD diagnosis and severity of COPD is part of the annual COPD patient review.[3,8] However, even spirometry if incorrectly performed or interpreted can lead to misdiagnosis (both over and under diagnosis of COPD) in approximately 20% of cases. It is also recognised that some subgroups of individuals (e.g. women and individuals under 50 and over 80) are less likely to have spirometry measured.[8]

This study aimed to improve our ability to identify patients with COPD within electronic health records. We used the CPRD, a widely used collection of computerised medical records which is commonly used for clinical and research purposes. CPRD is comparable to other electronic health record databases used in the UK. Our objective was to test the accuracy of different definitions of COPD in the CPRD using Positive Predictive Value (PPV), by comparing the database records with additional information provided by General Practitioners (GPs). This work is important for epidemiological research in COPD and diseases where COPD is an important co-morbidity as well as for clinical practice.

For peer review only

METHODS

Dataset

CPRD is the world's largest validated computerized database of anonymized longitudinal medical records for primary care. [9,10] Data comprise approximately 14 million patients with around 5.4 million of these being currently alive and registered from 660 primary care practices spread throughout the UK. Records are derived from a widely used GP software system and contain complete prescribing and coded diagnostic and clinical information as well as information on tests requested, laboratory results and referrals made at or following on from each consultation. [11]

Codelists and algorithms

Lists of medical codes (Read codes) specific and non-specific for COPD were created prior to the study initiation. Read codes are a hierarchical clinical coding system of over 80,000

terms that are used in general practice in the UK and are entered by the GP into Vision Software. [12] These data are then uploaded by CPRD after they have been processed and quality checked added to the CPRD database for research use. Specific COPD codes consisted of codes listing either COPD or COPD specific entities of emphysema (see supplementary material for specific codes included). Non-specific codes consisted of a variety of lung diseases that could potentially be COPD e.g. chronic bronchitis. Combinations of codelists and additional material in the form of the presence of spirometry or COPD medications (see supplementary material) were used to make up the eight algorithms. The first four definitions required a specific COPD diagnosis code, with the first three requiring additional documentation (e.g., medication and/or spirometry). The other four definitions required non-specific bronchitis or respiratory symptom codes, with the least specific definition requiring only respiratory symptom codes. Details regarding each algorithm can be found in the supplementary material. Briefly, the eight algorithms were defined as follows, from the expected most specific to most sensitive construct:

1. Specific COPD code and more than one prescription of a COPD medication and presence of spirometry (**COPD Code + spirometry + COPD medication)**
2. Specific COPD code and presence of spirometry (**COPD Code + spirometry)**
3. Specific COPD code and more than one prescription of a COPD medication (**COPD Code + COPD medication)**
4. Specific COPD code only (**COPD Code only)**
5. Non-specific bronchitis code and more than one prescription of a COPD medication (**Bronchitis + COPD medication)**
6. Non-specific bronchitis code only (**Bronchitis only)**
7. Respiratory symptoms and presence of spirometry. Respiratory symptoms consisted of persistent cough, sputum production, or dyspnoea. (**Symptoms + spirometry)**
8. Respiratory symptom definition only (**Symptoms only)**

For peer review only

The presence of spirometry for inclusion in the algorithm was based on an existence of a record of a specific value or a Read code for spirometry rather than examining the actual value. We were able to assess interpretation of spirometry in the subset of patients who had flow volume loops or clinic letters attached and where actual FEV_1 and FVC values were available in CPRD.

Study Population

The study population consisted of a random sample of individuals selected from all subjects registered in CPRD with cohort entry being on or after the 1st January 2004. At cohort entry, people included had to be: over 35 years old, with at least 1 year historical data, and a possible diagnosis of COPD defined as evidence of having ever smoked and a record of a least one specific or possible COPD code or respiratory symptoms suggestive of COPD. The presence of the algorithms was ascertained during a period between patient cohort entry and the 31st December 2012. Patients had to be alive within 4 months of the last collection date of CPRD data for the January 2013 data build for inclusion in the analysis so that CPRD could access their medical records and additional information. For the main analysis, a patient could contribute to one algorithm only. It was possible for an individual to be eligible for more than one algorithm depending upon the codes used in their medical record over the study period. Individuals were randomly selected from the algorithm with the fewest number of participants first and then removed from the cohort so they could not be selected for another algorithm.

CPRD mailed a short, structured questionnaire to GPs in charge of randomly selected patients requesting confirmation of COPD status as well as any available specific information from the individual's medical record including spirometry print outs and hospital respiratory outpatient letters (see supplementary material). Data were "twice encrypted" within CPRD to ensure anonymity; firstly between practices and CPRD and secondly from CPRD to researchers. In the questionnaire the GP was asked whether or not the patient had a

For peer review only

diagnosis of COPD, what that diagnosis was based on, whether or not the patient had seen a respiratory physician and if they had any other respiratory diagnoses. A pilot set of 20 questionnaires were sent to GPs to assess the quality of the questionnaire. In total, 951 questionnaires were sent out assuming an 80% response rate.

Primary outcome

The primary outcome was identification of a diagnosis of COPD according to the pre-defined eight algorithms. The gold standard for the diagnosis of COPD was the decision made after respiratory physicians independently reviewed the evidence from the GP (questionnaire response with or without- additional evidence). Where they did not agree, a 3rd independent physician decided. Additionally, GP diagnosis of COPD was validated in a subset of patients where the GP provided supportive information including spirometry print-outs and hospital letters. This also allowed review of spirometry interpretation in some cases. Although we used two respiratory physicians independently as the gold standard for diagnosing COPD, this was done by reviewing the questionnaire sent to the GP (see appendix) as well as any additional information the GP sent which supported that diagnosis. This supporting information ranged from free text in the GP database to spirometry printouts done in the GP surgery to letters from secondary care. Therefore we were not solely relying on information from secondary care to make the diagnosis unless the GP decided to share that information. In this way we were able to include and validate the diagnosis of COPD in people who were not seen in secondary care.

Analysis

The primary analysis focused on the accuracy of identification of a COPD diagnosis in each of the pre-defined algorithms as defined by positive predicted value (PPV); i.e. the proportion of “true positives” (individuals with COPD) in each algorithm as determined by the gold standard. In addition, within each algorithm, where additional information was provided (lung function, hospital clinic letters), we calculated the accuracy of the GP diagnosis of COPD relative to the gold standard. This allowed review of spirometry interpretation in some cases.

For peer review only

We assessed the impact of commonly occurring co-morbidities on the accuracy of the pre-specified algorithms stratifying for cardiovascular co-morbidity, previous asthma diagnosis, smoking status and, where possible, Global Initiative for Chronic Obstructive Lung Disease (GOLD) staging of airflow limitation severity.[13] Cardiovascular co-morbidity included angina, history of myocardial infarction, previous coronary artery bypass graft / percutaneous coronary intervention or heart failure, but not hypertension due to its lack of overlap of symptoms that could mimic COPD. All covariates for stratification analysis were derived from information available up to cohort entry.

As a post hoc analysis individuals were eligible to be placed into multiple algorithms where possible, and the PPV was calculated for all individuals who had a specific COPD code compared to those with a specific COPD code and additional information (either spirometry or a COPD medication).

Assessment of possible trends in COPD diagnosis recording were also evaluated, including temporal trends in codes used and diagnostic specificity from 2004 to 2011. In addition, we compared our specific COPD codes to those recommended for use by QOF (see supplementary material); [14,15] H31% (excluding H3101 (smokers cough), H31y0 (chronic tracheitis) and H3122 (acute exacerbation of COPD)) H32% H36-H3z (excluding H3y0 and H3y1).

Sample size calculation

Our sample size for each algorithm was chosen to achieve accuracy of the true positives or PPV ±0.08 based on the reviewing physician judgment as the gold standard. Assuming an estimated PPV of 0.85 for any one algorithm, we required a sample of at least 77 individuals in each algorithm to achieve the desired accuracy (95% CI ±0.08). All analyses were performed using STATA 13.

For peer review only - http://bmjopen.bmj.com/site/about/guidelines.xhtml

Ethics approval was obtained from ISAC (the Independent Scientific Advisory Committee overseeing CPRD); protocol 12_065 and the LSHTM ethics committee.

RESULTS

Nine hundred and fifty one questionnaires were sent to GPs (see **Figure 1** for patient selection). Of those, 738 (77.6%) were returned, 704 (74.0%) met quality control standards and were not duplicates, and 696 (73.2%) could be included in the final analysis (8 had "uncertain" COPD diagnosis and no supporting documentation and were therefore excluded).

Among those included in the final analysis, additional evidence for the diagnosis of COPD was available for 272 patients. This represented 39.1% of the total study population, or 67.7% of the 402 patients with a confirmed COPD diagnosis in the study.

Overall, irrespective of the qualifying algorithm, 402 patients (57.8%) were considered to have a diagnosis of COPD based on reviewing physician judgment. **Table 1** shows the characteristics of the 696 patients included in the final analysis who were considered to possibly have COPD based on the inclusion criteria. On average, patients were in their mid-60s to early 70s across all algorithms. Approximately two-thirds of them were current smokers and one-quarter had a history of asthma. Generally, there were fewer patients with supporting information and cardiovascular co-morbidity in the less specific algorithms (4-8).

For peer review only

Table 1: Characteristics of the 696 patients included in the final study analysis

Algorithm		COPD Code + spirometry + COPD medication	COPD Code + spirometry	COPD Code + COPD medication	COPD Code only	Bronchitis + COPD medication	Bronchitis only	Symptoms + spirometry	Symptoms only
**Number of individuals (%)**		85 (100)	79 (100)	88 (100)	89 (100)	98 (100)	84 (100)	83 (100)	90 (100)
**Number (%) with supporting info**		46 (54.1)	44 (55.7)	48 (54.5)	40 (44.9)	32 (32.7)	18 (21.4)	30 (36.1)	14 (15.6)
**Mean age (sd)**		68.7 (11.3)	68.3 (11.7)	71.8 (10.5)	71.1 (10.4)	68.5 (13.1)	67.8 (13.4)	65.9 (11.9)	63.4 (14.1)
**Male (%)**		45 (52.9)	41 (51.9)	40 (45.5)	44 (49.4)	31 (31.6)	29 (34.5)	43 (51.8)	47 (52.2)
**Current smoker (%)**		49 (57.7)	50 (63.3)	55 (62.5)	47 (52.8)	66 (67.4)	61 (72.6)	48 (57.8)	58 (64.4)
**GOLD stage* (n=465)**	**1**	13 (16.3)	14 (18.0)	13 (15.9)	16 (20.5)	17 (28.8)	13 (35.1)	8 (20.0)	4 (36.4)
	**2**	43 (53.8)	48 (61.5)	41 (50.0)	46 (59.0)	31(52.5)	13 (35.1)	22 (55.0)	5 (45.5)
	**3**	18 (22.5)	13 (16.7)	22 (26.8)	12 (15.4)	9 (15.3)	8 (21.6)	8 (20.0)	2 (18.2)
	**4**	6 (7.5)	3 (3.9)	6 (7.3)	4 (5.1)	2 (3.4)	3 (8.1)	2 (5.0)	0 (0.0)
**History of cardiovascular disease**		20 (23.5)	22 (27.8)	17 (19.3)	18 (20.2)	20 (20.4)	17 (20.2)	14 (16.9)	9 (10.0)
**History of asthma**		18 (21.2)	20 (25.3)	16 (18.2)	15 (16.9)	27 (27.6)	23 (27.4)	19 (22.9)	23 (25.6)
**Mean BMI (sd) (n=575)**		N=83<br>27.5 (5.4)	N=78<br>26.7 (5.8)	N=86<br>26.4 (4.7)	N=87<br>27.8 (5.4)	N=98<br>27.4 (5.1)	N=85<br>28.1 (5.0)	N=41<br>27.1 (4.5)	N=17<br>27.6 (4.7)

*GOLD staging was ascertained from CPRD records or from supplementary information provided by GPs.

For peer review only

The number of patients diagnosed with COPD confirmed by the gold standard and the PPV for each algorithm is given in **Table 2**. The PPV was greatest for algorithms 1-4. Further data is available in the supplementary material regarding the effect of co-morbidities (**Table S1**), smoking status (**Table S2**) and GOLD staging (**Table S3**) on the performance of each algorithm.

Table 2: The PPV and proportion of patients diagnosed with COPD within each algorithm

Algorithm	Number of questionnaires sent out (n=951)	Number evaluable returned (n=696) (%)	Number with confirmed COPD	PPV and 95% CI
**COPD Code + spirometry + COPD medication**	119	85 (71.4)	76	89.4, 80.7-94.5
**COPD Code + spirometry**	119	79 (66.4)	67	83.8, 73.7-90.4
**COPD Code + COPD medication**	119	88 (73.9)	77	87.5, 78.6-93.0
**COPD Code only**	119	89 (74.8)	77	86.5, 77.5-92.3
**Bronchitis + COPD medication**	119	98 (82.4)	44	44.4, 34.8-54.5
**Bronchitis only**	119	84 (70.6)	26	29.5, 20.8-40.1
**Symptoms + spirometry**	119	83 (69.7)	37	43.5, 33.2-54.4
**Symptoms only**	118	90 (75.6)	11	12.2, 6.8-20.9

In a subset of 272 patients where additional evidence was available (in the form of spirometry print outs or hospital outpatient letters), we assessed accuracy of GP diagnosis of COPD. Overall, the PPV in this group was 95.0% (91.1-97.2), This is broken down by algorithm in **Table 3**. While the presence of supporting evidence improved the PPV in each group, algorithms 1-4 were still most accurate.

For peer review only - http://bmjopen.bmj.com/site/about/guidelines.xhtml

Table 3: PPV by algorithm where evidence was available to assess GP compared to physician diagnosis of COPD

Algorithm	Total number with evidence (N=272)	Number of patients with COPD confirmed by GOLD standard (N=220)	Number with COPD according to GP	PPV and 95% CI
**COPD Code + spirometry + COPD medication**	46	46	46	100
**COPD Code + spirometry**	44	35	33	94.3, 82.4-98.9
**COPD Code + COPD medication**	48	43	41	95.3, 82.4-98.9
**COPD Code only**	40	34	33	97.1, 80.2-99.6
**Bronchitis + COPD medication**	32	21	19	90.5, 66.0-97.9
**Bronchitis only**	18	12	11	91.7, 49.9-99.2
**Symptoms + spirometry**	30	21	20	95.2, 69.1-99.4
**Symptoms only**	14	8	6	75.0, 27.6-95.9

Post-hoc analysis

We repeated the analysis allowing individuals to be put into more than one algorithm and tested the PPV of COPD by algorithm relative to the gold standard where individuals were identified using only the presence of a specific COPD code (n=457) the PPV was identical to if they had a COPD code and evidence either in the form of spirometry or COPD medication prescription (n=454); PPV 83.0% (79.0-86.0) for both. In the majority of cases, where a specific COPD code had been assigned, there was additional evidence. Only 3 individuals had a specific COPD code with no additional evidence.

DISCUSSION

We tested the accuracy of eight different algorithms for identifying COPD within the CPRD among patients with suspected COPD (e.g., >35 years of age, smoking history, and recording of respiratory symptoms or COPD codes). The physician reviewer consensus was the GOLD standard. The best performing algorithm allowed an accurate ascertainment of 90% of patients as diagnosed with COPD. This consisted of a combination of a specific COPD code, more than one prescription of a COPD medication and spirometry (PPV 89.4, 95% CI: 80.7-94.5). The worst performing algorithm was represented by presence of

For peer review only - http://bmjopen.bmj.com/site/about/guidelines.xhtml

respiratory symptoms only (PPV12.2, 95%CI: 6.8-20.9). We found that any algorithm containing a specific COPD code performed better than those without (Algorithms 1-4). In a post hoc analysis, where we allowed individuals to populate more than one algorithm, we established that the use of additional information such as spirometry or medications in an algorithm to a specific COPD Read code alone did not increase the PPV. This suggests that the presence of a specific COPD Read code alone is sufficient to accurately identify patients with COPD from the database. Some study protocols require the presence of obstructive spirometry for identification of COPD patients; however, this study demonstrates that it may be unnecessary. This is particularly important as certain groups of individuals are less likely to have spirometry, namely women and patients <50 and >80 years of age. [8] This is also important as the minimal precision lost by not including spirometry and medications in the algorithm, allows an increase in the number of individuals who can potentially be included in a study. Using the whole of CPRD, we identified individuals with COPD using a specific COPD code only compared to a specific code plus medication and spirometry and found an increase in potential sample size for study of 10% using a specific COPD code only.

One of the advantages of this study was our ability to investigate both the accuracy of algorithms when identifying COPD patients within the CPRD, and accuracy of the actual GP diagnosis of COPD. When validating the GP diagnosis of COPD with a respiratory physician diagnosis as gold standard based on extra evidence provided by the GP, there was improved accuracy (PPV) across all algorithms, with algorithms 1-4 again performing best. This suggests that additional evidence is collected when the GP is reasonably certain that the patient has COPD. There was good concordance between GP and respiratory physician diagnosis suggesting respiratory consultant validation is not always needed. Where there was disagreement, this was usually because lung function did not meet criteria for COPD.

For peer review only - http://bmjopen.bmj.com/site/about/guidelines.xhtml

We found that the diagnostic accuracy of COPD decreased across all algorithms when patients also had a diagnosis of cardiovascular disease or asthma (Table S1 online supplement). When patients had a concomitant diagnosis of asthma, the presence of spirometry was particularly important to improve accuracy of COPD diagnosis. This was predominantly due to the fact spirometry had been misinterpreted. However, stratification led to smaller sample sizes, which could have impacted the precision of estimates. Unsurprisingly, the addition of the use of any inhaled COPD medication to the algorithm did not improve precision, most likely due to the overlap in medications used to treat both asthma and COPD (see online supplement). The algorithm accuracy was not affected by smoking status (current vs. ex-smoker) (Table S2 online supplement). We only included current or former smokers in our analysis, and cannot be sure of the validity of the results in a patient who has never smoked. Certainly in the UK, the majority of COPD is related to tobacco smoking and we hypothesized that fixed airflow obstruction in a non-smoker would most likely be due to chronic asthma.

When considering the severity of COPD by GOLD classification, algorithms 2 and 3, i.e. a specific COPD code and spirometry or COPD medications had the greatest accuracy for patients with mild disease (GOLD stage 1) (Table S3 online supplement). PPV increased with increasing disease severity. PPV increases with disease prevalence, and the prevalence of COPD increased moving from algorithm 8 to algorithm 1.

We considered the timing of diagnosis in view of an increased uptake of spirometry in primary care in more recent years and changes in QOF requirements over time during our study period (Table S4 online supplement). We found that algorithms 1-4 still had the greatest accuracy, but the PPV estimates were better for the post 2008 period than pre 2008 period (see supplementary material). Non-specific bronchitis codes and symptom codes were more likely to be used before 2008 rather than post. This may require consideration

For peer review only - http://bmjopen.bmj.com/site/about/guidelines.xhtml

when developing retrospective cohorts for analysis and otherwise suggests QOF has had a positive effect on the consistency of codes used for COPD diagnoses. However, our codes were more specific than QOF codes and some of the codes included in QOF were not included in our specific COPD codelist, but were included in the non-specific bronchitis codes. While we cannot comment on the accuracy of QOF codes, it is important to highlight that that some QOF codes are not disease specific and may not be a good way of identifying COPD patients from electronic health records as inevitably people without COPD will be included. It is also important to highlight that Read codes change over time with new codes added and some removed, and this needs consideration when identifying people with COPD.

There are limited COPD validation studies in electronic health records published in the literature to which we can compare our study. Soriano validated COPD in the GPRD in 2001, when OXMIS codes were still in use. [4] A Swedish study using a Swedish inpatient registry used ICD-9 and ICD-10 codes and identified COPD patients with similar accuracy.[16] A Canadian study in the Canadian primary care sentinel surveillance network used algorithms to identify several long term conditions and also had a PPV similar to ours for COPD [17]. However, all of these studies used different codes, algorithms and databases.

Our analysis has several limitations. We appreciate that using the gold standard of GP questionnaire and respiratory physician review is not perfect as when asked about whether or not a specific patient has a certain diagnosis, the GP is most likely to simply look in the electronic health record and see if that diagnosis has been record. However, there is no alternative. The overall response rate for this study was acceptable (77.6%), the proportion of questionnaires accompanied with additional evidence allowing for adjudication was rather low. We chose PPV as the measure of accuracy in this study to allow us to determine the probability that a patient had COPD from their electronic health record. PPV is correlated

For peer review only - http://bmjopen.bmj.com/site/about/guidelines.xhtml

with disease prevalence, and although it is strongly related to specificity, the actual estimates of specificity, sensitivity and negative predictive value cannot be determined from our data. Further, GP practices are self selecting with respect to their contribution to CPRD, however those practices appear to be representative of the UK population. Very few patients within contributing practices refuse to participate at an individual level and this is not thought to bias the results. While CPRD is representative of the general population, as with all validation studies that require a response, we cannot be sure that our sample is representative of GPs who have not responded, although there is unlikely to be any difference. The amount of missing data among the responding questionnaires was low, suggesting reasonable data quality. One of the other limitations of this study is that patients had to be alive to be included; however it is unlikely that coding would be different for individuals who are no longer alive.

The algorithm that consisted of a specific COPD code, COPD medication and spirometry had the highest PPV; however the PPV was almost as high when a specific COPD code alone was used. The poorest performing algorithms were those that involved bronchitis codes or respiratory symptoms; we would not recommend using these algorithms to identify COPD patients. In conclusion, we have shown that the presence of a specific COPD Read code alone is sufficient to identify patients with COPD from electronic health records such as CPRD. Minimal precision lost by not including spirometry and medications in the algorithm, allows an increase in the number of individuals who can potentially be included in a study by up to 10%. However by not including spirometry in the definition, the ability to stage COPD according to GOLD stages may not be possible for all COPD patients included in a study.

For peer review only - http://bmjopen.bmj.com/site/about/guidelines.xhtml

Acknowledgments: none

Contributorship statement: JKQ, HM, RLD, SE, KD and LS contributed to the conception and design, JKQ, HM, JH, to acquisition and analysis, JKQ, HM, RLD, HF, SE, JH, KD and LS to interpretation of data, and JKQ, HM, RLD, HF, SE, JH, KD and LS to the drafting of the manuscript. JKQ is responsible for the overall content as guarantor.

Competing interests: none

Funding: This project was funded jointly by GSK and the MRC. JKQ is funded on a MRC Population Health Scientist Fellowship. This work was part funded by a MRC Industry Partnership award (grant number G0902135).

Data Sharing Statement: No additional data are available.

For peer review only

For peer review only - http://bmjopen.bmj.com/site/about/guidelines.xhtml

REFERENCES

1. National Statistics. www.statistics.gov.uk date accessed June 2013.

2. Shahab LM, Jarvis MJ, Britton J, et al. Prevalence, diagnosis and relation to tobacco dependence of chronic obstructive pulmonary disease in a nationally representative population sample *Thorax* 2006;61(12):1043

3. file:///C:/Users/ENCDJQUI/Downloads/Invisible-Lives-report.pdf Invisible Lives. Chronic Obstructive Pulmonary Disease (COPD) – finding the missing millions. British Lung Foundation. November 2007. Accessed February 2014.

4. Soriano JB, Maier WC, Visick G, et al. Validation of general practitioner-diagnosed COPD in the UK General Practice. *European Journal of Epidemiology* 2001;17:1075–1080

5. NICE guideline COPD http://guidance.nice.org.uk/CG101/QuickRefGuide/pdf/English date accessed January 2014.

6. Cooke CR, Joo MJ, Anderson SM et al. The validity of using ICD-9 codes and pharmacy records to identify patients with chronic obstructive pulmonary disease. *BMC Health Services Research* 2011;11:37

7. Smith CJP, Gribbin J, Challen KB, et al. The impact of the 2004 NICE guideline and 2003General Medical Services contract on COPD in primary care in the UK. *Q J Med* 2008;101:145–153.

8. http://www.nice.org.uk/aboutnice/qof/qof.jsp. Accessed January 2014.

For peer review only - http://bmjopen.bmj.com/site/about/guidelines.xhtml

9. CPRD http://www.cprd.com (accessed February 28,2013)

10. Williams T, VanStaa T, Padmanabhan S et al. Recent advances in utility and use of the General Practice Research Database as an example of a UK Primary Care Data resource. *Ther Adv Drug Saf* 2012;3:88–99.

11. Tate AR, Beloff N, Al-Radwan B et al. Exploiting the potential of large databases of electronic health records for research using rapid search algorithms and an intuitive query interface. *J Am Med Inform Assoc*. 2014;21(2):292-8.

12. Chisholm J. The Read clinical classification. BMJ, 1990. 300(6732):1092.

13. GOLD 2011 http://www.goldcopd.org/uploads/users/files/GOLD2011_Summary.pdf accessed Nov 2013.

14. https://mqi.ic.nhs.uk/IndicatorDefaultView.aspx?ref=1.09.03.07 (QOF COPD). Accessed Feb 2014.

15. http://www.nbmedical.com/pdf/keep_simple_qof_2014-2014.pdf accessed Feb 2014

16. Inghammar M, Engström G, Löfdahl C-G et al. Validation of a COPD diagnosis from the Swedish Inpatient Registry *Scandinavian Journal of Public Health*, 2012;40: 773–776

17. Kadhim-Saleh A, Green M, WilliamsonT et al. Validation of the Diagnostic Algorithms for 5 Chronic Conditions in the Canadian Primary Care Sentinel

For peer review only

For peer review only - http://bmjopen.bmj.com/site/about/guidelines.xhtml

Surveillance Network (CPCSSN): A Kingston Practice-based Research Network (PBRN) Report. *J Am Board Fam Med* 2013;26: 159–167

Figure 1: Study population

For peer review only

For peer review only - http://bmjopen.bmj.com/site/about/guidelines.xhtml

Validation of Chronic Obstructive Pulmonary Disease (COPD) recording in the Clinical Practice Research Datalink (CPRD-GOLD)

Jennifer K Quint[1], Hana Müllerova[2], Rachael L DiSantostefano[3], Harriet Forbes[1], Susan Eaton[4], John R Hurst[5], Kourtney Davis[2], Liam Smeeth[1].

[1]Department of Non-communicable disease Epidemiology, London School of Hygiene and Tropical Medicine, London, UK
[2] Respiratory Epidemiology, GlaxoSmithKline R&D, Uxbridge, UK
[3] Respiratory Epidemiology, GlaxoSmithKline R&D, RTP, NC USA
[4] Clinical Practice Research Datalink Group, Medicines and Healthcare products Regulatory Agency, London, UK
[5]UCL Respiratory Medicine, Royal Free Campus, University College London Medical School, London, UK

Corresponding author:
Dr. Jennifer K Quint
Department of Non-communicable disease Epidemiology
London School of Hygiene and Tropical Medicine
Keppel Street, London WC1E 7HT
Tel: 0207 927 2622
Email: **Jennifer.quint@lshtm.ac.uk**

Keywords: COPD, diagnosis of COPD, validation, spirometry, electronic health records

Word count: 3448

Contributorship statement: JKQ, HM, RLD, SE, KD and LS contributed to the conception and design, JKQ, HM, JH, to acquisition and analysis, JKQ, HM, RLD, HF, SE, JH, KD and LS to interpretation of data, and JKQ, HM, RLD, HF, SE, JH, KD and LS to the drafting of the manuscript. JKQ is responsible for the overall content as guarantor.

For peer review only

ABSTRACT

Objectives: The optimal method of identifying people with COPD from electronic primary care records is not known. We assessed the accuracy of different approaches using the Clinical Practice Research Datalink (CPRD), a UK electronic health record database.

Setting: Nine hundred and fifty one participants registered with a CPRD practice in the UK between 1st January 2004 and 31st December 2012. Individuals were selected for ≥1 of eight algorithms to identify people with COPD. General practitioners were sent a brief questionnaire and additional evidence to support a COPD diagnosis was requested. All information received was reviewed independently by two respiratory physicians whose opinion was taken as gold standard.

Primary outcome measure: The primary measure of accuracy was the positive predictive value (PPV); the proportion of people identified by each algorithm for whom COPD was confirmed.

Results: Nine hundred and fifty one questionnaires were sent and 738 (78%) returned. After quality control, 696 (73.2%) patients were included in the final analysis. All four algorithms including a specific COPD diagnostic code performed well. Using a diagnostic code alone the PPV was 86.5% (77.5% to 92.3%) while requiring a diagnosis plus spirometry plus specific medication the PPV was slightly higher at 89.4% (80.7% to 94.5%) but reduced case numbers by 10%. Algorithms without specific diagnostic codes had low PPVs (range 12.2% to 44.4%).

Conclusion: Patients with COPD can be accurately identified from UK primary care records using specific diagnostic codes. Requiring spirometry or COPD medications only marginally improved accuracy. The high accuracy applies since the introduction of an incentivised disease register for COPD as part of QOF (Quality and Outcomes Framework) in 2004.

For peer review only

Article Summary: This paper investigates how individuals with COPD can be accurately identified in electronic health records using specific COPD codes alone without additional information (i.e. presence of spirometry or respiratory medications). This information is essential for appropriate disease management, epidemiological research of COPD and other conditions, where COPD is an important co-morbidity.

Strengths and limitations of this study:

- We have shown that the presence of a specific COPD Read code alone is sufficient to identify patients with COPD from electronic health records. Minimal precision lost by not including spirometry and medications in the algorithm, allows an increase in the number of individuals who can potentially be included in a study by up to 10%.
- We were able to investigate both the accuracy of algorithms when identifying COPD patients within the CPRD, and accuracy of the actual GP diagnosis of COPD.
- The amount of missing data among the responding questionnaires was low, suggesting reasonable data quality.
- Although the overall response rate for this study was acceptable (77.6%), the proportion of questionnaires accompanied with additional evidence allowing for adjudication was lower.

No additional data are available. We would be happy to provide the ISAC protocol to reviewers if required.

INTRODUCTION

For peer review only

Chronic obstructive pulmonary disease (COPD) represents an enormous health burden worldwide. Currently COPD is the 4th leading cause of death and is predicted to become the 3rd by 2020.[1] There are approximately 835,000 people diagnosed with COPD in the UK and an estimated 2,200,000 people remain undiagnosed.[2,3]

Electronic health records in the UK provide an excellent resource in which to study COPD as they offer a large cohort size, the presence of disease severity indicators and long-term follow-up information on a patient's integrated medical history. Although multiple studies have been undertaken to investigate various aspects of COPD over the last 10 years in several electronic health record databases, there is no standard definition used to identify COPD in large databases and codelists used to identify patients with COPD vary by author. Over ten years ago, the diagnosis of COPD was validated in the Clinical Practice Research Datalink (CPRD-GOLD, formerly GPRD) using OXMIS codes.[4] This coding system is now obsolete in CPRD and Read codes are used as standard.

There is no single diagnostic test for COPD. The diagnosis of COPD relies on clinical judgement based on a combination of history, physical examination and confirmation of the presence of airflow obstruction using spirometry.[5] When retrospectively evaluating the accuracy of a COPD diagnosis, commonly used COPD definitions may misclassify patients as having COPD. Using multiple diagnostic codes in combination with pharmacy may improve the accuracy of identification of COPD patients.[6] Further, over the past 10 years the definition of COPD in clinical practice has evolved leading to changes in how COPD is diagnosed and the diagnosis recorded. With the introduction of the Quality and Outcomes Framework (QOF) codes for COPD in 2004 in England and Wales there are more codes available to identify COPD then there were previously. QOF is a voluntary incentive scheme for primary care physicians (GPs) in the UK which contains indicators against which GP practices can score points and hence be rewarded for how well they care for patients. [7] Evaluation of airflow limitation using spirometry is used as standard to confirm COPD

For peer review only

diagnosis and severity of COPD is part of the annual COPD patient review.[3,8] However, even spirometry if incorrectly performed or interpreted can lead to misdiagnosis (both over and under diagnosis of COPD) in approximately 20% of cases. It is also recognised that some subgroups of individuals (e.g. women and individuals under 50 and over 80) are less likely to have spirometry measured.[8]

This study aimed to improve our ability to identify patients with COPD within electronic health records. We used the CPRD, a widely used collection of computerised medical records which is commonly used for clinical and research purposes. CPRD is comparable to other electronic health record databases used in the UK. Our objective was to test the accuracy of different definitions of COPD in the CPRD using Positive Predictive Value (PPV), by comparing the database records with additional information provided by General Practitioners (GPs). This work is important for epidemiological research in COPD and diseases where COPD is an important co-morbidity as well as for clinical practice.

METHODS

Dataset

CPRD is the world's largest validated computerized database of anonymized longitudinal medical records for primary care. [9,10] Data comprise approximately 14 million patients with around 5.4 million of these being currently alive and registered from 660 primary care practices spread throughout the UK. Records are derived from a widely used GP software system and contain complete prescribing and coded diagnostic and clinical information as well as information on tests requested, laboratory results and referrals made at or following on from each consultation. [11]

Codelists and algorithms

Lists of medical codes (Read codes) specific and non-specific for COPD were created prior to the study initiation. Read codes are a hierarchical clinical coding system of over 80,000 terms that are used in general practice in the UK and are entered by the GP into Vision

For peer review only

Software. [12] These data are then uploaded by CPRD after they have been processed and quality checked added to the CPRD database for research use. Specific COPD codes consisted of codes listing either COPD or COPD specific entities of emphysema (see supplementary material for specific codes included). Non-specific codes consisted of a variety of lung diseases that could potentially be COPD e.g. chronic bronchitis. Combinations of codelists and additional material in the form of the presence of spirometry or COPD medications (see supplementary material) were used to make up the eight algorithms. The first four definitions required a specific COPD diagnosis code, with the first three requiring additional documentation (e.g., medication and/or spirometry). The other four definitions required non-specific bronchitis or respiratory symptom codes, with the least specific definition requiring only respiratory symptom codes. Details regarding each algorithm can be found in the supplementary material. Briefly, the eight algorithms were defined as follows, from the expected most specific to most sensitive construct:

1. Specific COPD code and more than one prescription of a COPD medication and presence of spirometry (**COPD Code + spirometry + COPD medication)**
2. Specific COPD code and presence of spirometry (**COPD Code + spirometry)**
3. Specific COPD code and more than one prescription of a COPD medication (**COPD Code + COPD medication)**
4. Specific COPD code only (**COPD Code only)**
5. Non-specific bronchitis code and more than one prescription of a COPD medication (**Bronchitis + COPD medication)**
6. Non-specific bronchitis code only (**Bronchitis only)**
7. Respiratory symptoms and presence of spirometry. Respiratory symptoms consisted of persistent cough, sputum production, or dyspnoea. (**Symptoms + spirometry)**
8. Respiratory symptom definition only (**Symptoms only)**

For peer review only

The presence of spirometry for inclusion in the algorithm was based on an existence of a record of a specific value or a Read code for spirometry rather than examining the actual value. We were able to assess interpretation of spirometry in the subset of patients who had flow volume loops or clinic letters attached and where actual FEV_1 and FVC values were available in CPRD.

Study Population

The study population consisted of a random sample of individuals selected from all subjects registered in CPRD with cohort entry being on or after the 1st January 2004. At cohort entry, people included had to be: over 35 years old, with at least 1 year historical data, and a possible diagnosis of COPD defined as evidence of having ever smoked and a record of a least one specific or possible COPD code or respiratory symptoms suggestive of COPD. The presence of the algorithms was ascertained during a period between patient cohort entry and the 31st December 2012. Patients had to be alive within 4 months of the last collection date of CPRD data for the January 2013 data build for inclusion in the analysis so that CPRD could access their medical records and additional information. For the main analysis, a patient could contribute to one algorithm only. It was possible for an individual to be eligible for more than one algorithm depending upon the codes used in their medical record over the study period. Individuals were randomly selected from the algorithm with the fewest number of participants first and then removed from the cohort so they could not be selected for another algorithm.

CPRD mailed a short, structured questionnaire to GPs in charge of randomly selected patients requesting confirmation of COPD status as well as any available specific information from the individual's medical record including spirometry print outs and hospital respiratory outpatient letters (see supplementary material). Data were "twice encrypted" within CPRD to ensure anonymity; firstly between practices and CPRD and secondly from CPRD to researchers. In the questionnaire the GP was asked whether or not the patient had a

For peer review only

diagnosis of COPD, what that diagnosis was based on, whether or not the patient had seen a respiratory physician and if they had any other respiratory diagnoses. A pilot set of 20 questionnaires were sent to GPs to assess the quality of the questionnaire. In total, 951 questionnaires were sent out assuming an 80% response rate.

Primary outcome

The primary outcome was identification of a diagnosis of COPD according to the pre-defined eight algorithms. The gold standard for the diagnosis of COPD was the decision made after respiratory physicians independently reviewed the evidence from the GP (questionnaire response with or without- additional evidence). Where they did not agree, a 3rd independent physician decided. Additionally, GP diagnosis of COPD was validated in a subset of patients where the GP provided supportive information including spirometry print-outs and hospital letters. This also allowed review of spirometry interpretation in some cases. Although we used two respiratory physicians independently as the gold standard for diagnosing COPD, this was done by reviewing the questionnaire sent to the GP (see appendix) as well as any additional information the GP sent which supported that diagnosis. This supporting information ranged from free text in the GP database to spirometry printouts done in the GP surgery to letters from secondary care. Therefore we were not solely relying on information from secondary care to make the diagnosis unless the GP decided to share that information. In this way we were able to include and validate the diagnosis of COPD in people who were not seen in secondary care.

Analysis

The primary analysis focused on the accuracy of identification of a COPD diagnosis in each of the pre-defined algorithms as defined by positive predicted value (PPV); i.e. the proportion of “true positives” (individuals with COPD) in each algorithm as determined by the gold standard. In addition, within each algorithm, where additional information was provided (lung function, hospital clinic letters), we calculated the accuracy of the GP diagnosis of COPD relative to the gold standard. This allowed review of spirometry interpretation in some cases.

For peer review only

We assessed the impact of commonly occurring co-morbidities on the accuracy of the pre-specified algorithms stratifying for cardiovascular co-morbidity, previous asthma diagnosis, smoking status and, where possible, Global Initiative for Chronic Obstructive Lung Disease (GOLD) staging of airflow limitation severity.[13] Cardiovascular co-morbidity included angina, history of myocardial infarction, previous coronary artery bypass graft / percutaneous coronary intervention or heart failure, but not hypertension due to its lack of overlap of symptoms that could mimic COPD. All covariates for stratification analysis were derived from information available up to cohort entry.

As a post hoc analysis individuals were eligible to be placed into multiple algorithms where possible, and the PPV was calculated for all individuals who had a specific COPD code compared to those with a specific COPD code and additional information (either spirometry or a COPD medication).

Assessment of possible trends in COPD diagnosis recording were also evaluated, including temporal trends in codes used and diagnostic specificity from 2004 to 2011. In addition, we compared our specific COPD codes to those recommended for use by QOF (see supplementary material); [14,15] H31% (excluding H3101 (smokers cough), H31y0 (chronic tracheitis) and H3122 (acute exacerbation of COPD)) H32% H36-H3z (excluding H3y0 and H3y1).

Sample size calculation

Our sample size for each algorithm was chosen to achieve accuracy of the true positives or PPV ±0.08 based on the reviewing physician judgment as the gold standard. Assuming an estimated PPV of 0.85 for any one algorithm, we required a sample of at least 77 individuals in each algorithm to achieve the desired accuracy (95% CI ±0.08). All analyses were performed using STATA 13.

For peer review only

Ethics approval was obtained from ISAC (the Independent Scientific Advisory Committee overseeing CPRD); protocol 12_065 and the LSHTM ethics committee.

RESULTS

Nine hundred and fifty one questionnaires were sent to GPs (see **Figure 1** for patient selection). Of those, 738 (77.6%) were returned, 704 (74.0%) met quality control standards and were not duplicates, and 696 (73.2%) could be included in the final analysis (8 had "uncertain" COPD diagnosis and no supporting documentation and were therefore excluded).

Among those included in the final analysis, additional evidence for the diagnosis of COPD was available for 272 patients. This represented 39.1% of the total study population, or 67.7% of the 402 patients with a confirmed COPD diagnosis in the study.

Overall, irrespective of the qualifying algorithm, 402 patients (57.8%) were considered to have a diagnosis of COPD based on reviewing physician judgment. **Table 1** shows the characteristics of the 696 patients included in the final analysis who were considered to possibly have COPD based on the inclusion criteria. On average, patients were in their mid-60s to early 70s across all algorithms. Approximately two-thirds of them were current smokers and one-quarter had a history of asthma. Generally, there were fewer patients with supporting information and cardiovascular co-morbidity in the less specific algorithms (4-8).

For peer review only

Table 1: Characteristics of the 696 patients included in the final study analysis

Algorithm		COPD Code + spirometry + COPD medication	COPD Code + spirometry	COPD Code + COPD medication	COPD Code only	Bronchitis + COPD medication	Bronchitis only	Symptoms + spirometry	Symptoms only
Number of individuals (%)		85 (100)	79 (100)	88 (100)	89 (100)	98 (100)	84 (100)	83 (100)	90 (100)
Number (%) with supporting info		46 (54.1)	44 (55.7)	48 (54.5)	40 (44.9)	32 (32.7)	18 (21.4)	30 (36.1)	14 (15.6)
Mean age (sd)		68.7 (11.3)	68.3 (11.7)	71.8 (10.5)	71.1 (10.4)	68.5 (13.1)	67.8 (13.4)	65.9 (11.9)	63.4 (14.1)
Male (%)		45 (52.9)	41 (51.9)	40 (45.5)	44 (49.4)	31 (31.6)	29 (34.5)	43 (51.8)	47 (52.2)
Current smoker (%)		49 (57.7)	50 (63.3)	55 (62.5)	47 (52.8)	66 (67.4)	61 (72.6)	48 (57.8)	58 (64.4)
GOLD stage* (n=465)	1	13 (16.3)	14 (18.0)	13 (15.9)	16 (20.5)	17 (28.8)	13 (35.1)	8 (20.0)	4 (36.4)
	2	43 (53.8)	48 (61.5)	41 (50.0)	46 (59.0)	31(52.5)	13 (35.1)	22 (55.0)	5 (45.5)
	3	18 (22.5)	13 (16.7)	22 (26.8)	12 (15.4)	9 (15.3)	8 (21.6)	8 (20.0)	2 (18.2)
	4	6 (7.5)	3 (3.9)	6 (7.3)	4 (5.1)	2 (3.4)	3 (8.1)	2 (5.0)	0 (0.0)
History of cardiovascular disease		20 (23.5)	22 (27.8)	17 (19.3)	18 (20.2)	20 (20.4)	17 (20.2)	14 (16.9)	9 (10.0)
History of asthma		18 (21.2)	20 (25.3)	16 (18.2)	15 (16.9)	27 (27.6)	23 (27.4)	19 (22.9)	23 (25.6)
Mean BMI (sd) (n=575)		N=83<br>27.5 (5.4)	N=78<br>26.7 (5.8)	N=86<br>26.4 (4.7)	N=87<br>27.8 (5.4)	N=98<br>27.4 (5.1)	N=85<br>28.1 (5.0)	N=41<br>27.1 (4.5)	N=17<br>27.6 (4.7)

*GOLD staging was ascertained from CPRD records or from supplementary information provided by GPs.

For peer review only

The number of patients diagnosed with COPD confirmed by the gold standard and the PPV for each algorithm is given in **Table 2**. The PPV was greatest for algorithms 1-4. Further data is available in the supplementary material regarding the effect of co-morbidities (**Table S1**), smoking status (**Table S2**) and GOLD staging (**Table S3**) on the performance of each algorithm.

Table 2: The PPV and proportion of patients diagnosed with COPD within each algorithm

**Algorithm**	**Number of questionnaires sent out (n=951)**	**Number evaluable returned (n=696) (%)**	**Number with confirmed COPD**	**PPV and 95% CI**
**COPD Code + spirometry + COPD medication**	119	85 (71.4)	76	89.4, 80.7-94.5
**COPD Code + spirometry**	119	79 (66.4)	67	83.8, 73.7-90.4
**COPD Code + COPD medication**	119	88 (73.9)	77	87.5, 78.6-93.0
**COPD Code only**	119	89 (74.8)	77	86.5, 77.5-92.3
**Bronchitis + COPD medication**	119	98 (82.4)	44	44.4, 34.8-54.5
**Bronchitis only**	119	84 (70.6)	26	29.5, 20.8-40.1
**Symptoms + spirometry**	119	83 (69.7)	37	43.5, 33.2-54.4
**Symptoms only**	118	90 (75.6)	11	12.2, 6.8-20.9

In a subset of 272 patients where additional evidence was available (in the form of spirometry print outs or hospital outpatient letters), we assessed accuracy of GP diagnosis of COPD. Overall, the PPV in this group was 95.0% (91.1-97.2), This is broken down by algorithm in **Table 3**. While the presence of supporting evidence improved the PPV in each group, algorithms 1-4 were still most accurate.

For peer review only

Table 3: PPV by algorithm where evidence was available to assess GP compared to physician diagnosis of COPD

Algorithm	Total number with evidence (N=272)	Number of patients with COPD confirmed by GOLD standard (N=220)	Number with COPD according to GP	PPV and 95% CI
**COPD Code + spirometry + COPD medication**	46	46	46	100
**COPD Code + spirometry**	44	35	33	94.3, 82.4-98.9
**COPD Code + COPD medication**	48	43	41	95.3, 82.4-98.9
**COPD Code only**	40	34	33	97.1, 80.2-99.6
**Bronchitis + COPD medication**	32	21	19	90.5, 66.0-97.9
**Bronchitis only**	18	12	11	91.7, 49.9-99.2
**Symptoms + spirometry**	30	21	20	95.2, 69.1-99.4
**Symptoms only**	14	8	6	75.0, 27.6-95.9

Post-hoc analysis

We repeated the analysis allowing individuals to be put into more than one algorithm and tested the PPV of COPD by algorithm relative to the gold standard where individuals were identified using only the presence of a specific COPD code (n=457) the PPV was identical to if they had a COPD code and evidence either in the form of spirometry or COPD medication prescription (n=454); PPV 83.0% (79.0-86.0) for both. In the majority of cases, where a specific COPD code had been assigned, there was additional evidence. Only 3 individuals had a specific COPD code with no additional evidence.

DISCUSSION

We tested the accuracy of eight different algorithms for identifying COPD within the CPRD among patients with suspected COPD (e.g., >35 years of age, smoking history, and recording of respiratory symptoms or COPD codes). The physician reviewer consensus was the GOLD standard. The best performing algorithm allowed an accurate ascertainment of 90% of patients as diagnosed with COPD. This consisted of a combination of a specific COPD code, more than one prescription of a COPD medication and spirometry (PPV 89.4, 95% CI: 80.7-94.5). The worst performing algorithm was represented by presence of

For peer review only - http://bmjopen.bmj.com/site/about/guidelines.xhtml

respiratory symptoms only (PPV12.2, 95%CI: 6.8-20.9). We found that any algorithm containing a specific COPD code performed better than those without (Algorithms 1-4). In a post hoc analysis, where we allowed individuals to populate more than one algorithm, we established that the use of additional information such as spirometry or medications in an algorithm to a specific COPD Read code alone did not increase the PPV. This suggests that the presence of a specific COPD Read code alone is sufficient to accurately identify patients with COPD from the database. Some study protocols require the presence of obstructive spirometry for identification of COPD patients; however, this study demonstrates that it may be unnecessary. This is particularly important as certain groups of individuals are less likely to have spirometry, namely women and patients <50 and >80 years of age. [8] This is also important as the minimal precision lost by not including spirometry and medications in the algorithm, allows an increase in the number of individuals who can potentially be included in a study. Using the whole of CPRD, we identified individuals with COPD using a specific COPD code only compared to a specific code plus medication and spirometry and found an increase in potential sample size for study of 10% using a specific COPD code only.

One of the advantages of this study was our ability to investigate both the accuracy of algorithms when identifying COPD patients within the CPRD, and accuracy of the actual GP diagnosis of COPD. When validating the GP diagnosis of COPD with a respiratory physician diagnosis as gold standard based on extra evidence provided by the GP, there was improved accuracy (PPV) across all algorithms, with algorithms 1-4 again performing best. This suggests that additional evidence is collected when the GP is reasonably certain that the patient has COPD. There was good concordance between GP and respiratory physician diagnosis suggesting respiratory consultant validation is not always needed. Where there was disagreement, this was usually because lung function did not meet criteria for COPD.

For peer review only - http://bmjopen.bmj.com/site/about/guidelines.xhtml

We found that the diagnostic accuracy of COPD decreased across all algorithms when patients also had a diagnosis of cardiovascular disease or asthma (Table S1 online supplement). When patients had a concomitant diagnosis of asthma, the presence of spirometry was particularly important to improve accuracy of COPD diagnosis. This was predominantly due to the fact spirometry had been misinterpreted. However, stratification led to smaller sample sizes, which could have impacted the precision of estimates. Unsurprisingly, the addition of the use of any inhaled COPD medication to the algorithm did not improve precision, most likely due to the overlap in medications used to treat both asthma and COPD (see online supplement). The algorithm accuracy was not affected by smoking status (current vs. ex-smoker) (Table S2 online supplement). We only included current or former smokers in our analysis, and cannot be sure of the validity of the results in a patient who has never smoked. Certainly in the UK, the majority of COPD is related to tobacco smoking and we hypothesized that fixed airflow obstruction in a non-smoker would most likely be due to chronic asthma.

When considering the severity of COPD by GOLD classification, algorithms 2 and 3, i.e. a specific COPD code and spirometry or COPD medications had the greatest accuracy for patients with mild disease (GOLD stage 1) (Table S3 online supplement). PPV increased with increasing disease severity. PPV increases with disease prevalence, and the prevalence of COPD increased moving from algorithm 8 to algorithm 1.

We considered the timing of diagnosis in view of an increased uptake of spirometry in primary care in more recent years and changes in QOF requirements over time during our study period (Table S4 online supplement). We found that algorithms 1-4 still had the greatest accuracy, but the PPV estimates were better for the post 2008 period than pre 2008 period (see supplementary material). Non-specific bronchitis codes and symptom codes were more likely to be used before 2008 rather than post. This may require consideration

For peer review only

For peer review only - http://bmjopen.bmj.com/site/about/guidelines.xhtml

when developing retrospective cohorts for analysis and otherwise suggests QOF has had a positive effect on the consistency of codes used for COPD diagnoses. However, our codes were more specific than QOF codes and some of the codes included in QOF were not included in our specific COPD codelist, but were included in the non-specific bronchitis codes. While we cannot comment on the accuracy of QOF codes, it is important to highlight that that some QOF codes are not disease specific and may not be a good way of identifying COPD patients from electronic health records as inevitably people without COPD will be included. It is also important to highlight that Read codes change over time with new codes added and some removed, and this needs consideration when identifying people with COPD.

There are limited COPD validation studies in electronic health records published in the literature to which we can compare our study. Soriano validated COPD in the GPRD in 2001, when OXMIS codes were still in use. [4] A Swedish study using a Swedish inpatient registry used ICD-9 and ICD-10 codes and identified COPD patients with similar accuracy.[16] A Canadian study in the Canadian primary care sentinel surveillance network used algorithms to identify several long term conditions and also had a PPV similar to ours for COPD [17]. However, all of these studies used different codes, algorithms and databases.

Our analysis has several limitations. We appreciate that using the gold standard of GP questionnaire and respiratory physician review is not perfect as when asked about whether or not a specific patient has a certain diagnosis, the GP is most likely to simply look in the electronic health record and see if that diagnosis has been record. However, there is no alternative. The overall response rate for this study was acceptable (77.6%), the proportion of questionnaires accompanied with additional evidence allowing for adjudication was rather low. We chose PPV as the measure of accuracy in this study to allow us to determine the probability that a patient had COPD from their electronic health record. PPV is correlated

For peer review only - http://bmjopen.bmj.com/site/about/guidelines.xhtml

with disease prevalence, and although it is strongly related to specificity, the actual estimates of specificity, sensitivity and negative predictive value cannot be determined from our data. Further, GP practices are self selecting with respect to their contribution to CPRD, however those practices appear to be representative of the UK population. Very few patients within contributing practices refuse to participate at an individual level and this is not thought to bias the results. While CPRD is representative of the general population, as with all validation studies that require a response, we cannot be sure that our sample is representative of GPs who have not responded, although there is unlikely to be any difference. The amount of missing data among the responding questionnaires was low, suggesting reasonable data quality. One of the other limitations of this study is that patients had to be alive to be included; however it is unlikely that coding would be different for individuals who are no longer alive.

The algorithm that consisted of a specific COPD code, COPD medication and spirometry had the highest PPV; however the PPV was almost as high when a specific COPD code alone was used. The poorest performing algorithms were those that involved bronchitis codes or respiratory symptoms; we would not recommend using these algorithms to identify COPD patients. In conclusion, we have shown that the presence of a specific COPD Read code alone is sufficient to identify patients with COPD from electronic health records such as CPRD. Minimal precision lost by not including spirometry and medications in the algorithm, allows an increase in the number of individuals who can potentially be included in a study by up to 10%. However by not including spirometry in the definition, the ability to stage COPD according to GOLD stages may not be possible for all COPD patients included in a study.

Acknowledgments: none

Competing interests: none

For peer review only - http://bmjopen.bmj.com/site/about/guidelines.xhtml

Funding: This project was funded jointly by GSK and the MRC. JKQ is funded on a MRC Population Health Scientist Fellowship. This work was part funded by a MRC Industry Partnership award (grant number G0902135).

Figure 1: Study population

REFERENCES

1. National Statistics. www.statistics.gov.uk date accessed June 2013.

2. Shahab LM, Jarvis MJ, Britton J, et al. Prevalence, diagnosis and relation to tobacco dependence of chronic obstructive pulmonary disease in a nationally representative population sample *Thorax* 2006;61(12):1043

3. file:///C:/Users/ENCDJQUI/Downloads/Invisible-Lives-report.pdf Invisible Lives. Chronic Obstructive Pulmonary Disease (COPD) – finding the missing millions. British Lung Foundation. November 2007. Accessed February 2014.

4. Soriano JB, Maier WC, Visick G, et al. Validation of general practitioner-diagnosed COPD in the UK General Practice. *European Journal of Epidemiology* 2001;17:1075–1080

5. NICE guideline COPD http://guidance.nice.org.uk/CG101/QuickRefGuide/pdf/English date accessed January 2014.

For peer review only

For peer review only - http://bmjopen.bmj.com/site/about/guidelines.xhtml

6. Cooke CR, Joo MJ, Anderson SM et al. The validity of using ICD-9 codes and pharmacy records to identify patients with chronic obstructive pulmonary disease. *BMC Health Services Research* 2011;11:37

7. Smith CJP, Gribbin J, Challen KB, et al. The impact of the 2004 NICE guideline and 2003General Medical Services contract on COPD in primary care in the UK. *Q J Med* 2008;101:145–153.

8. http://www.nice.org.uk/aboutnice/qof/qof.jsp. Accessed January 2014.

9. CPRD http://www.cprd.com (accessed February 28,2013)

10. Williams T, VanStaa T, Padmanabhan S et al. Recent advances in utility and use of the General Practice Research Database as an example of a UK Primary Care Data resource. *Ther Adv Drug Saf* 2012;3:88–99.

11. Tate AR, Beloff N, Al-Radwan B et al. Exploiting the potential of large databases of electronic health records for research using rapid search algorithms and an intuitive query interface. *J Am Med Inform Assoc*. 2014;21(2):292-8.

12. Chisholm J. The Read clinical classification. BMJ, 1990. 300(6732):1092.

13. GOLD 2011 http://www.goldcopd.org/uploads/users/files/GOLD2011_Summary.pdf accessed Nov 2013.

14. https://mqi.ic.nhs.uk/IndicatorDefaultView.aspx?ref=1.09.03.07 (QOF COPD). Accessed Feb 2014.

For peer review only - http://bmjopen.bmj.com/site/about/guidelines.xhtml

15. http://www.nbmedical.com/pdf/keep_simple_qof_2014-2014.pdf accessed Feb 2014

16. Inghammar M, Engström G, Löfdahl C-G et al. Validation of a COPD diagnosis from the Swedish Inpatient Registry *Scandinavian Journal of Public Health*, 2012;40: 773–776

17. Kadhim-Saleh A, Green M, WilliamsonT et al. Validation of the Diagnostic Algorithms for 5 Chronic Conditions in the Canadian Primary Care Sentinel Surveillance Network (CPCSSN): A Kingston Practice-based Research Network (PBRN) Report. *J Am Board Fam Med* 2013;26: 159–167

For peer review only

For peer review only - http://bmjopen.bmj.com/site/about/guidelines.xhtml

Figure 1: Study sample

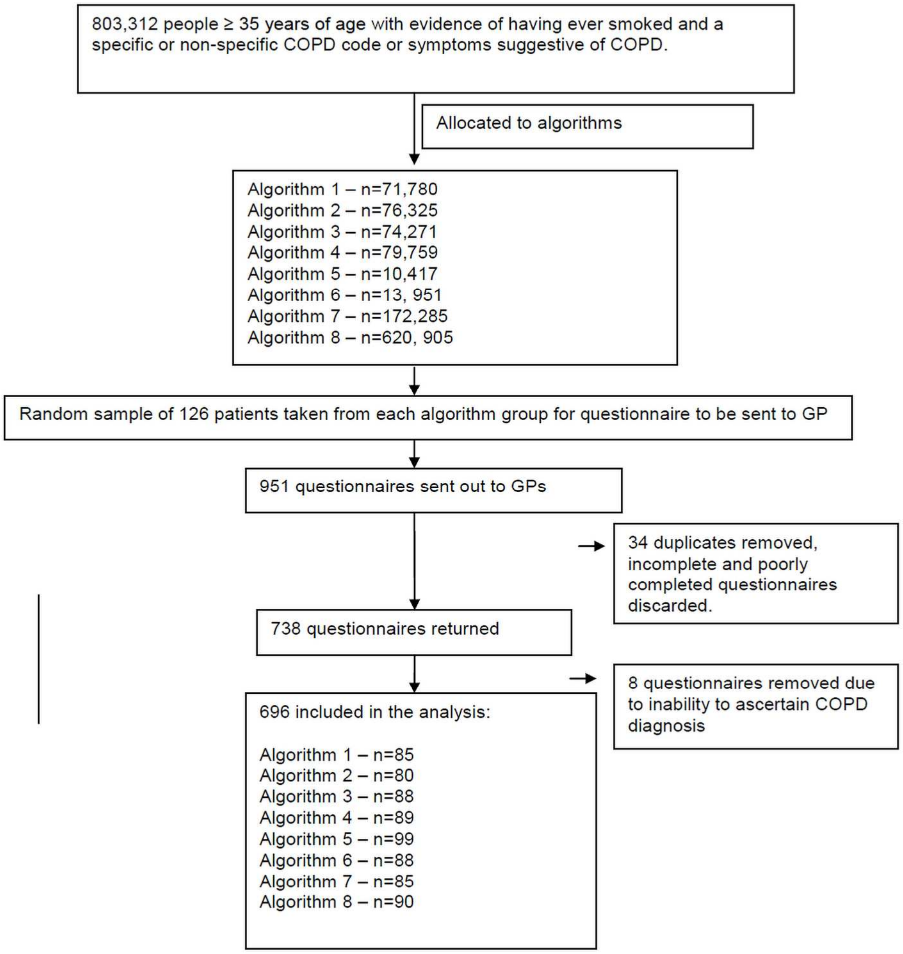

228x248mm (300 x 300 DPI)

Supplementary information

Algorithms

1. Specific COPD code and more than one prescription of a COPD medication and presence of spirometry. The first COPD medication had to be prescribed within 4 weeks of the COPD diagnostic code.
2. Specific COPD code and presence of spirometry
3. Specific COPD code and more than one prescription of a COPD medication. The first COPD medication had to be prescribed within 4 weeks of the COPD diagnostic code.
4. Specific COPD code only
5. Non-specific bronchitis codes and more than one prescription of a COPD medication. The first COPD medication had to be prescribed within 4 weeks of the COPD diagnostic code.
6. Non-specific bronchitis codes only
7. Respiratory symptoms and presence of spirometry. Respiratory symptoms consisted of persistent cough, sputum production, or dyspnoea
8. Respiratory symptom definition only

Specific COPD codes

medcode	term
18476	COPD follow-up
45771	Chronic obstructive pulmonary disease does not disturb sleep
4084	Airways obstructn irreversible
794	Emphysema
998	Chronic obstructive airways disease
1001	Chronic obstructive pulmonary disease
5710	Chronic obstructive airways disease NOS
9520	Chronic obstructive pulmonary disease monitoring
9876	Severe chronic obstructive pulmonary disease
10802	Moderate chronic obstructive pulmonary disease
10863	Mild chronic obstructive pulmonary disease
10980	Centrilobular emphysema
11287	Chronic obstructive pulmonary disease annual review
14798	Emphysematous bronchitis
18621	Chronic obstructive pulmonary disease follow-up
18792	Chronic obstructive pulmonary disease monitoring admin
23492	Chronic bullous emphysema NOS
26018	Chronic obstructive pulmonary disease monitoring by nurse
26306	Chronic bullous emphysema
28755	Chronic obstructive pulmonary disease monitoring 1st letter
33450	Emphysema NOS
34202	Chronic obstructive pulmonary disease monitoring 2nd letter
34215	Chronic obstructive pulmonary disease monitoring 3rd letter
37247	Chronic obstructive pulmonary disease NOS
37371	Chronic obstructive pulmonary disease monitoring due
44525	Obstructive chronic bronchitis NOS
45998	Chronic obstructive pulmonary disease monitoring by doctor
93568	Very severe chronic obstructive pulmonary disease
12166	Other specified chronic obstructive airways disease

For peer review only

38074 Chronic obstructive pulmonary disease monitor phone invite
42258 Chronic obstructive pulmonary disease monitoring verb invite
42313 Health education - chronic obstructive pulmonary disease
45770 Chronic obstructive pulmonary disease disturbs sleep
45777 Chronic obstructive pulmonary disease clini management plan

COPD medication codes

prodcode	productname
8	salbutamol 100micrograms/dose inhaler
17	salbutamol 100micrograms/dose inhaler cfc free
31	ventolin 100microgram/inhalation inhalation powder (glaxo wellcome uk ltd)
38	beclometasone 100micrograms/dose inhaler
99	becotide 100 inhaler (glaxosmithkline uk ltd)
218	aminophylline 100 mg cap
235	bricanyl 250micrograms/dose inhaler (astrazeneca uk ltd)
273	theophylline 200 mg cap
282	salbutamol 2mg/5ml oral solution sugar free
454	pulmicort 200microgram inhaler (astrazeneca uk ltd)
465	salmeterol 25micrograms/dose inhaler
510	ventolin 5mg/ml respirator solution (glaxosmithkline uk ltd)
549	serevent 25micrograms/dose inhaler (glaxosmithkline uk ltd)
555	aminophylline 225mg modified-release tablets
590	phyllocontin continus 225mg tablets (napp pharmaceuticals ltd)
638	seretide 250 accuhaler (glaxosmithkline uk ltd)
665	seretide 100 accuhaler (glaxosmithkline uk ltd)
674	ventolin 2.5mg nebules (glaxosmithkline uk ltd)
696	salbutamol 8mg modified-release capsules
719	salmeterol 50micrograms/dose dry powder inhaler
746	tiotropium 18 microgram capsule
752	carbocisteine 375mg capsules
856	ventolin 2mg/5ml syrup (glaxosmithkline uk ltd)
860	salbutamol 4mg tablets
862	salbulin inhalation powder (3m health care ltd)
863	slo-phyllin 125mg capsule (lipha pharmaceuticals ltd)
879	theophylline 125mg modified-release capsules
880	theophylline 60mg modified-release capsules
881	salbutamol 2mg tablets
882	salbutamol 200microgram inhalation powder capsules
883	becodisks 200microgram disc (allen & hanburys ltd)
895	beclazone 100 easi-breathe inhaler (teva uk ltd)
896	becotide easi-breathe 100microgram/actuation pressurised inhalation (allen & hanburys ltd)
898	ventolin evohaler 100 100microgram/inhalation pressurised inhalation (glaxo wellcome uk ltd)
907	bricanyl turbohaler 500 500microgram turbohaler (astrazeneca uk ltd)
908	pulmicort 400 turbohaler (astrazeneca uk ltd)
909	budesonide 200micrograms/dose inhaler
910	serevent diskhaler 50microgram inhalation powder (glaxo wellcome uk ltd)
911	flixotide accuhaler 250 250microgram/inhalation inhalation powder (allen & hanburys ltd)
947	budesonide 50micrograms/actuation refill canister
956	pulmicort 200 turbohaler (astrazeneca uk ltd)

For peer review only

957 salamol easi-breathe 100microgram/actuation pressurised inhalation (ivax pharmaceuticals uk ltd)
958 ventolin easi-breathe 100microgram/actuation pressurised inhalation (allen & hanburys ltd)
959 budesonide 50micrograms/dose inhaler
960 pulmicort 100 turbohaler (astrazeneca uk ltd)
987 ventolin 4mg tablet (allen & hanburys ltd)
1093 salamol 100microgram/actuation inhalation powder (ivax pharmaceuticals uk ltd)
1097 slo-phyllin 60mg capsule (lipha pharmaceuticals ltd)
1100 beclazone 100 inhaler (teva uk ltd)
1236 becloforte 250micrograms/dose inhaler (glaxosmithkline uk ltd)
1242 beclometasone 250micrograms/dose inhaler
1243 beclazone 250 easi-breathe inhaler (teva uk ltd)
1258 becotide 200 inhaler (glaxosmithkline uk ltd)
1259 beclometasone 200micrograms/dose inhaler
1269 becotide 50microgram/ml nebuliser liquid (allen & hanburys ltd)
1346 salbutamol 0.05mg/ml injection
1406 becotide 50 inhaler (glaxosmithkline uk ltd)
1409 ipratropium bromide 20micrograms/dose inhaler
1410 ipratropium bromide 0.25mg/ml
1411 ipratropium bromide 250micrograms/ml
1412 flixotide 250microgram/actuation inhalation powder (allen & hanburys ltd)
1414 salamol 5mg/2.5ml nebuliser liquid steri-neb unit dose vials (teva uk ltd)
1415 steri-neb ipratropium 250microgram/ml nebuliser liquid (ivax pharmaceuticals uk ltd)
1423 uniphyllin continus 200mg tablets (napp pharmaceuticals ltd)
1424 flixotide 250microgram disc (allen & hanburys ltd)
1426 flixotide 500microgram disc (allen & hanburys ltd)
1518 flixotide 50microgram/actuation inhalation powder (allen & hanburys ltd)
1537 becotide 200microgram rotacaps (glaxosmithkline uk ltd)
1551 beclazone 250 inhaler (teva uk ltd)
1552 becloforte easi-breathe 250microgram/actuation pressurised inhalation (allen & hanburys ltd)
1619 terbutaline 500micrograms/dose dry powder inhaler
1620 terbutaline 250micrograms/dose inhaler
1628 terbutaline 250micrograms/actuation refill canister
1630 salbutamol 2.5mg/2.5ml nebuliser liquid unit dose vials
1635 salbuvent 2mg/5ml oral solution (pharmacia ltd)
1642 budesonide 400micrograms/dose dry powder inhaler
1676 flixotide 125microgram/actuation inhalation powder (allen & hanburys ltd)
1680 pulmicort ls 50micrograms/dose inhaler (astrazeneca uk ltd)
1698 salbutamol 100micrograms/dose breath actuated inhaler
1711 salbutamol 5mg/2.5ml nebuliser liquid unit dose vials
1725 beclazone 50 easi-breathe inhaler (teva uk ltd)
1727 becotide easi-breathe 50microgram/actuation pressurised inhalation (allen & hanburys ltd)
1734 beclometasone 100micrograms/dose breath actuated inhaler
1741 salbutamol 100micrograms/dose breath actuated inhaler cfc free
1833 theophylline 200mg modified-release tablets
1834 theophylline 400mg modified-release tablets
1861 aerobec 100 autohaler (meda pharmaceuticals ltd)
1882 ventodisks 200microgram/blister disc (allen & hanburys ltd)
1885 beclazone 200 inhaler (teva uk ltd)
1950 ventodisks 400microgram/blister disc (allen & hanburys ltd)
1951 becodisks 400microgram disc (allen & hanburys ltd)
1952 ventolin 400microgram rotacaps (glaxosmithkline uk ltd)

1956	pulmicort 1mg respules (astrazeneca uk ltd)
1957	ventolin 5mg nebules (glaxosmithkline uk ltd)
1959	pulmicort 0.5mg respules (astrazeneca uk ltd)
1972	oxygen bp size af lightweight gas 1360 litres
1974	oxis 12 turbohaler (astrazeneca uk ltd)
1975	oxis 6 turbohaler (astrazeneca uk ltd)
2044	prednisone 2.5 mg tab
2092	budesonide 200micrograms/dose dry powder inhaler
2124	pulmicort refil 200 mcg inh
2125	pulmicort 200microgram refill canister (astrazeneca uk ltd)
2147	theophylline 250mg modified-release capsules
2148	beclometasone 400microgram disc
2149	steri-neb salamol 2.5 mg inh
2152	ipratropium bromide with salbutamol 20mcg + 100mcg
2159	aerobec 50 autohaler (meda pharmaceuticals ltd)
2160	beclometasone 50micrograms/dose breath actuated inhaler
2224	serevent 50micrograms/dose accuhaler (glaxosmithkline uk ltd)
2229	becodisks 100microgram disc (allen & hanburys ltd)
2282	fluticasone 500micrograms/dose dry powder inhaler
2335	qvar 100 inhaler (teva uk ltd)
2368	prednisolone 2.5mg tablet
2390	prednisolone e/c 1 mg tab
2395	salbutamol 2 mg/5ml syr
2437	oxitropium bromide 100micrograms/dose inhaler
2440	flixotide accuhaler 500 500microgram/inhalation inhalation powder (allen & hanburys ltd)
2510	oxygen bp gas 1280 litres
2600	beclometasone 250micrograms/dose breath actuated inhaler
2655	airomir 100micrograms/dose inhaler (teva uk ltd)
2723	fluticasone 25micrograms/dose inhaler
2757	slo-phyllin 250mg capsule (lipha pharmaceuticals ltd)
2758	bricanyl refill canister (astrazeneca uk ltd)
2799	prednisolone 10 mg tab
2850	salbutamol 400microgram inhalation powder capsules
2851	ventolin 200microgram rotacaps (glaxosmithkline uk ltd)
2869	salbutamol 8mg modified-release tablets
2892	becloforte 400microgram disks (glaxosmithkline uk ltd)
2893	beclometasone 200micrograms disc
2949	prednisone 5mg tablets
2951	fluticasone 250microgram/actuation pressurised inhalation
2978	salbutamol 200micrograms/dose dry powder inhaler
2992	beclazone 50 inhaler (teva uk ltd)
2995	nuelin sa 175mg tablets (meda pharmaceuticals ltd)
3018	beclometasone 50micrograms/dose inhaler
3059	prednisolone 50 mg tab
3065	bextasol inhalation powder (allen & hanburys ltd)
3075	becotide 400microgram rotacaps (glaxosmithkline uk ltd)
3119	becloforte integra 250microgram/actuation inhaler with compact spacer (glaxo laboratories ltd)
3150	beclometasone 100micrograms/actuation extrafine particle cfc free inhaler
3163	salbutamol 200micrograms disc
3188	pulmicort complete 50 mcg inh
3189	salbuvent inh inh
3220	qvar 50 autohaler (teva uk ltd)
3254	salbulin 4mg tablet (3m health care ltd)

For peer review only

3289 flixotide 25micrograms/dose inhaler (glaxosmithkline uk ltd)
3297 salmeterol 50micrograms disc
3322 aerolin inh 400 100 mcg aer
3345 sintisone tablet (pharmacia ltd)
3347 oxygen bp size f gas 1360 litres
3363 becloforte 400microgram disks with diskhaler (glaxosmithkline uk ltd)
3388 theophylline 175mg modified-release tablets
3442 pulmicort complete 200 mcg inh
3443 salbutamol 100microgram/inhalation spacehaler (celltech pharma europe ltd)
3534 bricanyl 5mg tablets (astrazeneca uk ltd)
3546 qvar 50 inhaler (teva uk ltd)
3556 beclometasone 50micrograms with salbutamol 100micrograms/inhalation inhaler
3557 prednisone 1mg tablets
3570 budesonide 200micrograms/actuation refill canister
3584 bricanyl 1.5mg/5ml syrup (astrazeneca uk ltd)
3666 seretide 500 accuhaler (glaxosmithkline uk ltd)
3743 filair 50 inhaler (meda pharmaceuticals ltd)
3753 flixotide diskhaler-community pack 250 mcg
3758 pulmadil inhalation powder (3m health care ltd)
3763 terbutaline respules inh
3764 bricanyl respules (5mg/2ml) 2.5 mg/ml inh
3786 fenoterol 100micrograms/dose / ipratropium 40micrograms/dose inhaler
3838 salbutamol 400mcg/beclometh.100mcg r/cap inh
3927 filair 100 inhaler (meda pharmaceuticals ltd)
3947 becotide 100microgram rotacaps (glaxosmithkline uk ltd)
3988 flixotide diskhaler-community pack 100 mcg
3989 flixotide 100microgram disc (allen & hanburys ltd)
3993 filair forte 250micrograms/dose inhaler (meda pharmaceuticals ltd)
3994 salbutamol 4mg modified-release tablets
4055 salbulin 2mg/5ml oral solution (3m health care ltd)
4131 fluticasone 100microgram disc
4132 fluticasone 125microgram/actuation pressurised inhalation
4165 zithromax 250mg capsules (pfizer ltd)
4171 ventolin 2mg tablet (allen & hanburys ltd)
4222 bricanyl 10mg/ml respirator solution (astrazeneca uk ltd)
4268 ipratropium bromide 40micrograms/dose inhaler
4365 beclometasone 100micrograms disc
4412 oxygen bp size g gas 3400 litres
4413 qvar 100 autohaler (teva uk ltd)
4497 ventolin accuhaler 200 200microgram/actuation inhalation powder (glaxo wellcome uk ltd)
4499 aerobec 250microgram/actuation pressurised inhalation (meda pharmaceuticals ltd)
4514 aminophylline 350mg modified-release tablets
4538 oxygen bp size pd gas 300 litres
4541 bricanyl sa 7.5mg tablets (astrazeneca uk ltd)
4545 pulmicort ls 50microgram refill canister (astrazeneca uk ltd)
4593 theophylline 125mg tablets
4601 asmabec 100 clickhaler (focus pharmaceuticals ltd)
4634 salamol 2.5mg/2.5ml nebuliser liquid steri-neb unit dose vials (teva uk ltd)
4640 bricanyl 5mg/2ml nebuliser liquid (astrazeneca uk ltd)
4665 salbulin 100micrograms/dose inhaler (3m health care ltd)
4688 fluticasone 50microgram/actuation pressurised inhalation
4759 beclometasone 100microgram inhalation powder capsules
4791 oxygen bp size c gas 170 litres
4801 budesonide 500micrograms/2ml nebuliser liquid unit dose vials

For peer review only

4803 beclazone 250microgram/actuation inhalation powder (actavis uk ltd)
4842 fenoterol 100microgram/actuation inhaler
4908 ventolin rotahaler (glaxosmithkline uk ltd)
4926 flixotide accuhaler 100 100microgram/inhalation inhalation powder (allen & hanburys ltd)
4942 budesonide 1mg/2ml nebuliser liquid unit dose vials
5057 azithromycin 200mg/5ml oral suspension
5116 azithromycin 250mg capsules
5143 seretide 50 evohaler (glaxosmithkline uk ltd)
5161 seretide 125 evohaler (glaxosmithkline uk ltd)
5170 salamol 100micrograms/dose inhaler cfc free (teva uk ltd)
5172 seretide 250 evohaler (glaxosmithkline uk ltd)
5185 fenoterol 200micrograms/dose inhaler
5223 fluticasone 50micrograms/dose inhaler cfc free
5261 nuelin sa 250 tablets (meda pharmaceuticals ltd)
5308 terbutaline 5mg/2ml nebuliser liquid unit dose vials
5309 flixotide 50micrograms/dose evohaler (glaxosmithkline uk ltd)
5335 zithromax 500mg tablets (pfizer ltd)
5453 uniphyllin continus 400mg tablets (napp pharmaceuticals ltd)
5516 salamol 100micrograms/dose easi-breathe inhaler (teva uk ltd)
5521 beclometasone 200micrograms/dose dry powder inhaler
5522 beclometasone 100micrograms/dose dry powder inhaler
5551 flixotide 0.5mg/2ml nebules (glaxosmithkline uk ltd)
5558 salmeterol 50micrograms with fluticasone 500micrograms cfc free inhaler
5580 flixotide accuhaler 50 50microgram/inhalation inhalation powder (allen & hanburys ltd)
5584 oxygen cylinders size pd (boc ltd)
5683 flixotide 250micrograms/dose evohaler (glaxosmithkline uk ltd)
5718 flixotide 125micrograms/dose evohaler (glaxosmithkline uk ltd)
5740 airomir 100micrograms/dose autohaler (teva uk ltd)
5753 salbutamol 400micrograms disc
5773 oxygen bp size dd gas 460 litres
5780 oxygen cylinders size af (boc ltd)
5804 beclometasone 250micrograms/dose dry powder inhaler
5822 fluticasone 250micrograms/dose inhaler cfc free
5837 salamol steri-neb 5mg/2.5ml nebuliser liquid (numark management ltd)
5864 salmeterol 25micrograms with fluticasone 250micrograms cfc free inhaler
5885 fluticasone 100micrograms/dose dry powder inhaler
5889 salamol 100microgram/inhalation inhalation powder (kent pharmaceuticals ltd)
5898 salamol steri-neb 2.5mg/2.5ml nebuliser liquid (numark management ltd)
5941 uniphyllin continus 300mg tablets (napp pharmaceuticals ltd)
5942 salmeterol 50micrograms with fluticasone 250micrograms cfc free inhaler
5975 fluticasone 125micrograms/dose inhaler cfc free
5976 oxygen bp size dd 460 litre inhalation gas (boc ltd)
5992 beclometasone 50micrograms/dose dry powder inhaler
6081 ipratropium bromide 20micrograms/dose breath actuated inhaler
6276 carbocisteine 250mg/5ml oral solution
6315 slo-phyllin 250mg capsules (merck serono ltd)
6325 symbicort 200/6 turbohaler (astrazeneca uk ltd)
6419 oxygen cylinders size f (boc ltd)
6420 oxygen bp size d gas 340 litres
6423 oxygen bp with integral headset 1360 litre inhalation gas (boc ltd)
6462 salbutamol 95micrograms/dose dry powder inhaler
6522 ipratropium bromide 20micrograms/dose inhaler cfc free
6526 formoterol 12microgram inhalation powder capsules with device

For peer review only

6556	oxygen cylinders size c (boc ltd)
6569	salmeterol 25micrograms with fluticasone 125micrograms cfc free inhaler
6616	salmeterol 25micrograms with fluticasone 50micrograms cfc free inhaler
6619	oxygen bp size cd gas 460 litres
6719	ipratropium bromide 500micrograms/2ml nebuliser liquid unit dose vials
6746	budesonide 400micrograms/dose / formoterol 12micrograms/dose dry powder inhaler
6758	ipratropium 250micrograms/1ml nebuliser liquid steri-neb unit dose vials (teva uk ltd)
6768	oxygen bp size cd 460 litre inhalation gas (boc ltd)
6772	ipratropium bromide 250micrograms/1ml nebuliser liquid unit dose vials
6780	symbicort 400/12 turbohaler (astrazeneca uk ltd)
6796	budesonide 200micrograms/dose / formoterol 6micrograms/dose dry powder inhaler
6802	mucodyne 375mg capsules (sanofi)
6848	oxygen cylinders size dd with integral headset 2 and 4litres/minute flow rate (boc ltd)
6920	mecysteine 100mg gastro-resistant tablets
6938	salmeterol 50micrograms with fluticasone 100micrograms dry powder inhaler
6976	oxygen bp with integral headset gas 430 litres
6988	aminophylline hydrate 100mg modified-release tablets
7013	symbicort 100/6 turbohaler (astrazeneca uk ltd)
7017	salbutamol 100micrograms/dose dry powder inhaler
7031	oxygen bp size e gas 680 litres
7042	oxygen cylinders size e (boc ltd)
7133	formoterol 12micrograms/dose dry powder inhaler
7192	bambuterol 10mg tablets
7239	oxygen cylinders size b10s (air products plc)
7268	serevent 25micrograms/dose evohaler (glaxosmithkline uk ltd)
7270	salmeterol 25micrograms/dose inhaler cfc free
7452	ventolin .25 mg inj
7584	prednisolone 4 mg tab
7602	fluticasone 50microgram disc
7638	fluticasone 250microgram disc
7653	beclometasone 400microgram inhalation powder capsules
7710	prednisolone 15 mg tab
7711	terbutaline 250micrograms/dose inhaler with spacer
7724	betamethasone valerate 100micrograms/actuation inhaler
7730	theo-dur 300mg tablets (astrazeneca uk ltd)
7731	theo-dur 200mg tablets (astrazeneca uk ltd)
7732	theophylline 300mg modified-release tablets
7733	theophylline 250mg modified-release tablets
7788	budesonide 100micrograms/dose dry powder inhaler
7832	choline theophyllinate 200mg tablets
7841	nuelin 125mg tablets (3m health care ltd)
7891	fluticasone 500microgram disc
7934	prednisone 30 mg tab
7935	maxivent 100microgram/inhalation inhalation powder (ashbourne pharmaceuticals ltd)
7948	fluticasone 250micrograms/dose dry powder inhaler
7953	terbutaline 1.5mg/5ml oral solution sugar free
7954	bricanyl 250micrograms/dose spacer inhaler (astrazeneca uk ltd)
7964	beclometasone 50micrograms/ml nebuliser suspension
7965	salbutamol 5mg/ml nebuliser liquid
8012	exirel 15mg capsule (3m health care ltd)
8056	aminophylline 100mg tablets
8057	aminophylline 100mg modified-release tablets
8111	becloforte vm 250microgram/actuation vm pack (allen & hanburys ltd)
8183	oxygen bp gas 640 litres

For peer review only

8251	pulmicort refil 50 mg inh
8252	pirbuterol 15mg capsule
8267	sodium cromoglicate 1mg/dose / salbutamol 100micrograms/dose inhaler
8333	ipratropium bromide 40microgram inhalation powder capsules
8339	fenoterol hydrobromide complete unit inh
8429	ventolin i/v 5 mg inj
8433	budesonide 100micrograms/actuation inhaler
8450	flixotide diskhaler-community pack 50 mcg
8504	exirel 15 mg tab
8505	oxygen bp gas 6400 litres
8522	terbutaline 7.5mg modified-release tablets
8572	rimiterol inhaler
8635	flixotide 50microgram disc (allen & hanburys ltd)
8636	ventolin s/r 8 mg spa
8676	terbutaline 10mg/ml nebuliser liquid
8757	oxygen bp gas 3200 litres
8806	phyllocontin continus 350mg tablet (napp pharmaceuticals ltd)
8955	theophylline 100 mg tab
9018	mucodyne 375mg capsule (aventis pharma)
9092	theophylline 350mg modified release tablets
9164	fluticasone 50micrograms/dose dry powder inhaler
9233	beclometasone 200microgram inhalation powder capsules
9270	ipratropium bromide with fenoterol hydrobromide 500micrograms + 1.25mg/4ml
9384	salbutamol 4mg modified-release capsules
9477	asmabec 100microgram/actuation spacehaler (celltech pharma europe ltd)
9571	beclometasone 250micrograms/actuation vortex inhaler
9577	asmabec 50 clickhaler (focus pharmaceuticals ltd)
9599	beclazone 50microgram/actuation inhalation powder (actavis uk ltd)
9642	mucodyne 250mg/5ml oral solution (aventis pharma)
9651	asmasal 100microgram/inhalation spacehaler (celltech pharma europe ltd)
9658	oxitropium bromide 100micrograms/dose breath actuated inhaler
9711	formoterol 6micrograms/dose dry powder inhaler
9727	prednisolone 50mg tablets
9805	salbutamol 5mg/50ml solution for infusion vials
9889	oxygen cylinders size g (boc ltd)
9895	oxygen bp size df with integral headset gas 1360 litres
9906	mucodyne 250mg/5ml syrup (sanofi)
9921	beclometasone 100micrograms/dose breath actuated inhaler cfc free
9943	visclair 100mg gastro-resistant tablets (ranbaxy (uk) ltd)
10043	oxygen composite cylinders size b10c with integral headset (air products plc)
10053	oxygen connection cubing 1.8m
10090	beclometasone 50micrograms/actuation extrafine particle cfc free inhaler
10218	budesonide 100micrograms/dose / formoterol 6micrograms/dose dry powder inhaler
10321	budesonide 400microgram inhalation powder capsules
10331	nuelin 60mg/5ml liquid (3m health care ltd)
10353	salbuvent rondo
10360	aerocrom inhaler (castlemead healthcare ltd)
10407	phyllocontin paediatric continus 100mg tablets (napp pharmaceuticals ltd)
10433	theophylline 60mg/5ml oral solution
10458	ventolin cr 4mg tablet (allen & hanburys ltd)
10561	aminophylline 250mg/ml injection
10723	theophylline 125mg/5ml syrup
10744	theophylline 80 mg eli
10808	mucodyne paediatric 125mg/5ml syrup (sanofi)
10825	terbutaline 5mg tablets

10831 biophylline 125mg/5ml oral solution (lorex synthelabo ltd)
10858 pulmadil auto inhalation powder (3m health care ltd)
10958 salbutamol .25 mg inj
10968 foradil 12microgram inhalation powder capsules with device (novartis pharmaceuticals uk ltd)
11046 ipratropium bromide with salbutamol 500micrograms + 2.5mg/2.5ml
11149 betnelan 500microgram tablets (focus pharmaceuticals ltd)
11198 beclometasons 50 micrograms/actuation vortex inhaler
11307 salbutamol 100micrograms/dose / beclometasone 50micrograms/dose inhaler
11410 fluticasone 500micrograms/dose / salmeterol 50micrograms/dose dry powder inhaler
11478 fluticasone 2mg/2ml nebuliser liquid unit dose vials
11497 beclometasone 400micrograms/dose dry powder inhaler
11588 fluticasone 125micrograms/dose / salmeterol 25micrograms/dose inhaler cfc free
11618 fluticasone 250micrograms/dose / salmeterol 25micrograms/dose inhaler cfc free
11659 oxygen bp size dd with integral headset gas 460 litres
11719 slo-phyllin 60mg capsules (merck serono ltd)
11732 beclometasone 50micrograms/dose breath actuated inhaler cfc free
11779 ipratropium bromide 40microgram inhalation powder capsules with device
11993 pro-vent 300mg capsule (wellcome medical division)
12042 ventolin cr 8mg tablet (allen & hanburys ltd)
12144 bambuterol 20mg tablets
12240 theophylline 300mg modified release capsules
12463 pirbuterol 15 mg tab
12479 aerolin inh auto refil 100 mcg aer
12486 bronchodil 500microgram/dose inhalation powder (viatris pharmaceuticals ltd)
12529 fabrol 200mg granules (novartis consumer health uk ltd)
12563 exirel inhalation powder (3m health care ltd)
12699 pecram 225mg modified-release tablet (novartis consumer health uk ltd)
12808 fenoterol 100micrograms/dose / ipratropium bromide 40micrograms/dose breath actuated inhaler
12822 salbutamol 2.5mg with ipratropium bromide 500micrograms/2.5ml unit dose nebuilser solution
12909 salbutamol 100micrograms/dose / ipratropium 20micrograms/dose inhaler
12994 fluticasone 50micrograms/dose / salmeterol 25micrograms/dose inhaler cfc free
13037 pulvinal beclometasone dipropionate 200micrograms/dose dry powder inhaler (chiesi ltd)
13038 pulvinal salbutamol 200micrograms/dose dry powder inhaler (chiesi ltd)
13040 fluticasone 250micrograms/dose / salmeterol 50micrograms/dose dry powder inhaler
13181 easyhaler salbutamol sulphate 100micrograms/dose dry powder inhaler (orion pharma (uk) ltd)
13206 oxygen bp with integral headset gas 300 litres
13273 fluticasone 100micrograms/dose / salmeterol 50micrograms/dose dry powder inhaler
13290 clenil modulite 100micrograms/dose inhaler (chiesi ltd)
13307 bricanyl 500microgram/ml injection (astrazeneca uk ltd)
13522 prednisolone 2 mg tab
13529 amnivent-225 sr tablets (ashbourne pharmaceuticals ltd)
13575 bambec 20mg tablets (astrazeneca uk ltd)
13615 prednisone 10 mg tab
13757 tropiovent steripoule 250microgram/ml nebuliser liquid (ashbourne pharmaceuticals ltd)
13815 beclazone 100microgram/actuation inhalation powder (actavis uk ltd)
13996 salamol 100microgram/inhalation inhalation powder (sandoz ltd)
14294 qvar 50micrograms/dose easi-breathe inhaler (teva uk ltd)
14306 formoterol 12micrograms/dose inhaler cfc free
14321 beclometasone 200micrograms/dose inhaler cfc free

For peer review only

14482 bricanyl 2.5 mg inj
14483 terbutaline 500micrograms/ml injection
14514 zithromax 200mg/5ml oral suspension (pfizer ltd)
14524 bdp 250microgram/actuation spacehaler (celltech pharma europe ltd)
14525 salbutamol 100micrograms/inhalation vortex inhaler
14527 bambec 10mg tablets (astrazeneca uk ltd)
14561 salbutamol 400microgram / beclometasone 200microgram inhalation powder capsules
14567 asmabec 250 clickhaler (focus pharmaceuticals ltd)
14590 asmabec 250microgram/actuation spacehaler (celltech pharma europe ltd)
14700 budesonide 400micrograms/actuation inhaler
14736 pulvinal beclometasone dipropionate 400micrograms/dose dry powder inhaler (chiesi ltd)
14739 norphyllin sr 225mg tablets (teva uk ltd)
14757 pulvinal beclometasone dipropionate 100micrograms/dose dry powder inhaler (chiesi ltd)
14991 aminophylline 250mg/10ml injection
15075 bronchodil 20mg tablet (viatris pharmaceuticals ltd)
15165 reproterol 500micrograms/dose inhaler
15214 oxygen bp size pd 300 litre inhalation gas (medigas ltd)
15281 oxygen cylinders size pa2 with integral headset (air products plc)
15284 slo-phyllin 125mg capsules (merck serono ltd)
15301 carbocisteine 125mg/5ml oral solution
15326 beclometasone 100micrograms/dose inhaler cfc free
15365 theophylline 10mg/5ml sf elixir
15409 theophylline 3 mg sol
15441 fenoterol hydrobromide .5 % sol
15613 salbutamol 500micrograms/1ml solution for injection ampoules
15706 beclometasone 100 micrograms/actuation vortex inhaler
15979 oxygen bp with integral headset 300 litre inhalation gas (boc ltd)
16054 budesonide 200micrograms/actuation breath actuated powder inhaler
16124 oxygen cylinders (medigas ltd)
16148 clenil modulite 250micrograms/dose inhaler (chiesi ltd)
16151 clenil modulite 200micrograms/dose inhaler (chiesi ltd)
16158 clenil modulite 50micrograms/dose inhaler (chiesi ltd)
16236 pirbuterol acetate inhaler
16305 flixotide 2mg/2ml nebules (glaxosmithkline uk ltd)
16523 oxygen cylinders size cd with integral headset 0-15litres/minute flow rate (boc ltd)
16577 easyhaler salbutamol sulphate 200micrograms/dose dry powder inhaler (orion pharma (uk) ltd)
16584 beclometasone 50micrograms/dose inhaler cfc free
16625 ventide rotacaps (glaxosmithkline uk ltd)
16724 prednisone 50 mg tab
16994 aminophylline hydrate 350mg modified-release tablets
17002 aminophylline hydrate 225mg modified-release tablets
17005 oxygen bp size cd with integral headset gas 460 litres
17140 aminophylline 200mg tablets
17185 ventolin 500micrograms/1ml solution for injection ampoules (glaxosmithkline uk ltd)
17465 fluticasone 500micrograms/2ml nebuliser liquid unit dose vials
17644 oxygen bp with integral headset gas 2122 litres
17654 easyhaler beclometasone 200micrograms/dose dry powder inhaler (orion pharma (uk) ltd)
17670 easyhaler budesonide 100micrograms/dose dry powder inhaler (orion pharma (uk) ltd)
17696 ventmax sr 4mg capsules (chiesi ltd)

For peer review only

17874 monovent 1.5mg/5ml oral solution (lagap)
17901 bricanyl nebule 2.5 ml
18140 respontin 500micrograms/2ml nebules (glaxosmithkline uk ltd)
18288 choline theophyllinate 100mg tablets
18299 fenoterol 1.25mg/4ml / ipratropium 500micrograms/4ml nebuliser liquid unit dose vials
18314 aerocrom syncroner with spacer (castlemead healthcare ltd)
18394 bdp 50microgram/actuation spacehaler (celltech pharma europe ltd)
18421 respontin nebules 250microgram/ml nebuliser liquid (glaxo wellcome uk ltd)
18456 salbutamol 200microgram / beclometasone 100microgram inhalation powder capsules
18484 ventide paediatric rotacaps (glaxosmithkline uk ltd)
18537 budesonide 200microgram inhalation powder capsules
18622 salbulin 2mg tablet (3m health care ltd)
18848 qvar 100micrograms/dose easi-breathe inhaler (teva uk ltd)
18937 sabidal sr 270 270 mg tab
18968 salbutamol 5mg/5ml solution for infusion ampoules
18988 choline theophyllinate 62.5mg/5ml oral solution
19031 bdp 100microgram/actuation spacehaler (celltech pharma europe ltd)
19121 beclometasone 100micrograms with salbutamol 200micrograms inhalation capsules
19376 beclometasone 200micrograms with salbutamol 400micrograms inhalation capsules
19389 asmabec 50microgram/actuation spacehaler (celltech pharma europe ltd)
19401 beclometasone 250micrograms/actuation inhaler and compact spacer
19563 becotide for nebuliser
19642 ventolin nebules
19649 ventolin rotahaler
19653 ventolin respirator
19726 ventolin s/r
19732 cobutolin inh
19735 uniphyllin continus
19736 becotide susp for nebulisation
19737 atrovent nebuliser solution (1ml vial)
19799 tulobuterol 2mg
19805 atrovent
20095 precortisyl forte 25mg tablet (aventis pharma)
20225 aminophylline 500 mg inj
20670 prednisolone e/c
20675 salbutamol rotahaler complete unit
20680 aerolin autohaler
20707 becotide 100
20720 atrovent forte
20763 becloforte
20781 salbutamol u.dose nebulising 2.5mg/2.5ml
20803 ipratropium bromide nebuliser solution
20812 pulmicort refill
20825 spacehaler bdp 250microgram/actuation spacehaler (celltech pharma europe ltd)
20838 salbuvent 2mg tablet (pharmacia ltd)
21005 beclometasone 250micrograms/dose inhaler cfc free
21102 salbutamol 2mg/5ml oral solution (lagap)
21330 oxygen composite cylinders size if2 with integral headset (medigas ltd)
21331 oxygen composite cylinders with integral headset (air products plc)
21402 oxygen cylinders size pd (air products plc)
21482 beclometasone 100micrograms/dose inhaler (generics (uk) ltd)
21769 lasma 300mg tablet (pharmax ltd)
21833 decortisyl 5mg tablet (roussel laboratories ltd)

For peer review only

21859 asmaven 100microgram inhalation powder (berk pharmaceuticals ltd)
22080 aminophylline 20 ml inj
22225 beclomethasone /salbutamol
22313 ventmax sr 8mg capsules (chiesi ltd)
22430 spacehaler salbutamol 100microgram/inhalation spacehaler (celltech pharma europe ltd)
22467 salbutamol respirator soln
22512 salbutamol inhaler
22550 duovent
22661 pirbuterol 10mg capsule
22663 respacal 2mg tablet (ucb pharma ltd)
22669 choline theophyllinate 270 mg tab
22790 reproterol 10mg/ml respirator solution
22828 carbocisteine 750mg/5ml forte oral solution
23269 maxivent 2.5mg/2.5ml nebuliser liquid unit dose steripoule vials (ashbourne pharmaceuticals ltd)
23337 oxygen cylinder f size 1280 litres
23512 precortisyl 5mg tablet (hoechst marion roussel)
23567 respontin 250micrograms/1ml nebules (glaxosmithkline uk ltd)
23572 aminophylline sr 225mg modified-release tablet (ivax pharmaceuticals uk ltd)
23675 pulmicort l.s. refil
23688 ventolin rotacaps
23709 ipratropium 500micrograms/2ml nebuliser liquid steri-neb unit dose vials (teva uk ltd)
23741 novolizer budesonide 200microgram/actuation pressurised inhalation (meda pharmaceuticals ltd)
23787 exirel 10mg capsule (3m health care ltd)
23905 oxygen cylinders size d (boc ltd)
23961 ipratropium bromide 250microgram/ml inhalation vapour (galen ltd)
24219 becotide rotacaps
24380 sodium cromoglicate 1mg/dose / salbutamol 100micrograms/dose inhaler with spacer
24418 biophylline 350mg tablet (lorex synthelabo ltd)
24456 carbocisteine 375mg tablets
24645 ventolin 5mg/5ml solution for infusion ampoules (glaxosmithkline uk ltd)
24660 betamethasone valerate
24674 biophylline 500mg tablet (lorex synthelabo ltd)
24716 prednisolone e/c
24898 spacehaler bdp 100microgram/actuation spacehaler (celltech pharma europe ltd)
25020 ipratropium bromide (forte)
25073 salbutamol
25093 theophylline s/r
25204 beclometasone 100micrograms/dose inhaler (a a h pharmaceuticals ltd)
25218 salbutamol cfc/free b/a
25272 precortisyl 1mg tablet (hoechst marion roussel)
25339 maxivent 5mg/2.5ml nebuliser liquid unit dose steripoule vials (ashbourne pharmaceuticals ltd)
25784 atimos modulite 12micrograms/dose inhaler (chiesi ltd)
25820 bronchodil 10mg/5ml oral solution (viatris pharmaceuticals ltd)
25821 exirel 7.5mg/5ml oral solution (3m health care ltd)
25829 pirbuterol 7.5mg/5ml oral solution
26063 beclometasone 100micrograms/dose inhaler (teva uk ltd)
26079 uniphyllin paediatric continus
26420 exirel 10 mg tab
26525 ventolin
26616 ipratropium bromide with fenoterol hydrobromide 0micrograms + 100micrograms/actuation

For peer review only

26665 pulmicort complete
26716 airomir autohaler cfc free b/a
26829 brelomax 2mg tablet (abbott laboratories ltd)
26873 cobutolin 2mg tablet (actavis uk ltd)
27040 phyllocontin continus
27188 easyhaler budesonide 200micrograms/dose dry powder inhaler (orion pharma (uk) ltd)
27301 bromhexine hcl 8mg tablets
27340 salbuvent 0.5mg/ml injection (pharmacia ltd)
27505 ipratropium bromide with fenoterol hydrobromide 40micrograms + 100micrograms/actuation
27525 becotide 50
27558 choledyl
27573 ventolin
27583 pulmicort
27679 beclometasone 100microgram/actuation pressurised inhalation (approved prescription services ltd)
27842 aminophylline 2 ml inj
27889 prednisolone
27915 fluticasone prop disk refill
27959 prednisolone
27962 deltastab 1mg tablet (waymade healthcare plc)
28073 beclometasone 250microgram/actuation pressurised inhalation (approved prescription services ltd)
28241 aminophylline 250mg/10ml solution for injection minijet pre-filled syringes (ucb pharma ltd)
28376 prednisolone 2.5mg gastro-resistant tablet (biorex laboratories ltd)
28508 salbutamol 100microgram/inhalation inhalation powder (ivax pharmaceuticals uk ltd)
28577 ventolin 50microgram/ml injection (allen & hanburys ltd)
28640 beclometasone 100microgram/actuation inhalation powder (actavis uk ltd)
28761 spacehaler bdp 50microgram/actuation spacehaler (celltech pharma europe ltd)
28859 deltastab 5mg tablet (waymade healthcare plc)
28881 salbutamol 2mg/5ml oral solution sugar free (a a h pharmaceuticals ltd)
29267 salbuvent 4mg tablet (pharmacia ltd)
29273 aminophylline 225mg modified-release tablet (hillcross pharmaceuticals ltd)
29325 beclometasone 250micrograms/dose inhaler (generics (uk) ltd)
30118 salbutamol 100micrograms/dose inhaler cfc free (teva uk ltd)
30204 salbutamol 200micrograms inahalation capsules
30210 beclometasone 250micrograms/dose inhaler (teva uk ltd)
30212 salbutamol cyclohaler
30229 ipratropium bromide 250microgram/ml nebuliser liquid (galen ltd)
30230 salbutamol 100micrograms/actuation breath actuated inhaler
30238 beclometasone 50microgram/actuation pressurised inhalation (approved prescription services ltd)
30240 aerolin autohaler 100microgram/actuation pressurised inhalation (3m health care ltd)
30390 deltastab 2 mg tab
30596 aminophylline 225mg modified-release tablet (actavis uk ltd)
30649 easyhaler budesonide 400micrograms/dose dry powder inhaler (orion pharma (uk) ltd)
30971 decortisyl 25 mg tab
31082 salbuvent 5mg/ml respirator solution (pharmacia ltd)
31290 salbulin cfc free
31327 prednisolone steaglate 6.65mg tablet
31758 uniphyllin continus
31774 beclometasone 50micrograms/dose inhaler (generics (uk) ltd)

For peer review only

31845	salapin 2mg/5ml syrup (pinewood healthcare)
31933	salbutamol 100micrograms/dose inhaler (a a h pharmaceuticals ltd)
32050	salbutamol 400 cyclocaps (teva uk ltd)
32102	salbutamol 4mg tablets (a a h pharmaceuticals ltd)
32461	choline theophyllinate 90 mg tab
32812	numotac 10mg tablet (3m health care ltd)
32874	beclometasone 50microgram/actuation inhalation powder (actavis uk ltd)
32893	theophylline 100mg/lysine 74mg mg tab
33089	salbutamol 100micrograms/dose inhaler (kent pharmaceuticals ltd)
33258	beclometasone 250micrograms/dose inhaler (a a h pharmaceuticals ltd)
33373	salbutamol 200 cyclocaps (teva uk ltd)
33588	salbutamol 100micrograms/dose inhaler (generics (uk) ltd)
33691	prednisolone 5mg gastro-resistant tablet (biorex laboratories ltd)
33817	salbutamol 100micrograms/dose inhaler cfc free (actavis uk ltd)
33849	beclometasone 100microgram/actuation inhalation powder (neo laboratories ltd)
33888	azithromycin 250mg tablets
33988	prednisolone 5mg tablet (co-pharma ltd)
33990	prednisolone 5mg tablet (ivax pharmaceuticals uk ltd)
34018	salbutamol 5mg/2.5ml nebuliser liquid (galen ltd)
34029	salbutamol 400micrograms inahalation capsules
34109	prednisolone 5 mg gastro-resistant tablet
34134	aerolin 400 100microgram/actuation inhalation powder (3m health care ltd)
34162	salbutamol 2.5mg/2.5ml nebuliser liquid (galen ltd)
34310	salbutamol 100micrograms/dose inhaler cfc free (a a h pharmaceuticals ltd)
34311	salbutamol 100microgram/inhalation inhalation powder (berk pharmaceuticals ltd)
34315	beclometasone 250microgram/actuation inhalation powder (actavis uk ltd)
34428	beclometasone 50microgram/actuation inhalation powder (neo laboratories ltd)
34618	salbutamol 2mg tablets (actavis uk ltd)
34619	salbutamol 100microgram/inhalation inhalation powder (kent pharmaceuticals ltd)
34631	prednisolone 1mg tablet (co-pharma ltd)
34702	salbutamol 100microgram/inhalation inhalation powder (c p pharmaceuticals ltd)
34739	beclometasone 50micrograms/dose inhaler (teva uk ltd)
34794	beclometasone 200micrograms/dose inhaler (a a h pharmaceuticals ltd)
34859	beclometasone 250microgram/actuation inhalation powder (neo laboratories ltd)
34914	prednisolone 1mg tablet (celltech pharma europe ltd)
34919	beclometasone 50micrograms/dose inhaler (a a h pharmaceuticals ltd)
34938	salbutamol 4mg tablets (actavis uk ltd)
35011	tiotropium bromide 18microgram inhalation powder capsules
35014	tiotropium bromide 18microgram inhalation powder capsules with device
35015	erdosteine 300mg capsules
35071	becodisks 200microgram (glaxosmithkline uk ltd)
35106	becodisks 100microgram with diskhaler (glaxosmithkline uk ltd)
35107	beclometasone 400microgram inhalation powder blisters with device
35113	beclometasone 200microgram inhalation powder blisters
35118	becodisks 400microgram with diskhaler (glaxosmithkline uk ltd)
35165	serevent 50microgram disks with diskhaler (glaxosmithkline uk ltd)
35178	erdotin 300mg capsules (galen ltd)
35225	flixotide 100microgram disks with diskhaler (glaxosmithkline uk ltd)
35288	beclometasone 400microgram inhalation powder blisters
35293	beclometasone 200microgram inhalation powder blisters with device
35299	becodisks 400microgram (glaxosmithkline uk ltd)
35374	flixotide 500microgram disks (glaxosmithkline uk ltd)
35392	flixotide 500microgram disks with diskhaler (glaxosmithkline uk ltd)
35408	becodisks 100microgram (glaxosmithkline uk ltd)
35430	becodisks 200microgram with diskhaler (glaxosmithkline uk ltd)

For peer review only

35461 flixotide 250microgram disks with diskhaler (glaxosmithkline uk ltd)
35503 salmeterol 50microgram inhalation powder blisters
35510 budesonide 200micrograms/dose dry powder inhalation cartridge with device
35522 bricanyl 500micrograms/1ml solution for injection ampoules (astrazeneca uk ltd)
35542 salmeterol 50microgram inhalation powder blisters with device
35557 ipramol nebuliser solution 2.5ml steri-neb unit dose vials (teva uk ltd)
35566 oxygen bp with set gas 1360 litres
35580 beclometasone 100microgram inhalation powder blisters with device
35602 budesonide 200micrograms/dose dry powder inhalation cartridge
35611 flixotide 250microgram disks (glaxosmithkline uk ltd)
35631 budelin novolizer 200micrograms/dose inhalation powder (meda pharmaceuticals ltd)
35638 fluticasone 100microgram inhalation powder blisters with device
35652 beclometasone 100microgram inhalation powder blisters
35700 fluticasone 500microgram inhalation powder blisters with device
35724 budelin novolizer 200micrograms/dose inhalation powder refill (meda pharmaceuticals ltd)
35725 formoterol easyhaler 12micrograms/dose dry powder inhaler (orion pharma (uk) ltd)
35744 bricanyl 2.5mg/5ml solution for injection ampoules (astrazeneca uk ltd)
35772 fluticasone 100microgram inhalation powder blisters
35825 serevent 50microgram disks (glaxosmithkline uk ltd)
35861 terbutaline 2.5mg/5ml solution for injection ampoules
35862 terbutaline 500micrograms/1ml solution for injection ampoules
35905 fluticasone 250microgram inhalation powder blisters
35986 flixotide 50microgram disks (glaxosmithkline uk ltd)
36021 fluticasone 50microgram inhalation powder blisters with device
36090 flixotide 100microgram disks (glaxosmithkline uk ltd)
36290 flixotide 50microgram disks with diskhaler (glaxosmithkline uk ltd)
36401 fluticasone 250microgram inhalation powder blisters with device
36462 fluticasone 500microgram inhalation powder blisters
36677 reproterol 10mg/5ml oral solution
36864 tiotropium bromide 2.5micrograms/dose solution for inhalation cartridge with device cfc free
37432 fostair 100micrograms/dose/6micrograms/dose inhaler (chiesi ltd)
37447 fluticasone 50microgram inhalation powder blisters
37470 beclometasone 100micrograms/dose / formoterol 6micrograms/dose inhaler cfc free
37612 terbutaline 5mg/2ml nebuliser liquid unit dose vials (galen ltd)
37791 ipratropium bromide 250microgram/ml
38079 salbutamol 100micrograms/dose dry powder inhalation cartridge with device
38097 salbutamol cyclocaps 200microgram inhalation powder (dupont pharmaceuticals ltd)
38120 theophylline 500mg modified release tablets
38136 salbulin novolizer 100micrograms/dose inhalation powder (meda pharmaceuticals ltd)
38214 salbutamol 100micrograms/dose dry powder inhalation cartridge
38226 salbulin novolizer 100micrograms/dose inhalation powder refill (meda pharmaceuticals ltd)
38407 prednisolone 20mg tablet
38416 salbutamol cyclocaps 400microgram inhalation powder (dupont pharmaceuticals ltd)
38419 terbutaline 1.5mg/5ml oral solution sugar free (a a h pharmaceuticals ltd)
39040 phyllocontin forte continus 350mg tablets (napp pharmaceuticals ltd)
39099 pulmicort 100micrograms/dose inhaler cfc free (astrazeneca uk ltd)
39102 budesonide 100micrograms/dose inhaler cfc free
39200 aerobec forte 250 autohaler (meda pharmaceuticals ltd)
39879 budesonide 200micrograms/dose inhaler cfc free
40057 pulmicort 200micrograms/dose inhaler cfc free (astrazeneca uk ltd)
40177 ipratropium bromide 250microgram/ml nebuliser liquid (hillcross pharmaceuticals ltd)
40218 azithromycin 500mg tablets (teva uk ltd)

For peer review only

40599 salbutamol 5mg/2.5ml nebuliser liquid unit dose steripoule vials (galen ltd)
40637 ipratropium 250micrograms/1ml nebuliser liquid unit dose steripoule vials (galen ltd)
40655 salbuvent 100microgram/actuation inhalation powder (pharmacia ltd)
40709 salbutamol 2.5mg/2.5ml nebuliser liquid unit dose vials (a a h pharmaceuticals ltd)
40832 ipratropium 500micrograms/2ml nebuliser liquid unit dose steripoule vials (galen ltd)
41269 beclometasone 400 cyclocaps (teva uk ltd)
41412 beclometasone 400micrograms/actuation inhaler
41548 salbutamol 2mg tablets (approved prescription services ltd)
41549 salbutamol 2mg tablet (c p pharmaceuticals ltd)
41691 salbutamol 2mg/5ml oral solution sugar free (sandoz ltd)
41832 monovent 1.5mg/5ml syrup (sandoz ltd)
42103 tulobuterol 1mg/5ml sugar free syrup
42279 salbutamol 2.5mg/2.5ml nebuliser liquid unit dose steripoule vials (galen ltd)
42497 salbutamol 8mg tablet
42511 aminophylline 25mg/ml injection (celltech pharma europe ltd)
42830 ventolin 100micrograms/dose evohaler (glaxosmithkline uk ltd)
42858 ventolin 200micrograms/dose accuhaler (glaxosmithkline uk ltd)
42867 terbutaline 1.5mg/5ml oral solution (sandoz ltd)
42886 bricanyl 500micrograms/dose turbohaler (astrazeneca uk ltd)
42910 aminophylline 250mg/10ml solution for injection ampoules (martindale pharmaceuticals ltd)
42928 flixotide 100micrograms/dose accuhaler (glaxosmithkline uk ltd)
42985 flixotide 50micrograms/dose accuhaler (glaxosmithkline uk ltd)
42994 flixotide 250micrograms/dose accuhaler (glaxosmithkline uk ltd)
43046 salipraneb 0.5mg/2.5mg nebuliser solution 2.5ml ampoules (arrow generics ltd)
43074 flixotide 500micrograms/dose accuhaler (glaxosmithkline uk ltd)
43085 bricanyl 5mg/2ml respules (astrazeneca uk ltd)
43400 clamelle 500mg tablets (actavis uk ltd)
43544 prednisone 5mg tablet (knoll ltd)
43738 indacaterol 150microgram inhalation powder capsules with device
43794 nebusal 7% inhalation solution 4ml ampoules (forest laboratories uk ltd)
43870 sodium chloride 7% inhalation solution 4ml ampoules
43893 onbrez breezhaler 150microgram inhalation powder capsules with device (novartis pharmaceuticals uk ltd)
44064 onbrez breezhaler 300microgram inhalation powder capsules with device (novartis pharmaceuticals uk ltd)
44173 roflumilast 500microgram tablets
44380 prednisone 1mg modified-release tablets
44431 daxas 500microgram tablets (takeda uk ltd)
44713 salbutamol 100microgram/inhalation inhalation powder (celltech pharma europe ltd)
45302 prednisolone 5mg tablet (biorex laboratories ltd)
45610 indacaterol 300microgram inhalation powder capsules with device
45863 salbutamol 5mg/2.5ml nebuliser liquid (generics (uk) ltd)
46157 beclometasone 200 cyclocaps (teva uk ltd)
46551 salbutamol 100microgram/inhalation inhalation powder (neo laboratories ltd)
46695 azithromycin 500mg tablet (hillcross pharmaceuticals ltd)
49016 sodium chloride 7% inhalation solution 4ml vials
49045 nebusal 7% inhalation solution 4ml vials (forest laboratories uk ltd)
49530 azithromycin 200mg/5ml oral suspension (sandoz ltd)
50508 mucodyne 250mg/5ml syrup (sigma pharmaceuticals plc)
52029 zithromax 250mg capsules (mawdsley-brooks & company ltd)
53303 carbocisteine 375mg capsules (arrow generics ltd)

For peer review only

Spirometry codes

medcode	readterm
6118	spirometry
10336	spirometry reversibility
10337	spirometry screening
10420	spirometry reversibility negative
10492	spirometry reversibility positive
13683	referral for spirometry
14453	forced expiratory volume - fev
19428	chronic obstructive pulmonary disease excluded by spirometry
26241	spirometry indicated
29015	spirometry
45993	incentive spirometry
102522	post bronchodilator spirometry
6091	Percent predicted FEV1
8512	FEV1/FVC percent
10320	Forced expired volume in 1 second
11078	FEV1/FVC > 70% of predicted
14455	FEV1/FVC ratio normal
14456	FEV1/FVC ratio
19830	FEV1 after bronchodilation
19832	FEV1/FVC ratio after bronchodilator
23237	FEV1 before bronchodilation
23285	FEV1/FVC ratio abnormal
25083	FEV1/FVC < 70% of predicted
27141	FEV1/FVC ratio before bronchodilator
43040	FEV1 pre steroids
43041	FEV1 post steroids
58632	FEV1/FVC ratio pre steroids
58633	FEV1/FVC ratio post steroids
99777	Forced expired volume in 1 second reversibility
100391	Forced expired volume in 1 second percentage change
101079	Percentage predicted FEV1 after bronchodilation

Non-specific bronchitis codes / Frequent winter bronchitis

medcode	readterm
148	bronchitis unspecified
152	wheezy bronchitis
3243	chronic bronchitis
3480	bronchitis nos
4519	h/o: bronchitis
5798	chronic asthmatic bronchitis
5909	chronic wheezy bronchitis
7092	recurrent wheezy bronchitis
11150	mucopurulent chronic bronchitis
13247	fh: bronchitis
13262	fh: bronchitis/coad
14798	emphysematous bronchitis
15157	chronic bronchitis nos
15626	chronic catarrhal bronchitis
17359	chest infection - unspecified bronchitis
24248	mixed simple and mucopurulent chronic bronchitis
25603	simple chronic bronchitis
27819	obstructive chronic bronchitis

For peer review only

37959	fetid chronic bronchitis
40159	purulent chronic bronchitis
44525	obstructive chronic bronchitis nos
61118	simple chronic bronchitis nos
61513	mucopurulent chronic bronchitis nos
62913	[v]screening for chronic bronchitis or emphysema
66043	other chronic bronchitis
68066	other chronic bronchitis nos
202167	INFLUENZAL BRONCHITIS
207165	Acute streptococcal bronchitis
207166	Acute viral bronchitis unspecified
216109	Acute membranous bronchitis
216111	Acute bronchitis due to mycoplasma pneumoniae
216112	Acute bronchitis NOS
216141	Chest infection - unspecified bronchopneumonia
220234	BRONCHITIS FIBRINOUS ACUTE
225216	Acute pseudomembranous bronchitis
225241	Bronchitis NOS
234340	Acute bronchitis due to rhinovirus
239395	H/O: bronchitis
243349	Acute bronchitis and bronchiolitis
243350	Acute pneumococcal bronchitis
243351	Acute bronchitis due to echovirus
243389	Bronchitis unspecified
261710	Acute wheezy bronchitis
261711	Acute purulent bronchitis
261712	Acute croupous bronchitis
261713	Acute bronchitis due to respiratory syncytial virus
271007	Acute bronchitis due to coxsackievirus
280054	Acute fibrinous bronchitis
280055	Acute bronchitis due to parainfluenza virus
280082	Chest infection - unspecified bronchitis
289164	Acute bronchitis
289165	Acute neisseria catarrhalis bronchitis
289166	Subacute bronchitis unspecified
289233	[X]Acute bronchitis due to other specified organisms
293231	BRONCHITIS SUBACUTE
298435	Acute haemophilus influenzae bronchitis
298436	Acute bacterial bronchitis unspecified
298440	Acute bronchitis or bronchiolitis NOS
303952	BRONCHITIS ACUTE
303954	VIRAL BRONCHITIS
303963	BRONCHOPNEUMONIA
306525	BRONCHITIS PURULENT
306528	CATARRHAL BRONCHITIS
308788	Acute bronchitis

Respiratory symptom codes

Wheeze

medcode	readterm
152	wheezy bronchitis
173	wheezing
2210	[d]wheezing

For peer review only

2891	wheezing symptom
4836	nocturnal cough / wheeze
5861	o/e - expiratory wheeze
5909	chronic wheezy bronchitis
5978	acute wheezy bronchitis
7092	recurrent wheezy bronchitis
42317	increasing exercise wheeze
100954	[d]mild wheeze
101037	[d]moderate wheeze
101073	[d]severe wheeze
101421	[d]very severe wheeze
102480	on examination - inspiratory wheeze

Chronic cough

medcode	readterm
92	cough
292	chesty cough
1025	bronchial cough
1160	[d]cough
1234	productive cough nos
1273	c/o - cough
1612	chronic cough
3068	night cough present
3628	persistent cough
3645	coughing up phlegm
4070	morning cough
4836	nocturnal cough / wheeze
4931	dry cough
7706	productive cough -clear sputum
7707	cough symptom nos
7708	productive cough-yellow sputum
7773	productive cough -green sputum
16717	smokers' cough
22318	difficulty in coughing up sputum
29318	evening cough
43795	unexplained cough
60903	cough aggravates symptom
100333	cough on exercise

Exertional breathlessness

medcode	readterm
735	[d]breathlessness
1429	breathlessness
3092	[d]dyspnoea
5175	breathlessness symptom
5896	dyspnoea - symptom
6326	breathless - moderate exertion
7000	o/e - dyspnoea
7932	breathless - mild exertion
19426	mrc breathlessness scale: grade 3
19427	mrc breathlessness scale: grade 2
19429	mrc breathlessness scale: grade 5

For peer review only

19430 mrc breathlessness scale: grade 4
19432 mrc breathlessness scale: grade 1
21801 breathlessness nos
24889 breathless - strenuous exertion
31143 breathless - at rest
42287 borg breathlessness score: 6 severe (+)
53771 dyspnoea on exertion
57193 borg breathlessness score: 3 moderate
57759 borg breathlessness score: 2 slight
59860 borg breathlessness score: 4 somewhat severe
64049 borg breathlessness score: 5 severe
67566 borg breathlessness score: 9 very, very sev (almost maximal)
68707 borg breathlessness score: 1 very slight
70061 borg breathlessness score: 7 very severe
70818 borg breathlessness score: 0.5 very, very slight
72334 borg breathlessness score: 8 very severe (+)
101843 borg breathlessness score: 10 maximal
741 [D]Shortness of breath
2563 [D]Respiratory distress
2575 Short of breath on exertion
2931 Difficulty breathing
4822 Shortness of breath
5349 Shortness of breath symptom
9297 [D]Respiratory insufficiency
22094 Short of breath dressing/undressing
40813 Unable to complete a sentence in one breath

Sputum codes

medcode readterm
292 chesty cough
1025 bronchial cough
1234 productive cough nos
3645 coughing up phlegm
7706 productive cough -clear sputum
7708 productive cough-yellow sputum
7773 productive cough -green sputum
22318 difficulty in coughing up sputum
1251 [d]abnormal sputum
3727 sputum sent for c/s
8287 sputum sample obtained
8760 [d]positive culture findings in sputum
9807 sputum - symptom
14804 sputum appears infected
15430 [d]sputum abnormal - colour
16026 sputum examination: abnormal
18964 sputum clearance
20086 [d]sputum abnormal - amount
22318 difficulty in coughing up sputum
23582 [d]abnormal sputum nos
24181 sputum: mucopurulent
29294 sputum appears normal
30754 yellow sputum
30904 sputum sent for examination
31417 sputum - not infected
35577 sputum examination - general

For peer review only

36515 [d]abnormal sputum - tenacious
36880 green sputum
40201 sputum microscopy: nad
40202 mucoid sputum - o/e
42573 clear sputum
43270 sputum evidence of infection
43272 sputum examination nos
44214 [d]sputum abnormal - odour
49029 sputum gen. exam. nos
49694 sputum: organism on gram stain
52806 sputum: frothy/watery
54177 sputum: excessive - mucoid
61079 sputum inspection nos
91680 expectoration of induced sputum from respiratory tract
100484 volume of sputum
100524 moderate sputum
100629 white sputum
100647 copious sputum
100931 brown sputum
101782 profuse sputum
103209 grey sputum

QOF codes

H3... Chronic obstructive pulmonary disease
H31.. Chronic bronchitis
H310. Simple chronic bronchitis
H3100 Chronic catarrhal bronchitis
H310z Simple chronic bronchitis NOS
H311. Mucopurulent chronic bronchitis
H3110 Purulent chronic bronchitis
H3111 Fetid chronic bronchitis
H311z Mucopurulent chronic bronchitis NOS
H312. Obstructive chronic bronchitis
H3120 Chronic asthmatic bronchitis
H3121 Emphysematous bronchitis
H3123 Bronchiolitis obliterans
H312z Obstructive chronic bronchitis NOS
H313. Mixed simple and mucopurulent chronic bronchitis
H31y. Other chronic bronchitis
H31y1 Chronic tracheobronchitis
H31yz Other chronic bronchitis NOS
H31z. Chronic bronchitis NOS
H32.. Emphysema
H320. Chronic bullous emphysema
H3200 Segmental bullous emphysema
H3201 Zonal bullous emphysema
H3202 Giant bullous emphysema
H3203 Bullous emphysema with collapse
H320z Chronic bullous emphysema NOS
H321. Panlobular emphysema
H322. Centrilobular emphysema
H32y. Other emphysema
H32y0 Acute vesicular emphysema

For peer review only

H32y1 Atrophic (senile) emphysema
H32y2 MacLeod's unilateral emphysema
H32yz Other emphysema NOS
H32z. Emphysema NOS
H36.. Mild chronic obstructive pulmonary disease
H37.. Moderate chronic obstructive pulmonary disease
H38.. Severe chronic obstructive pulmonary disease
H39.. Very severe chronic obstructive pulmonary disease
H3A.. End stage chronic obstructive airways disease (v23)
H3y.. Other specified chronic obstructive airways disease
~~H3y0. Chronic obstructive pulmonary disease with acute lower respiratory infection~~ (v23)
~~H3y1. Chronic obstructive pulmonary disease with acute exacerbation, unspecified~~ (v23)
H3z.. Chronic obstructive airways disease NOS

GP Questionnaire

1. Do you think this patient has COPD? Yes / No / Uncertain
2. What was the diagnosis of COPD based on? (please circle all that apply)
   Smoking history symptoms spirometry other (please describe)
3. Has a respiratory physician seen the patient and confirmed a diagnosis of COPD?
4. Does the patient have any other respiratory condition?
   If yes, than what?

Table S1: The PPV and proportion of patients diagnosed with COPD who also had cardiovascular disease or asthma

Algorithm	Number with CVDx	Number confirmed COPD	PPV and 95% CI	Number with asthma	Number confirmed COPD	PPV and 95% CI
**COPD Code + spirometry + COPD medication**	20	17	85.0, 59.6-95.6	18	14	72.2, 45.3-89.1
**COPD Code + spirometry**	24	20	83.3, 61.1-94.1	20	14	70.0, 45.0-87.0
**COPD Code + COPD medication**	18	16	88.9, 61.1-97.6	16	9	56.3, 29.8-79.6
**COPD Code only**	18	16	88.9, 61.1-97.6	15	12	80.0, 48.8-94.4
**Bronchitis + COPD medication**	21	8	38.1, 19.1-61.7	27	4	14.8, 5.3-35.1
**Bronchitis only**	19	8	42.1, 21.1-66.5	23	6	26.1, 11.4-49.1
**Symptoms + spirometry**	15	9	60.0, 31.8-82.9	19	4	21.1, 7.3-47.3
**Symptoms only**	9	2	22.2, 3.9-67.0	23	2	8.7, 1.9-31.4

For peer review only

Table S2: The PPV and proportion of patients diagnosed with COPD who were current smokers

Algorithm	Number current smokers	Number confirmed COPD	PPV and 95% CI
**COPD Code + spirometry + COPD medication**	49	44	89.9, 77.1-95.8
**COPD Code + spirometry**	51	39	76.5, 62.5-86.4
**COPD Code + COPD medication**	55	46	83.6, 71.0-91.4
**COPD Code only**	47	41	87.2, 73.8-94.3
**Bronchitis + COPD medication**	67	34	50.7, 38.7-62.7
**Bronchitis only**	64	19	29.7, 19.6-42.3
**Symptoms + spirometry**	50	20	40.0, 27.1-54.5
**Symptoms only**	58	6	10.3, 4.6-21.7

Table S3: The PPV and proportion of patients diagnosed with COPD by COPD severity

Algorithm	GOLD 1 Number confirmed, (PPV and 95% CI)	GOLD 2 Number confirmed, (PPV and 95% CI)	GOLD 3 Number confirmed, (PPV and 95% CI)	GOLD 4 Number confirmed, (PPV and 95% CI)
**COPD Code + spirometry + COPD medication**	9, (69.2, 36.5-89.8)	34, (87.2, 71.8-94.8)	22, (100)	6, (100)
**COPD Code + spirometry**	8, (66.7, 32.9-89.1)	35, (83.3, 68.2-92.1)	20, (100)	3, (75, 4.1-99.5)
**COPD Code + COPD medication**	14, (82.4, 53.7-94.9)	32 (84.2, 68.1-93.0)	20, (95.2, 69.1-99.4)	6, (100)
**COPD Code only**	14, (82.4, 53.7-94.9)	37, (86.0, 71.5-93.8)	14, (93.3, 58.4-99.3)	3, (100)
**Bronchitis + COPD medication**	7, (36.8, 17.3-62.0)	20, (76.9, 55.6-89.9)	11, (91.7, 49.9-99.2)	2, (100)
**Bronchitis only**	3, (20.0, 5.6-51.2)	7, (63.6, 28.8-83.3)	10 (100)	1, (100)
**Symptoms + spirometry**	4, (57.1, 15.0-90.9)	19, (90.5, 66.0-97.9)	10 (90.9, 46.3-99.1)	1, (100)
**Symptoms only**	2, (40, 3.8-91.9)	6, (100)	0	0

For peer review only

Table S4: The PPV and proportion of patients diagnosed with COPD by timing of diagnosis

Algorithm	2004 to end 2008			Post 2008		
	Number in algorithm	Number confirmed COPD	PPV and 95% CI	Number in algorithm	Number confirmed COPD	PPV and 95% CI
COPD Code + spirometry + COPD medication	32	28	87.5, 69.8-95.5	53	48	90.6, 78.7-96.1
COPD Code + spirometry	14	11	78.6, 46.0-94.0	66	56	84.8, 73.7-91.8
COPD Code + COPD medication	27	23	85.2, 64.9-94.7	61	54	88.5, 77.4-94.5
COPD Code only	21	16	76.2, 51.7-90.5	68	61	89.7, 79.6-95.1
Bronchitis + COPD medication	94	42	44.7, 34.8-55.0	5	2	40.0, 3.8-91.9
Bronchitis only	83	22	26.5, 18.0-37.2	5	4	80.0, 11.1-99.2
Symptoms + spirometry	63	28	44.4, 32.4-57.1	22	9	40.9, 21.6-63.5
Symptoms only	64	10	15.6, 8.5-27.0	26	1	3.8, 0.47-25.4

For peer review only

STROBE Statement—checklist of items that should be included in reports of observational studies

	Item No	Recommendation
**Title and abstract**	1	(*a*) Indicate the study's design with a commonly used term in the title or the abstract
**Pg 1 and 2**		(*b*) Provide in the abstract an informative and balanced summary of what was done and what was found
**Introduction**		
Background/rationale **Pg 3**	2	Explain the scientific background and rationale for the investigation being reported
Objectives **Pg 4**	3	State specific objectives, including any prespecified hypotheses
**Methods**		
Study design **Pg 4**	4	Present key elements of study design early in the paper
Setting **Pg 4**	5	Describe the setting, locations, and relevant dates, including periods of recruitment, exposure, follow-up, and data collection
Participants **Pg 6**	6	(*a*) *Cohort study*—Give the eligibility criteria, and the sources and methods of selection of participants. Describe methods of follow-up<br>*Case-control study*—Give the eligibility criteria, and the sources and methods of case ascertainment and control selection. Give the rationale for the choice of cases and controls<br>*Cross-sectional study*—Give the eligibility criteria, and the sources and methods of selection of participants
		(*b*) *Cohort study*—For matched studies, give matching criteria and number of exposed and unexposed<br>*Case-control study*—For matched studies, give matching criteria and the number of controls per case
Variables **Pg 5,7**	7	Clearly define all outcomes, exposures, predictors, potential confounders, and effect modifiers. Give diagnostic criteria, if applicable
Data sources/ measurement **Pg 7**	8*	For each variable of interest, give sources of data and details of methods of assessment (measurement). Describe comparability of assessment methods if there is more than one group
Bias **Pg 6**	9	Describe any efforts to address potential sources of bias
Study size **Pg 8**	10	Explain how the study size was arrived at
Quantitative variables **Pg 7 and 8**	11	Explain how quantitative variables were handled in the analyses. If applicable, describe which groupings were chosen and why
Statistical methods **Pg 7 and 8**	12	(*a*) Describe all statistical methods, including those used to control for confounding
		(*b*) Describe any methods used to examine subgroups and interactions
		(*c*) Explain how missing data were addressed
		(*d*) *Cohort study*—If applicable, explain how loss to follow-up was addressed<br>*Case-control study*—If applicable, explain how matching of cases and controls was addressed<br>*Cross-sectional study*—If applicable, describe analytical methods taking account of sampling strategy

For peer review only - http://bmjopen.bmj.com/site/about/guidelines.xhtml

For peer review only

(*e*) Describe any sensitivity analyses

Continued on next page

For peer review only

For peer review only - http://bmjopen.bmj.com/site/about/guidelines.xhtml

**Results**		
Participants<br>**Pg 9**	13*	(a) Report numbers of individuals at each stage of study—eg numbers potentially eligible, examined for eligibility, confirmed eligible, included in the study, completing follow-up, and analysed
		(b) Give reasons for non-participation at each stage
		(c) Consider use of a flow diagram
Descriptive data<br>**Pg 9 and 10**	14*	(a) Give characteristics of study participants (eg demographic, clinical, social) and information on exposures and potential confounders
		(b) Indicate number of participants with missing data for each variable of interest
		(c) *Cohort study*—Summarise follow-up time (eg, average and total amount)
Outcome data<br>**Pg 11**	15*	*Cohort study*—Report numbers of outcome events or summary measures over time
		Case-control study—Report numbers in each exposure category, or summary measures of exposure
		Cross-sectional study—Report numbers of outcome events or summary measures
Main results<br>**Pg 10-12**	16	(*a*) Give unadjusted estimates and, if applicable, confounder-adjusted estimates and their precision (eg, 95% confidence interval). Make clear which confounders were adjusted for and why they were included
		(*b*) Report category boundaries when continuous variables were categorized
		(*c*) If relevant, consider translating estimates of relative risk into absolute risk for a meaningful time period
Other analyses<br>**Supplementary material**	17	Report other analyses done—eg analyses of subgroups and interactions, and sensitivity analyses
**Discussion**		
Key results<br>**Pg 12**	18	Summarise key results with reference to study objectives
Limitations<br>**Pg 15**	19	Discuss limitations of the study, taking into account sources of potential bias or imprecision. Discuss both direction and magnitude of any potential bias
Interpretation<br>**Pg 12-15**	20	Give a cautious overall interpretation of results considering objectives, limitations, multiplicity of analyses, results from similar studies, and other relevant evidence
Generalisability<br>**Pg 15**	21	Discuss the generalisability (external validity) of the study results
**Other information**		
Funding<br>**Pg 17**	22	Give the source of funding and the role of the funders for the present study and, if applicable, for the original study on which the present article is based

*Give information separately for cases and controls in case-control studies and, if applicable, for exposed and unexposed groups in cohort and cross-sectional studies.

Note: An Explanation and Elaboration article discusses each checklist item and gives methodological background and published examples of transparent reporting. The STROBE checklist is best used in conjunction with this article (freely available on the Web sites of PLoS Medicine at http://www.plosmedicine.org/, Annals of Internal Medicine at http://www.annals.org/, and Epidemiology at http://www.epidem.com/). Information on the STROBE Initiative is available at www.strobe-statement.org.

For peer review only
